# WWOX gene is associated with HDL cholesterol and triglyceride levels

**DOI:** 10.1186/1471-2350-11-148

**Published:** 2010-10-14

**Authors:** María E Sáez, Antonio González-Pérez, María T Martínez-Larrad, Javier Gayán, Luis M Real, Manuel Serrano-Ríos, Agustín Ruiz

**Affiliations:** 1 Departamento de Genómica Estructural. Neocodex. C/. Charles Darwin 6, Acc. A, 41092 Sevilla, Spain; 2 Departamento de Medicina Interna II. Hospital Clínico Universitario San Carlos. CIBER de Diabetes y Enfermedades Metabólicas Asociadas (CIBERDEM). C/Profesor Martín Lagos s/n, 28040, Madrid, Spain

## Abstract

**Background:**

Altered lipid profile, and in particular low HDL and high triglyceride (TG) plasma levels, are within the major determinants of cardiovascular diseases. The identification of quantitative trait loci (QTL) affecting these lipid levels is a relevant issue for predictive purposes. The *WWOX *gene has been recently associated with HDL levels. This gene is located at chromosome 16q23, a region previously linked to familial combined hyperlipidemia (FCHL) and HDL. Our objective is to perform a genetic association analysis at the *WWOX *gene region with HDL, TG and TG/HDL ratio.

**Methods:**

A quantitative association analysis performed in 801 individuals selected from the Spanish general population.

**Results:**

For HDL levels, two regions of intron 8 display clustering of positive signals (p < 0.05) but none of them was associated in the haplotypic analysis (0.07 ≤ p ≤ 0.165). For TG levels not only intron 8 but also a 27 kb region spanning from the promoter region to intron 4 are associated in this study. For the TG/HDL genetic association analysis, positive signals are coincident with those of the isolated traits. Interestingly, haplotypic analysis at the 5' region showed that variation in this region modified both HDL and TG levels, especially the latter (p = 0.003).

**Conclusions:**

Our results suggest that *WWOX *is a QTL for both TG and HDL.

## Background

Altered lipid profile is one of the major determinants of cardiovascular disease, which is the first cause of death in the developed countries. Unhealthy diet and low physical activity both contribute to the appearance of dyslipidemia, but blood lipid profile is also highly heritable. In addition to several mendelian forms of hyperlipemia and hypertriglyceridemia, dyslipidemia is commonly a complex disease or group of diseases with an estimated heritability ranging from 25 to 80% [1]. A recent study performed in 1,275 coronary artery disease patients derived from the *Regensburg Myocardial Infarction Family Study *has described heritabilities of 27-48% for HDL cholesterol and 21-44% for LDL cholesterol [2].

The genetic variation in genes such as lipoprotein lipase (LPL), hepatic lipase (LIPC), the LDL receptor (LDLR), the ABCA1 transporter or diverse apolipoproteins, has been found to influence blood lipid levels [3-8]. Lately, the rapid spread of genome-wide association studies has allowed not only the confirmation of previously described associations, but also the identification of many quantitative trait loci (QTL) for lipid levels across the genome [9-15]. One of these loci has been recently reported for HDL [16] and involves the WW-domain-containing oxidoreductase (*WWOX*) gene (MIM 605131). *WWOX *gene is a large gene spanning about 1.1 Mb and located within a region previously linked to HDL and familial combined hyperlipidemia (FCHL), a hereditary disorder characterized by the elevation of both cholesterol and triglycerides (TGs) in the blood [17-21]. *WWOX *encodes a protein which contains 2 WW domains and a short-chain dehydrogenase/reductase domain (SRD). The highest normal expression of this gene is detected in hormonally regulated tissues such as testis, ovary, and prostate [22]. This expression pattern and the presence of an SRD domain suggest a role for this gene in steroid metabolism. In fact, WWOX is implicated in tumorigenesis [23-25], a pathological process highly dependent on cholesterol metabolism. In fact, WWOX knockout mouse model exhibits hypotriglyceridemia and hypocholesterolemia among other metabolic disturbances [26]. A recent report by Vasan et al. [27] associated the rs2059238 polymorphism, located at intron 5, with left ventricular wall thickness in individuals with coronary artery disease (CAD).

To assess the effect of this gene in our population, we have analysed the *WWOX *gene region for association with TGs, HDL and TG/HDL ratio. The TG and HDL levels are inversely correlated and their metabolism are closely interrelated. Therefore, the TG/HDL ratio has been suggested to be a powerful estimator of cardiovascular disease risk [20,28]. We have analysed for association with HDL, TG and TG/HDL, 1045 polymorphisms genotyped from the Affymetrix 250 k NspI assay or imputed with MACH 1 software using CEU HapMap phased haplotype data [29].

## Methods

### Study design

This study comprises 801 non related Caucasian men (n = 433, 54.05%) and women (n = 368, 45.95%) who were recruited by a simple random sampling approach from a cross-sectional population-based epidemiological survey in Spain, aimed at investigating the prevalence of anthropometric and physiological parameters related to obesity and other components of MS [30,31]. Participants with previous diagnosis of type 1 diabetes were excluded from the study.

All participants gave their written consent to participate in the study. The study protocol was approved by the Ethics Committee of the Hospital Clínico San Carlos of Madrid.

### Measurements

#### Biochemical determinations

After an overnight fasting period, 20 ml of blood were obtained from an antecubital vein without compression. HDL and TG levels were determined by enzymatic methods using commercial kits from Boehringer Mannheim.

#### Genotypes

Genotypic data were derived from a genome wide scan performed with the 250 k NspI Affymetrix chip. Genotyping was done according to manufacturer's instructions.

#### Genetic quality control

Of the 253 polymorphism included in the Affymetrix chip located at the *WWOX *genomic region (transcribed region ± 2 kb), only 175 passed the quality control filtering (at least 90% of individuals genotyped, minor allele frequency (MAF) ≥ 0.05, and Hardy-Weinberg equilibrium p > 0.05).

### Statistical analysis

To compare our results with those previously published, the 175 genotyped SNPs were used to impute untyped polymorphism in the region using MACH 1 software (http://www.sph.umich.edu/csg/abecasis/MACH) [29] and CEU HapMap phased haplotypic data (http://www.HapMap.org), since no significant differences were observed between the Spanish and the HapMap populations (Additional files 1-2). According to the software recommendations, only those SNPs with a r^2 ^> 0.30 were selected for the association analysis (870 polymorphisms). Quality metrics for imputed genotypes are included in Additional file 3. Finally, 1045 SNPs (151 directly genotyped and 894 imputed, all of them with MAF ≥ 0.10) were analysed for association with HDL, TGs and HDL/TG ratio using the linear regression procedure included in the PLINK software [32]. 39 individuals under lipid lowering medications were excluded from the genetic association analyses. All the studies were adjusted by sex, age, BMI, smoking (defined as present or past history of smoking of at least five cigarettes per day for a minimum of 5 years), alcohol consumption (defined as a daily intake of more than 1 drink, equivalent to 10 g of pure ethanol), and physical activity (physical activity was defined as light or moderate activity for a minimum of 60 minutes per session at least three times a week). Empirical p values were obtained using the adaptative permutation method implemented in PLINK [32]. We have not applied multiple comparison correction because we consider it over-conservative for the replication of a previously described gene specially given that most genotypes analysed in this report are derived from a small number of observed genotypes; a p value under 0.05 is interpreted as significant.

Linkage disequilibrium (LD) patterns at the *WWOX *gene were analysed using Haploview software [33].

For haplotypic association analysis, we have also employed PLINK v1.06 software [32]. Only haplotypes with a population frequency over 5% were retained for analysis.

## Results

The characteristics of the study population are described in Table 1.

**Table 1:** Characteristics of the study population

	*All* *N = 801*	*Men* *N = 433*	*Women* *N = 368*	*Men vs women* *P value*
***Age (years)***	51.90 (8.81)	51.57 (9.24)	52.30 (8.28)	0.242

***BMI (kg/m2)***	27.44 (4.29)	27.11 (3.71)	27.84 (4.87)	0.017

***Triglycerides (mg/dl)***	104.81 (73.23)	116.36 (83.38)	91.21 (56.26)	1.09 × 10^-6^

***HDL (mg/dl)***	50.57 (20.78)	46.08 (19.09)	55.86 (21.45)	1.72 × 10^-11^

***Smokers (%)***	28.6%	42.5%	12.2%	1.34 × 10^-14^

***Alcohol consumers (%)***	66.0%	83.4%	45.7%	2.97 × 10^-14^

***Physical active (%)***	46.3%	45.3%	47.6%	0.523

For quantitative traits, values represent means and, in brackets, standard deviations (SD). For the statistical differences p values (Anova test for quantitative traits and exact test for dichotomic traits) are given.

**Figure 1:**
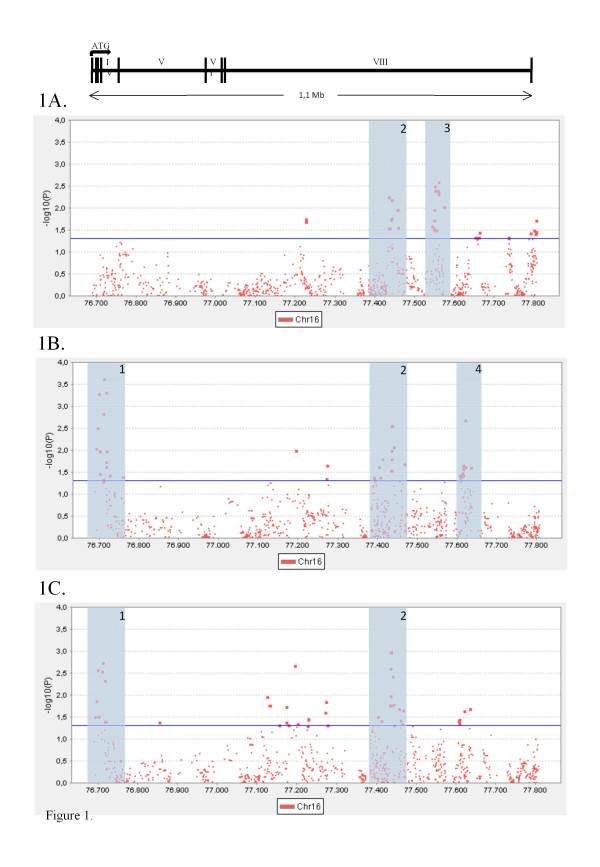
**Graphical representation of the association analysis of the *WWOX *gene with HDL (Figure 1A), triglycerides (Figure 1B) and TG/HDL index (Figure 1C)**. Dots symbolize the logarithm of the p value at each marker. In the top of the Figure a scaled representation of the WWOX gene structure is shown. Shaded numbered zones point out the regions referred in the text as regions 1-4.

For all the traits analysed, we have identified four regions displaying clustering of positive signals (Additional files 4, 5, 6, 7). They have been numbered on the basis of their chromosomal positions from 5' to 3' (Figure 1): region 1 from 76,689,775 to 76,716,533 bp (NCBI36/hg18), region 2 from 77,433,428 to 77,441,867 bp (NCBI36/hg18), region 3 from 77,538,855 to 77,563,088 bp (NCBI36/hg18), and region 4 from 77,605,536 to 77,620,657 bp (NCBI36/hg18). The complete list of the results from the association analysis can be accessed online (Additional files 8, 9, 10).

For HDL levels, two regions concentrate the positive signals, both of them in the *WWOX *gene intron 8 (Figure 1A and Additional file 8). The first of them, comprises linked SNPs in the region 77,433,428 - 77,441,867 bp (rs7405423-rs4145518, region 2); the adjacent LD block (77,442,683-77,457,346 bp, rs8060856-rs2656634), partially linked with it (D' = 0.92, Additional file 5), also shows associated markers. The strongest associations in the region were observed for the rs4888865 (p = 0.006, β = 0.96) and the rs4145518 (p = 0.007, β = 1.03) polymorphisms. The second associated region, also in the 8^th ^intron, is distal to the previous one (77,538,855-77,563,088 bp, region 3) and comprises a large LD block of 42 markers (rs11643794-rs1110894, Additional file 6) of which the rs2047927 showed the highest significance (p = 0.003, β = -2.25).

For TG quantitative association analysis, again two regions in the intron 8 showed clustering of positive signals (Figure 1b and Additional file 9). One of these regions was the same LD block at the region 77,433,428 - 77,441,867 bp associated with HDL (region 2), but the signal was limited in this case to the seven first highly linked markers (rs7405423-rs7501409, D' = 1). The other associated region in intron 8 extends from 77,605,536 to 77,620,657 bp (rs12598471-rs2550698, region 4, Additional file 7), showing a peak at the rs11645630 (p = 0.002, β = -11.14). However, the highest signals in this study are located at 76,689,775-76,716,533 bp (rs10220974-rs1076514, region 1, Additional file 4), a broad region that extends from the promoter to the 4^th ^intron, with the largest association at rs11645006 (p = 0.0002, β = -13.81).

In the association study of the TG/HDL index, regions 1 (from 76,689,775 to 76,716,533 bp, promoter region) and 2 (intron 8, from 77,433,428 to 77,441,867 bp) huddle associated SNPs (Figure 1C and Additional file 10). For the region 1, that is associated with TGs but not with HDL, p values are smaller than those obtained for TGs. On the other hand, in the region 2, which is associated with both HDL and TGs, the significance is increased with respect to the individual phenotypes. Additionally, four markers of those included in region 4 (intron 8, from 77,605,536 to 77,620,657 bp) exceed the 0.05 p value threshold.

### Haplotypic analysis

We have also performed a haplotypic association analysis in the regions associated in the genotypic study (Table 2). The global haplotypic effect at region 1 (76,689,775-76,716,533 bp) was significant for all the traits analysed (0.0029 ≤ p ≤ 0.0180), despite that allelic analysis did not show any associated marker with HDL. For region 2 (77,433,428 - 77,441,867 bp), only HDL was not significant. For regions 3 (77,538,855-77,563,088 bp) and 4 (77,605,536-77,620,657 bp), only the analysis of TGs at region 4 was significant. Selection of tag SNPs did not improve the results of the association analysis (data not shown).

**Table 2:** Haplotypic analysis at the four *WWOX *regions selected in the genotypic analysis

	HAPLOTYPE	FREQ.	TGs	HDL	TGs/HDL
**REGION 1**	CTTCCTCCCGCGCAGA	0.14	(-ref)	(-ref)	(-ref)
	
	TTCGATCTTCGATGGG	0.12	11.44 (0.09; 22.80)	0.38 (-2.03; 2.78)	0.28 (-0.05; 0.59)
	
	CCTGAGTTTCGACAGG	0.11	10.05 (-1.96; 22.10)	-2.14 (-4.68; 0.41)	0.34 (0.01; 0.68)
	
	CCTGAGTTTCGATGGG	0.17	15.32 (5.27; 25.40)	0.41 (-1.72; 2.54)	0.39 (0.10; 0.67)
	
	CTCGATTTTCCGCACA	0.09	2.85 (-10.30; 16.00)	0.60 (-2.18; 3.38)	0.003 (-0.37; 0.38)
	
	CCTGAGTTTCCACAGG	0.07	7.82 (-6.54; 22.20)	4.24 (1.2; 7.28)	0.03 (-0.38; 0.43)
	
	CTTCCTCTTCCATAGG	0.05	10.54 (-5.77; 27.00)	2.73 (-0.75; 6.19)	0.23 (-0.23; 0.69)
	
	CTTCCTCTTCCGCACA	0.07	-15.76 (-30.20;-1.29)	2.04 (-1.02; 5.11)	-0.42 (-0.83; -0.01)
	
	*GLOBAL HAPLOTYPIC EFFECT*		F = 3.13 p = **0.003**	F = 2.435 p = **0.018**	F = 2.836 p = **0.006**

**REGION 2**	TGACAAGACATG	0.09	(-ref)	(-ref)	(-ref)
	
	CATAAAGAGGTA	0.07	1.50 (-13.50; 16.50)	-2.62 (-5.79; 0.56)	0.09 (-0.33; 0.52)
	
	CATACGCGGGTA	0.52	12.54 (3.34; 21.70)	-2.43 (-4.37; -0.48)	0.41 (0.15; 0.66)
	
	CATACGCGGGCA	0.19	8.36 (-2.86; 19.60)	-3.33 (-5.7; -0.95)	0.31 (-0.01; 0.62)
	
	CATACGCGCATA	0.05	20.08 (3.23; 36.90)	-1.81 (-5.37; 1.76)	0.67 (0.20; 1.15)
	
	*GLOBAL HAPLOTYPIC EFFECT*		F = 2.72 p = **0.028**	F = 2.17 p = 0.070	F = 3.50 p = **0.007**

**REGION 3**	GACGCTGCACTAGCGATCATTAGTACCAGACATGACAGCCCGC	0.28	(-ref)	(-ref)	(-ref)
	
	ATGTTCAGAGTAAGCGAGGTCACCGCGGCGGCCAGGGATATAT	0.31	-3.62 (-11.30; 4.04)	0.72 (-0.89; 2.34)	-0.13 (-0.34; 0.09)
	
	GAGGTTGGGCAGGCCATCACTGCTACCAGACCTGAGAACACAC	0.07	-12.22 (-25.10; 0.67)	-2.07 (-4.78; 0.65)	-0.26 (-0.62; 0.11)
	
	*GLOBAL HAPLOTYPIC EFFECT*		F = 1.89 p = 0.151	F = 1.81 p = 0.165	F = 1.35 p = 0.261

**REGION 4**	GCTCTCCCGAAGGCCAGAGAACACCTAGACGATT	0.12	(-ref)	(-ref)	(-ref)
	
	GCTCTCCCTAGGGTAGAAAGCATTCCCCAAGATT	0.06	-11.44 (-27.80; 4.88)	0.54 (-2.93; 4.02)	-0.32 (-0.79; 0.14)
	
	GTACGAGCGAAGGCCAGAGAACACCTAGACGATT	0.18	6.45 (-3.59; 16.50)	-1.09 (-3.24; 1.04)	0.20 (-0.09; 0.48)
	
	GTACGAGCTAGGGTAGAAAGCATTCCCCAAGATT	0.18	-6.28 (-15.4; 2.84)	-0.30 (-2.24; 1.64)	-0.09 (-0.35; 0.17)
	
	GTACGAGCGGGAACCGGGAAACACCCACACAATT	0.07	11.58 (-1.97; 25.10)	-1.02 (-3.90; 1.87)	0.28 (-0.11; 0.66)
	
	*GLOBAL HAPLOTYPIC EFFECT*		F = 2.61 p = **0.0347**	F = 0.43 p = 0.786	F = 1.99 p = 0.0939

Region 1 (Chr.16: 76,689,775-76,716,533 bp): rs10220947, rs4887935, rs7192129, rs12931172, rs8045450, rs2287973, rs2287972, rs16947127, rs16947129, rs12917833, rs10492874, rs11645006, rs2042356, rs1079569, rs12920698, rs1076514.

Region 2 (Chr.16: 77,433,428 - 77,441,867 bp): rs7405423, rs7405283, rs9922613, rs4888865, rs6420411, rs1543296, rs7498176, rs7501409, rs12447303, rs7190122, rs11643930, rs4145518.

Region 3 (Chr.16: 77,538,855-77,563,088 bp): rs11643794, rs11642227, rs10871357, rs4888887, rs1995549, rs8063186, rs11648482, rs7205567, rs16949032, rs17709129, rs17709147, rs17635945, rs11150119, rs9319530, rs13334115, rs10871358, rs12596756, rs12925319, rs16949036, rs12447067, rs9941223, rs7189021, rs17636170, rs8052880, rs4600477, rs4888888, rs4888889, rs8052893, rs11150120, rs13339083, rs4888892, rs13330742, rs16944152, rs2047927, rs5024396, rs17636262, rs2062900, rs8057659, rs12716865, rs1467067, rs11860206, rs1110891, rs1110894.

Region 4 (Chr.16: 77,605,536 - 77,620,657 bp): rs12598471, rs2550725, rs1862841, rs16949214, rs2550724, rs12447246, rs2550723, rs16949222, rs2113305, rs7185820, rs8064141, rs16949238, rs16949240, rs2656653, rs2550718, rs2550717, rs2550716, rs16949251, rs8047597, rs2550711, rs8047300, rs2550710, rs7199119, rs1808447, rs905781, rs8053936, rs2550702, rs2550701, rs2656649, rs11645630, rs16949276, rs2656647, rs2656646, rs2550698.

## Discussion

We have reported association of four *WWOX *gene regions with HDL and TG levels. One of them is located at the 5' end of the gene, from the promoter to the intron 4 (region 1) whereas the remaining regions are placed at the intron 8 of the gene, a region previously associated with HDL levels by Lee et a. [16]. Haplotypic analysis pointed out that the allelic combinations at region 1 significantly modify HDL, TG levels and the TG/HDL index, despite that no individual SNP associations were found for HDL in this region. Moreover, none of the intron 8 regions are associated with HDL in the haplotypic analysis, although region 2 shows a trend for association (p = 0.07). Regions 2 and 4 are associated with TG levels and only region 2 with the TG/HDL index.

Our results suggest that the *WWOX *gene is associated with HDL and TGs levels in our study population. From a functional point of view, the 5' region of the gene with both TG and HDL is highly interesting, since includes regulatory regions of the gene and two 44 bp insertion/deletion polymorphisms. For the *WWOX *gene, there have been eight different mRNAs described (7 alternatively spliced variants and 1 unspliced form) and three probable alternative promoters [34]. Of eight transcripts, six spliced and the unspliced mRNAs putatively encode good proteins. It is tempting to speculate that genetic variations in this region could modify the expression of the *WWOX *gene. Functional analyses are needed to answer this hypothesis.

The second region identified in this study is, of the three regions identified in intron 8, the one nearest to the 5' boundary of this uncoding region. It is associated with all the traits analysed, although for HDL, it didn't reach the statistical significance threshold. This region is in high LD with the rs2548861 polymorphism associated by Lee et al. (D' = 0.72), which is located upstream of this region at approximately 200 kb.

Two other regions of the large intron 8 (around 800 kb long) display clustering of associated SNPs with HDL or TG. The global haplotypic analyses for these two regions were not significant, indicating that the observed associations in the genotypic analysis seem to be related with individual polymorphic effects rather than to haplotypic blocks.

Genotype imputation methods are now being commonly used in the analysis of genome-wide association studies. Imputation enables researchers to pool and/or compare data between studies conducted using different SNP sets. It is also possible to fine map associated regions. Imputation methods work by combining a reference panel of individuals genotyped at a dense set of polymorphisms with a study sample collected from a genetically similar population and genotyped at a subset of these SNPs. Usually, HapMap panels are employed as the reference sample. It has raised the question about how well these panels represent the genetic variation in other populations that, for historical and demographic reasons, are more distant from the reference population. Two recent papers have analyzed the genomic diversity in European populations and revealed that the level of genetic differentiation within Europe is small [35] while the complex origin of the Spanish population is reflected in a greater haplotypic diversity than Northern/Western Europeans, although this divergence is not statistically significant [36]. These reports are in accordance with our observation that the Spanish population is largely homogeneous and in general similar, to the CEU HapMap sample in terms of MAF and LD patterns, notwithstanding that our population has more blocks and smaller on average [37]. Specifically at the *WWOX *region, no significant differences were observed between the Spanish and the CEU HapMap sample (Additional files 1-2). Regarding accuracy of imputation, we have recently imputed the complete genome using the 250 k NspI chip information and CEU HapMap sample with an overall concordance of 96.7% between the genotyped and imputed genotypes revealed by the use of the mask function. These findings are in accordance with the results presented by Huang et al. [38] that described high imputation accuracy for all the European populations examined (including a Spanish sample) with the CEU HapMap reference panel. Another supporting argument for the validity of the imputed genotypes is that the proportion of genotyped:imputed SNPs is 1:6. The associated SNPs are an admixture of both of them in approximately the same proportion: 1:8.7 for HDL, 1:6.6 for TGs and 1:8.4 for TG/HDL, with p values greater than 0.5 for the comparison of the distribution of associated/not associated SNPs in both genotyped and imputed classes.

In our study, we failed to replicate the association of the rs2548861 with HDL. This polymorphism has been imputed in our study population, and inaccurate imputation could be the cause for the absence of association, although it is unlikely for the reasons exposed above. We have estimated the effect size (ES) of this SNP in our study using the same method employed by Lee based on the t statistics (ES = t × sqrt [[n0 + n1]/[n0 × n1]], ni = number of participants coded as i), that represents the proportion of one-standard-deviation change in HDL. In our study population, the ES for the rs2548861 is -0.08, exactly the same raw score obtained by Lee et al. for the METSIM cohort, which includes more than 4,000 males. We estimate that our study is underpowered for SNPs with effect sizes below 0.14, so we can not rule out power issues. However, in the report by Lee et al [16] the effect of the rs2548861 was identified in familial cases of dyslipidemia and validated in two large population-based samples of Finnish samples, but the authors also failed to replicate this association in three GWAS dataset: the Diabetes Genetics Initiative (DGI) (1,464 cases of type 2 diabetes and 1,467 non-diabetic control individuals from Scandinavia), the Finland-United States Investigation of NIDDM Genetics (FUSION, 1,161 Finnish type 2 diabetes cases, 1,174 normal glucose tolerant controls, and 122 offspring of case/control pairs) and the Sardinia study of aging-associated variables cohorts (4,305 related individuals). Lee et al. pointed out that part of the initial linkage signal remains unexplained by rs2548861, suggesting that additional variants in the WWOX region influence lipid levels. In fact, the rs2667590 polymorphism, located also in the intron 8 but not linked to the rs2548861 polymorphism, has been also associated with HDL levels [12].

In our study, a new region in the WWOX gene, the promoter, is shown to be associated with both HDL and TG levels, spanning the susceptibility region to the proximal region of the long arm of chromosome 16. 10 Mbp away from the WWOX gene, at 16q22, several recent reports have identified different polymorphisms at the lecithin-cholesterol acyltransferase (*LCAT*) gene in association with HDL [12-15]. It has been recently reported the existence of synthetic associations, that is, combinations of several low frequency variants across large regions of the genome. These synthetic associations can span up to 10 Mb and encompass several LD blocks [39,40]. It is then possible that the *WWOX *and the *LCAT *regions are components of the same synthetic haplotype which contributes to the regulation of blood lipid levels.

The work presented in this article, has identified new putative SNPs associated, not only with HDL, but also with triglycerides and the TG/HDL index, a powerful estimator or cardiovascular risk. In addition to the previously described intron 8, we have identified the promoter region as a new region influencing both HDL and TG levels. We cannot rule out that some of the observed associations could have arisen by chance, reason why additional, independent replications are needed.

## Conclusions

In conclusion, the information derived from the genome wide genotyping combined with that provided for the imputation procedure, has allowed us to fine map the *WWOX *region. We agree with Lee et al. [16] on the relevance of *WWOX *intron 8 in HDL and we extend it to TG levels. In addition, we have identified the 5' region of the gene as another putative source of HDL and TG variance at least in this Spanish sample. The *WWOX *gene is therefore a promising target for future research in cardiovascular diseases and risk factors.

## Competing interests

The authors declare that they have no competing interests.

## Authors' contributions

MES: analysed and interpreted data and draft the manuscript.

AGP, JG and AR: have contributed to conception and design of the study and revised the manuscript.

LMR, MTML and MSR: have contributed to the acquisition and interpretation of data and revised the manuscript.

All authors have read and approved the manuscript.

## Pre-publication history

The pre-publication history for this paper can be accessed here:

http://www.biomedcentral.com/1471-2350/11/148/prepub

## Supplementary Material

Additional file 1 Figure S1: LD map of *WWOX *gene in the Spanish population Click here for file

Additional file 2 Figure S2: LD map of *WWOX *gene in the CEU HapMap reference panel Click here for file

Additional file 3 Table S1: Quality metrics for imputed SNPs Click here for file

Additional file 4 Figure S3: LD map at *WWOX *region 1 (Chr.16: 76,689,775-76,716,533 bp) Click here for file

Additional file 5 Figure S4: LD map at *WWOX *region 2 (Chr.16: 77,433.428-77,441.867 bp) Click here for file

Additional file 6 Figure S5: LD map at *WWOX *region 3 (Chr.16: 77,538.855-77,563.088 bp) Click here for file

Additional file 7 Figure S6: LD map at *WWOX *region 4 (Chr.16: 77,605.536-77,620.657 bp) Click here for file

Additional file 8 Table S2: Association analysis of the WWOX gene markers with HDL Click here for file

Additional file 9 Table S3: Association analysis of the WWOX gene markers with triglycerides Click here for file

Additional file 10 Table S4: Association analysis of the WWOX gene markers with TG/HDL index Click here for file
