# Supplementary figures and images for: WWOX gene is associated with HDL cholesterol and triglyceride levels
Source: BMC Med Genet. 2010 Oct 14;11:148. doi: 10.1186/1471-2350-11-148 (PMC2967537; doi:10.1186/1471-2350-11-148)

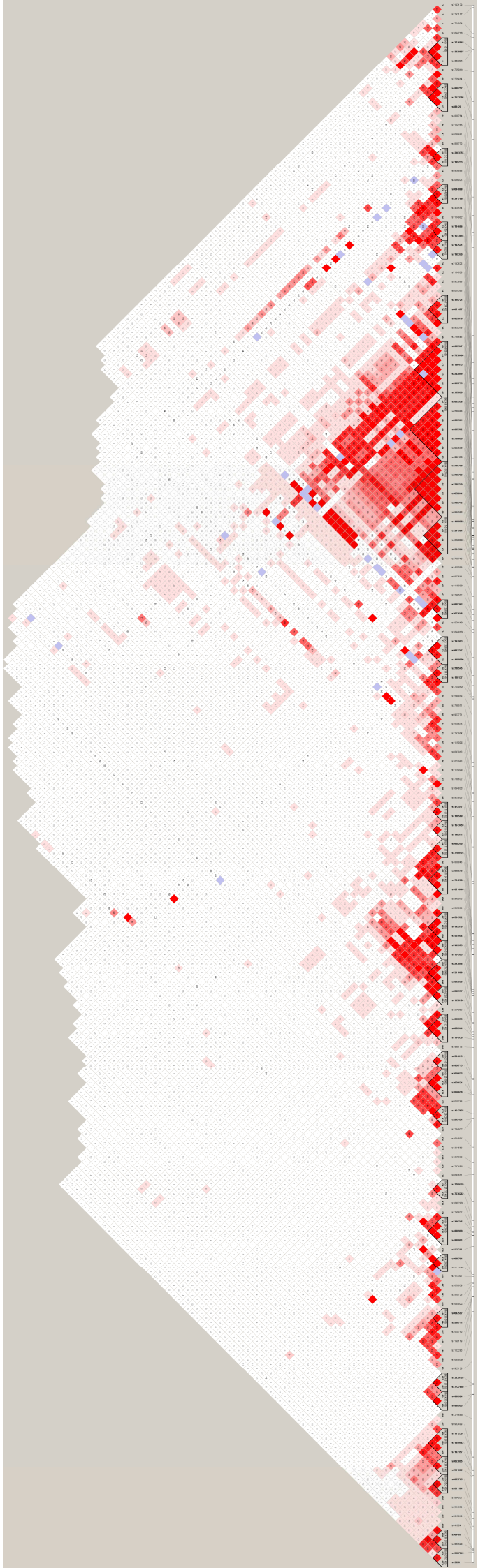

Supplement: Additional file 1 — Figure S1: LD map of WWOX gene in the Spanish population [file 1471-2350-11-148-S1.PDF]

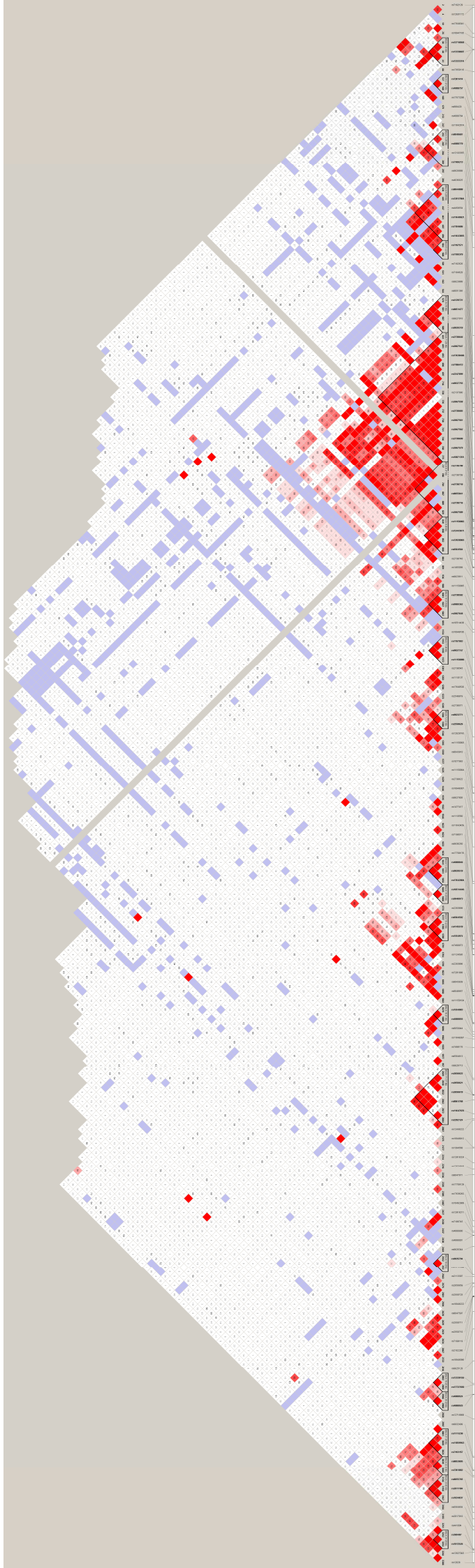

Supplement: Additional file 2 — Figure S2: LD map of WWOX gene in the CEU HapMap reference panel [file 1471-2350-11-148-S2.PDF]

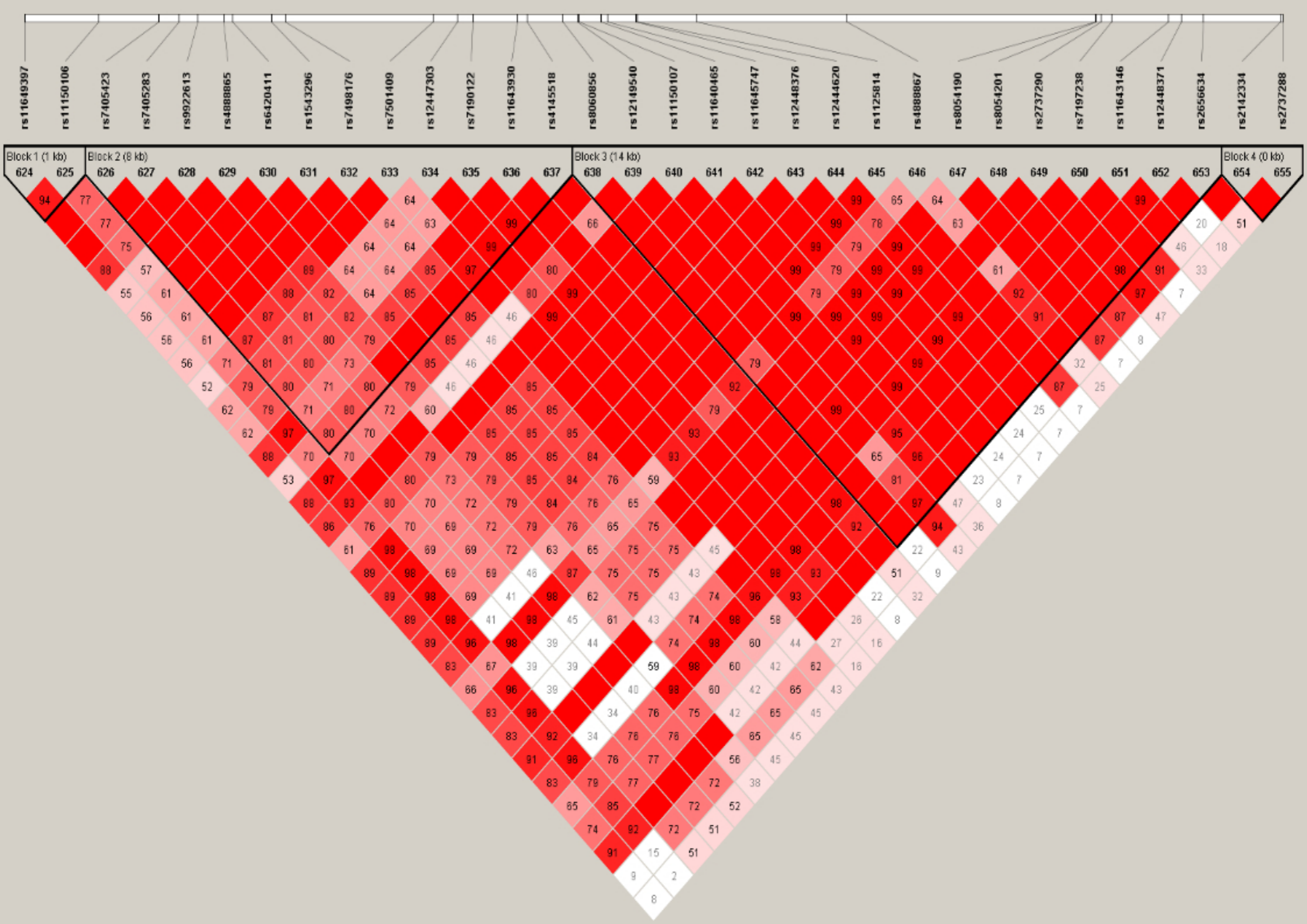

Supplement: Additional file 5 — Figure S4: LD map at WWOX region 2 (Chr.16: 77,433.428-77,441.867 bp) [file 1471-2350-11-148-S5.PDF]

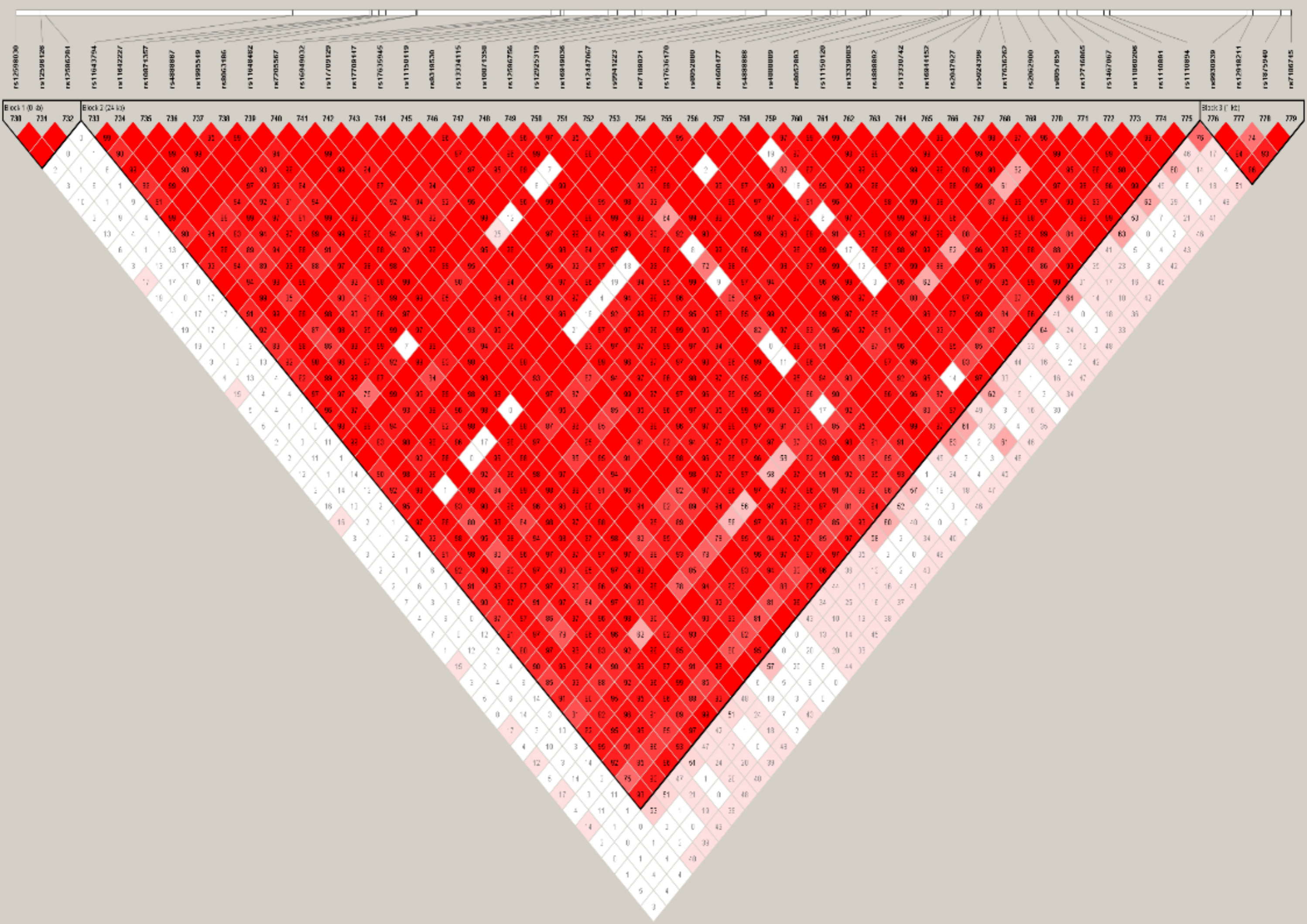

Supplement: Additional file 6 — Figure S5: LD map at WWOX region 3 (Chr.16: 77,538.855-77,563.088 bp) [file 1471-2350-11-148-S6.PDF]

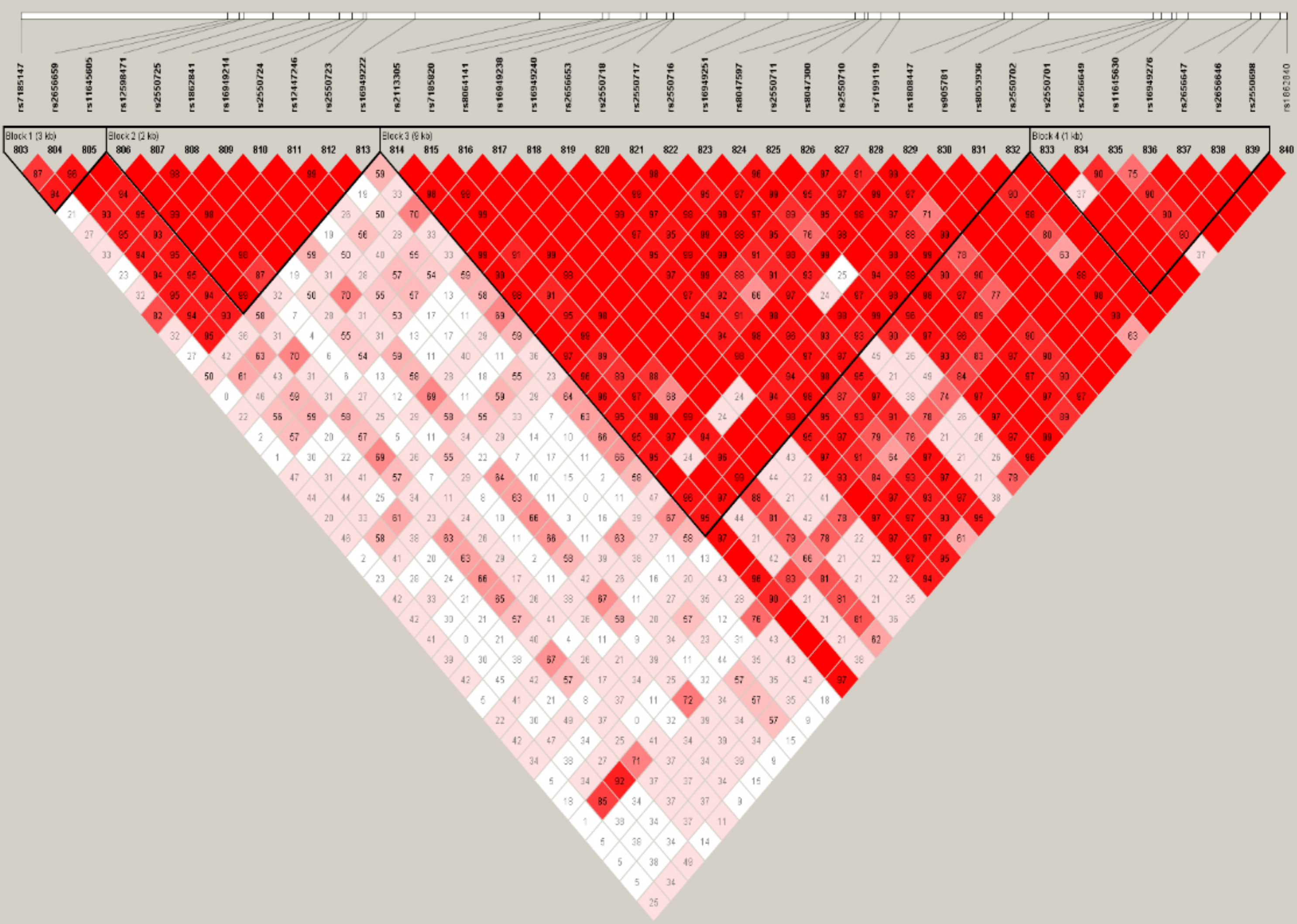

Supplement: Additional file 7 — Figure S6: LD map at WWOX region 4 (Chr.16: 77,605.536-77,620.657 bp) [file 1471-2350-11-148-S7.PDF]
