# Supplementary material for: WWOX gene is associated with HDL cholesterol and triglyceride levels
Source: BMC Med Genet. 2010 Oct 14;11:148. doi: 10.1186/1471-2350-11-148 (PMC2967537; doi:10.1186/1471-2350-11-148)
Supplement: Additional file 3 — Table S1: Quality metrics for imputed SNPs [file 1471-2350-11-148-S3.DOC]

| **SNP** | **BP** | **A1** | **A2** | **MAF** | **Quality** | **Rsq** |
| --- | --- | --- | --- | --- | --- | --- |
| rs10220974 | 76689775 | T | C | 0.15 | 0.9704 | 0.9044 |
| rs4887935 | 76691956 | C | T | 0.39 | 0.9819 | 0.9697 |
| rs8045450 | 76698765 | C | A | 0.34 | 0.9792 | 0.9567 |
| rs2287973 | 76700822 | G | T | 0.39 | 0.9466 | 0.8985 |
| rs2287972 | 76701108 | C | T | 0.49 | 0.9253 | 0.8702 |
| rs16947127 | 76709149 | C | T | 0.14 | 0.9416 | 0.8249 |
| rs16947129 | 76709247 | C | T | 0.15 | 0.9428 | 0.8301 |
| rs12917833 | 76710102 | G | C | 0.16 | 0.9495 | 0.8504 |
| rs10492874 | 76710132 | G | C | 0.47 | 0.8994 | 0.8367 |
| rs11645006 | 76710658 | G | A | 0.33 | 0.9330 | 0.8756 |
| rs2042356 | 76715856 | T | C | 0.44 | 0.9159 | 0.8541 |
| rs1079569 | 76716080 | G | A | 0.35 | 0.9191 | 0.8521 |
| rs12920698 | 76716255 | C | G | 0.17 | 0.9732 | 0.9209 |
| rs1076514 | 76716533 | A | G | 0.33 | 0.9573 | 0.9158 |
| rs8057015 | 76717963 | C | A | 0.42 | 0.9353 | 0.8920 |
| rs9319518 | 76718209 | G | C | 0.26 | 0.9531 | 0.8921 |
| rs12931246 | 76718455 | C | G | 0.17 | 0.9750 | 0.9264 |
| rs9319519 | 76720755 | A | G | 0.37 | 0.8730 | 0.7807 |
| rs11644207 | 76721736 | G | T | 0.22 | 0.9448 | 0.8699 |
| rs13334300 | 76721852 | C | A | 0.22 | 0.8745 | 0.7121 |
| rs7203218 | 76722306 | A | G | 0.20 | 0.8889 | 0.7430 |
| rs12716849 | 76725191 | G | A | 0.45 | 0.9714 | 0.9536 |
| rs12926275 | 76725923 | T | G | 0.16 | 0.9472 | 0.8342 |
| rs12928563 | 76726351 | T | A | 0.16 | 0.9728 | 0.9214 |
| rs9929762 | 76727176 | G | A | 0.39 | 0.9478 | 0.9137 |
| rs1074963 | 76727661 | C | G | 0.42 | 0.9828 | 0.9652 |
| rs1074964 | 76727759 | C | T | 0.42 | 0.9759 | 0.9512 |
| rs9922536 | 76728567 | T | C | 0.39 | 0.9405 | 0.8994 |
| rs9933156 | 76728809 | G | A | 0.39 | 0.9396 | 0.8978 |
| rs9937449 | 76728877 | C | T | 0.39 | 0.9341 | 0.8872 |
| rs16947173 | 76729096 | G | A | 0.23 | 0.9569 | 0.8919 |
| rs4888748 | 76730655 | A | G | 0.48 | 0.9258 | 0.8661 |
| rs8062483 | 76731553 | A | G | 0.42 | 0.9394 | 0.8825 |
| rs4888750 | 76735216 | C | G | 0.34 | 0.9282 | 0.8551 |
| rs3764299 | 76738266 | C | T | 0.17 | 0.9455 | 0.8494 |
| rs3764298 | 76738301 | A | G | 0.17 | 0.9306 | 0.8105 |
| rs3764297 | 76738442 | G | T | 0.17 | 0.9430 | 0.8434 |
| rs3764295 | 76738609 | G | T | 0.17 | 0.9427 | 0.8426 |
| rs17650073 | 76738796 | G | C | 0.18 | 0.8406 | 0.5718 |
| rs12935345 | 76739133 | C | T | 0.17 | 0.9411 | 0.8388 |
| rs11648121 | 76739487 | A | G | 0.17 | 0.9382 | 0.8324 |
| rs11648242 | 76739729 | C | G | 0.17 | 0.9368 | 0.8296 |
| rs11644853 | 76740383 | G | A | 0.17 | 0.9365 | 0.8290 |
| rs9924381 | 76741701 | T | C | 0.42 | 0.8472 | 0.7298 |
| rs7194147 | 76742625 | G | A | 0.41 | 0.8682 | 0.7474 |
| rs7359490 | 76742855 | T | C | 0.41 | 0.8656 | 0.7426 |
| rs1079573 | 76744555 | A | G | 0.44 | 0.8256 | 0.6841 |
| rs1079572 | 76744639 | G | A | 0.44 | 0.8249 | 0.6828 |
| rs1541690 | 76744651 | T | A | 0.20 | 0.8255 | 0.5902 |
| rs11860867 | 76745239 | T | A | 0.24 | 0.7597 | 0.4969 |
| rs12716850 | 76745404 | A | G | 0.47 | 0.7909 | 0.6303 |
| rs8056446 | 76745997 | G | A | 0.47 | 0.8043 | 0.6555 |
| rs12716852 | 76746239 | G | A | 0.47 | 0.8034 | 0.6539 |
| rs12716853 | 76746301 | A | G | 0.47 | 0.7992 | 0.6461 |
| rs8059793 | 76746799 | A | C | 0.40 | 0.8090 | 0.6471 |
| rs7206273 | 76747735 | G | A | 0.20 | 0.8157 | 0.5642 |
| rs9927362 | 76748253 | G | T | 0.19 | 0.8320 | 0.5537 |
| rs10871347 | 76748607 | C | A | 0.20 | 0.8113 | 0.5527 |
| rs1922618 | 76749271 | T | G | 0.20 | 0.8109 | 0.5518 |
| rs11645786 | 76750975 | T | A | 0.18 | 0.9480 | 0.8497 |
| rs12932880 | 76753394 | G | A | 0.25 | 0.7425 | 0.4621 |
| rs719743 | 76754788 | C | A | 0.25 | 0.7628 | 0.5200 |
| rs12923935 | 76754985 | A | C | 0.11 | 0.8985 | 0.6092 |
| rs2303190 | 76755737 | A | G | 0.17 | 0.9484 | 0.8483 |
| rs2113163 | 76756034 | A | G | 0.11 | 0.9018 | 0.6156 |
| rs4887940 | 76757590 | T | C | 0.14 | 0.8916 | 0.6264 |
| rs12926108 | 76757600 | G | C | 0.11 | 0.8895 | 0.5763 |
| rs4888754 | 76757603 | G | A | 0.17 | 0.9610 | 0.8841 |
| rs4887941 | 76757779 | G | A | 0.17 | 0.9618 | 0.8865 |
| rs11150044 | 76758793 | A | C | 0.37 | 0.8001 | 0.6440 |
| rs11150045 | 76759031 | G | C | 0.24 | 0.8842 | 0.7452 |
| rs11150046 | 76759162 | T | C | 0.17 | 0.9160 | 0.7451 |
| rs11150047 | 76759254 | A | C | 0.24 | 0.8855 | 0.7483 |
| rs2113160 | 76759454 | G | A | 0.24 | 0.7562 | 0.5101 |
| rs12923504 | 76760660 | G | C | 0.10 | 0.8833 | 0.5534 |
| rs8048466 | 76760681 | A | G | 0.25 | 0.7316 | 0.4737 |
| rs7206823 | 76762452 | T | A | 0.16 | 0.8176 | 0.5113 |
| rs7206730 | 76762965 | T | C | 0.18 | 0.9961 | 0.9881 |
| rs7189984 | 76763229 | C | T | 0.18 | 0.9872 | 0.9642 |
| rs8051687 | 76766177 | A | G | 0.17 | 0.8026 | 0.5014 |
| rs11643308 | 76766570 | G | T | 0.34 | 0.7658 | 0.6070 |
| rs7501067 | 76767164 | G | T | 0.19 | 0.9778 | 0.9493 |
| rs11862871 | 76771719 | G | C | 0.19 | 0.9067 | 0.7499 |
| rs2042354 | 76771795 | T | C | 0.19 | 0.9858 | 0.9624 |
| rs2042352 | 76772063 | C | G | 0.21 | 0.8383 | 0.6471 |
| rs16947244 | 76772276 | A | G | 0.19 | 0.9874 | 0.9660 |
| rs4887942 | 76772453 | G | A | 0.19 | 0.9888 | 0.9696 |
| rs4888755 | 76773002 | A | G | 0.19 | 0.9893 | 0.9710 |
| rs17573164 | 76774455 | G | A | 0.11 | 0.7682 | 0.3706 |
| rs4888759 | 76775582 | G | T | 0.19 | 0.9949 | 0.9874 |
| rs10521024 | 76779219 | G | A | 0.11 | 0.7660 | 0.3675 |
| rs10492873 | 76779471 | C | G | 0.11 | 0.9191 | 0.6915 |
| rs2345442 | 76780226 | G | T | 0.11 | 0.7652 | 0.3659 |
| rs4888761 | 76780490 | A | G | 0.33 | 0.7466 | 0.5791 |
| rs7192037 | 76790899 | A | G | 0.18 | 0.9362 | 0.8233 |
| rs7190387 | 76793549 | G | T | 0.22 | 0.8265 | 0.6275 |
| rs12924899 | 76793572 | C | T | 0.22 | 0.8251 | 0.6237 |
| rs12925637 | 76803021 | A | C | 0.19 | 0.8346 | 0.5753 |
| rs13336080 | 76807967 | T | G | 0.19 | 0.8281 | 0.5732 |
| rs17652533 | 76809117 | G | A | 0.21 | 0.8473 | 0.6350 |
| rs17574299 | 76810187 | G | A | 0.21 | 0.8493 | 0.6406 |
| rs9933348 | 76810835 | G | A | 0.21 | 0.8500 | 0.6426 |
| rs7188086 | 76815309 | A | G | 0.34 | 0.8189 | 0.6615 |
| rs4887946 | 76818022 | T | C | 0.21 | 0.8317 | 0.6045 |
| rs4887948 | 76818242 | G | C | 0.21 | 0.8281 | 0.5998 |
| rs2161635 | 76823824 | C | G | 0.31 | 0.8383 | 0.6842 |
| rs9935796 | 76824598 | G | A | 0.20 | 0.8456 | 0.6266 |
| rs1035530 | 76826414 | A | G | 0.33 | 0.7616 | 0.5522 |
| rs16947307 | 76827579 | T | C | 0.22 | 0.7995 | 0.5466 |
| rs7204887 | 76827833 | C | A | 0.22 | 0.7854 | 0.5247 |
| rs4888769 | 76837946 | T | C | 0.44 | 0.8281 | 0.7138 |
| rs8047321 | 76838148 | A | G | 0.16 | 0.9553 | 0.8646 |
| rs1079192 | 76841262 | A | G | 0.29 | 0.9563 | 0.9062 |
| rs1079191 | 76841349 | G | A | 0.29 | 0.9509 | 0.8933 |
| rs4377167 | 76842979 | T | C | 0.29 | 0.9776 | 0.9501 |
| rs7193919 | 76849354 | G | A | 0.47 | 0.8489 | 0.7675 |
| rs7196681 | 76853299 | C | A | 0.40 | 0.8556 | 0.7724 |
| rs8054537 | 76853337 | C | G | 0.40 | 0.9680 | 0.9372 |
| rs12923794 | 76859900 | C | T | 0.13 | 0.8372 | 0.5150 |
| rs4508428 | 76860767 | C | T | 0.15 | 0.8330 | 0.5354 |
| rs12929792 | 76861451 | G | T | 0.14 | 0.8280 | 0.4985 |
| rs6564520 | 76863720 | A | G | 0.41 | 0.9207 | 0.8634 |
| rs1124584 | 76866621 | T | C | 0.48 | 0.9907 | 0.9832 |
| rs4536491 | 76868101 | T | C | 0.39 | 0.8719 | 0.7706 |
| rs4270188 | 76868189 | C | A | 0.39 | 0.9052 | 0.8313 |
| rs4243149 | 76869274 | A | G | 0.39 | 0.8841 | 0.7909 |
| rs4243150 | 76869300 | G | A | 0.39 | 0.8763 | 0.7752 |
| rs11150053 | 76873767 | G | A | 0.21 | 0.7387 | 0.4450 |
| rs12927416 | 76874384 | C | G | 0.20 | 0.7551 | 0.4509 |
| rs12446017 | 76877135 | G | A | 0.36 | 0.6923 | 0.4307 |
| rs4427816 | 76878154 | C | T | 0.11 | 0.7413 | 0.3096 |
| rs4581713 | 76878167 | C | G | 0.11 | 0.7413 | 0.3082 |
| rs9928997 | 76879999 | C | A | 0.11 | 0.7518 | 0.3197 |
| rs11643583 | 76880159 | G | A | 0.11 | 0.7518 | 0.3195 |
| rs13333906 | 76880566 | T | A | 0.11 | 0.7518 | 0.3194 |
| rs9941255 | 76881031 | A | G | 0.11 | 0.7519 | 0.3190 |
| rs9934482 | 76881478 | A | T | 0.10 | 0.7562 | 0.3189 |
| rs4447442 | 76901946 | C | T | 0.12 | 0.7418 | 0.3210 |
| rs4887952 | 76904717 | A | G | 0.11 | 0.7849 | 0.3892 |
| rs9635572 | 76909405 | G | A | 0.10 | 0.7989 | 0.3635 |
| rs13339052 | 76935541 | G | C | 0.11 | 0.7737 | 0.4026 |
| rs4624193 | 76943796 | C | T | 0.45 | 0.6966 | 0.5170 |
| rs8064138 | 76947953 | C | T | 0.20 | 0.9971 | 0.9929 |
| rs12598990 | 76948879 | T | C | 0.11 | 0.9538 | 0.8161 |
| rs11648339 | 76950623 | C | T | 0.37 | 0.9121 | 0.8393 |
| rs4888786 | 76951541 | A | G | 0.37 | 0.9526 | 0.9182 |
| rs4243154 | 76954721 | C | T | 0.25 | 0.9234 | 0.8318 |
| rs7201630 | 76956767 | A | G | 0.13 | 0.8246 | 0.4793 |
| rs12918436 | 76960368 | C | T | 0.37 | 0.9762 | 0.9569 |
| rs12932569 | 76962019 | T | C | 0.34 | 0.9288 | 0.8856 |
| rs4412982 | 76962550 | A | T | 0.37 | 0.9610 | 0.9294 |
| rs11639904 | 76964024 | G | A | 0.37 | 0.9795 | 0.9666 |
| rs11150069 | 76964564 | A | C | 0.27 | 0.9797 | 0.9615 |
| rs13337989 | 76964954 | T | C | 0.27 | 0.9794 | 0.9608 |
| rs13338044 | 76965068 | T | C | 0.36 | 0.9744 | 0.9589 |
| rs11645844 | 76966473 | C | T | 0.37 | 0.9512 | 0.9156 |
| rs4888798 | 76968135 | C | T | 0.38 | 0.9726 | 0.9570 |
| rs4887961 | 76968429 | T | C | 0.22 | 0.9778 | 0.9556 |
| rs7189636 | 76969888 | A | G | 0.16 | 0.9841 | 0.9607 |
| rs4887962 | 76970217 | C | T | 0.38 | 0.9554 | 0.9209 |
| rs4888800 | 76970344 | C | T | 0.38 | 0.9603 | 0.9321 |
| rs4243156 | 76971397 | G | A | 0.17 | 0.9915 | 0.9773 |
| rs8058540 | 76971907 | G | C | 0.17 | 0.9523 | 0.8738 |
| rs7186569 | 76973049 | A | G | 0.17 | 0.9950 | 0.9870 |
| rs8055871 | 76975211 | G | A | 0.23 | 0.9938 | 0.9879 |
| rs8062393 | 76975312 | T | G | 0.16 | 0.9799 | 0.9376 |
| rs9928955 | 76977239 | G | C | 0.17 | 0.9948 | 0.9855 |
| rs12918952 | 76978276 | G | A | 0.38 | 0.9654 | 0.9396 |
| rs3897139 | 76986890 | G | A | 0.15 | 0.9167 | 0.7761 |
| rs4083383 | 76988134 | C | T | 0.17 | 0.8719 | 0.6588 |
| rs4609864 | 76988341 | G | A | 0.24 | 0.8573 | 0.7221 |
| rs11150075 | 76989993 | T | C | 0.15 | 0.7378 | 0.3781 |
| rs3764342 | 77024259 | C | A | 0.13 | 0.9910 | 0.9694 |
| rs7200731 | 77030931 | C | T | 0.16 | 0.9850 | 0.9603 |
| rs7185902 | 77033277 | G | C | 0.16 | 0.9903 | 0.9645 |
| rs7199023 | 77035049 | G | A | 0.16 | 0.9789 | 0.9266 |
| rs7197248 | 77047175 | C | G | 0.11 | 0.9617 | 0.8485 |
| rs2667657 | 77054377 | T | C | 0.13 | 0.9139 | 0.6569 |
| rs2941934 | 77056079 | A | G | 0.13 | 0.9267 | 0.7004 |
| rs2738664 | 77056971 | G | A | 0.21 | 0.9192 | 0.7695 |
| rs2738666 | 77057410 | T | A | 0.18 | 0.9263 | 0.7707 |
| rs2738673 | 77058753 | G | A | 0.25 | 0.9408 | 0.8432 |
| rs2667543 | 77059477 | C | T | 0.25 | 0.9750 | 0.9311 |
| rs9928087 | 77059778 | A | C | 0.14 | 0.9863 | 0.9413 |
| rs2667546 | 77060074 | G | C | 0.25 | 0.9896 | 0.9708 |
| rs2738674 | 77060238 | T | C | 0.25 | 0.9997 | 0.9992 |
| rs2738675 | 77060570 | A | G | 0.11 | 0.9997 | 0.9983 |
| rs9933282 | 77061049 | T | C | 0.14 | 0.9995 | 0.9984 |
| rs17638504 | 77061888 | G | C | 0.14 | 0.9985 | 0.9954 |
| rs2738679 | 77062499 | C | T | 0.25 | 0.9931 | 0.9829 |
| rs2738680 | 77062909 | G | A | 0.25 | 0.9908 | 0.9783 |
| rs2667552 | 77063407 | T | C | 0.21 | 0.9903 | 0.9766 |
| rs16947630 | 77063859 | G | T | 0.21 | 0.9929 | 0.9815 |
| rs9931884 | 77066163 | C | G | 0.15 | 0.9980 | 0.9938 |
| rs13336293 | 77066858 | T | G | 0.14 | 0.9974 | 0.9901 |
| rs2667555 | 77069538 | T | G | 0.17 | 0.9937 | 0.9805 |
| rs9931636 | 77069589 | C | A | 0.18 | 0.9879 | 0.9681 |
| rs8051225 | 77069814 | C | T | 0.21 | 0.9931 | 0.9819 |
| rs9936255 | 77070041 | G | T | 0.29 | 0.9538 | 0.8973 |
| rs2667556 | 77070054 | G | C | 0.17 | 0.9936 | 0.9792 |
| rs2667557 | 77070692 | T | C | 0.16 | 0.9823 | 0.9435 |
| rs4888804 | 77071161 | C | G | 0.20 | 0.9926 | 0.9780 |
| rs2738686 | 77072873 | C | G | 0.20 | 0.9985 | 0.9974 |
| rs2667568 | 77074710 | G | T | 0.19 | 0.9992 | 0.9978 |
| rs2667569 | 77074878 | G | A | 0.19 | 0.9991 | 0.9974 |
| rs2738691 | 77075102 | A | C | 0.19 | 0.9989 | 0.9968 |
| rs2738692 | 77075116 | A | T | 0.19 | 0.9307 | 0.8121 |
| rs2667572 | 77075383 | T | G | 0.19 | 0.9975 | 0.9929 |
| rs2738693 | 77075481 | C | T | 0.19 | 0.9971 | 0.9923 |
| rs2667573 | 77075655 | G | A | 0.19 | 0.9971 | 0.9921 |
| rs2738694 | 77076358 | A | G | 0.19 | 0.9963 | 0.9910 |
| rs2738695 | 77076383 | C | T | 0.19 | 0.9963 | 0.9910 |
| rs2738696 | 77076412 | A | G | 0.19 | 0.9964 | 0.9909 |
| rs16947701 | 77076491 | A | G | 0.12 | 0.9930 | 0.9749 |
| rs1540757 | 77077190 | C | T | 0.24 | 0.9091 | 0.7838 |
| rs2738697 | 77077546 | G | A | 0.21 | 0.9194 | 0.7920 |
| rs2254564 | 77077657 | C | T | 0.20 | 0.9990 | 0.9970 |
| rs2738706 | 77080114 | C | T | 0.20 | 0.9996 | 0.9988 |
| rs4888805 | 77081119 | A | T | 0.15 | 0.9960 | 0.9874 |
| rs2175473 | 77081266 | A | T | 0.15 | 0.9968 | 0.9898 |
| rs16947728 | 77081831 | A | G | 0.12 | 0.9993 | 0.9977 |
| rs12444091 | 77082641 | T | C | 0.12 | 0.9993 | 0.9977 |
| rs2738712 | 77082670 | C | G | 0.15 | 0.9996 | 0.9989 |
| rs12716855 | 77083313 | A | G | 0.15 | 0.9996 | 0.9988 |
| rs4887965 | 77083622 | G | A | 0.15 | 0.9996 | 0.9988 |
| rs3115956 | 77084533 | A | C | 0.15 | 0.9994 | 0.9981 |
| rs2738717 | 77084554 | C | G | 0.15 | 0.9993 | 0.9978 |
| rs2738719 | 77084661 | C | A | 0.15 | 0.9993 | 0.9976 |
| rs4888807 | 77085010 | A | G | 0.15 | 0.9977 | 0.9921 |
| rs4888808 | 77085037 | C | A | 0.23 | 0.9960 | 0.9901 |
| rs4888809 | 77085126 | C | T | 0.15 | 0.9991 | 0.9968 |
| rs2978629 | 77085559 | C | G | 0.16 | 0.9689 | 0.9034 |
| rs2859636 | 77085653 | G | C | 0.15 | 0.9990 | 0.9966 |
| rs2738722 | 77086120 | C | T | 0.15 | 0.9990 | 0.9966 |
| rs2738723 | 77086177 | T | G | 0.15 | 0.9721 | 0.9064 |
| rs5019441 | 77086367 | G | A | 0.15 | 0.9990 | 0.9966 |
| rs2738724 | 77087419 | C | T | 0.15 | 0.9991 | 0.9972 |
| rs2738726 | 77087562 | A | G | 0.15 | 0.9709 | 0.9026 |
| rs2667586 | 77087858 | A | C | 0.15 | 0.9993 | 0.9982 |
| rs2667587 | 77087915 | G | C | 0.15 | 0.9993 | 0.9984 |
| rs2738729 | 77088622 | A | G | 0.15 | 0.9961 | 0.9859 |
| rs3419 | 77089133 | G | T | 0.15 | 0.9942 | 0.9793 |
| rs2738736 | 77092324 | A | C | 0.17 | 0.9921 | 0.9749 |
| rs12931826 | 77092704 | T | A | 0.33 | 0.9320 | 0.8648 |
| rs2245201 | 77092871 | C | G | 0.17 | 0.9947 | 0.9822 |
| rs8062956 | 77094080 | C | G | 0.26 | 0.9938 | 0.9845 |
| rs11863365 | 77096077 | G | A | 0.22 | 0.9876 | 0.9748 |
| rs1877281 | 77096916 | A | G | 0.20 | 0.9902 | 0.9754 |
| rs8054925 | 77097883 | C | T | 0.20 | 0.9898 | 0.9743 |
| rs8055733 | 77097927 | A | C | 0.20 | 0.9896 | 0.9742 |
| rs1882957 | 77098833 | A | T | 0.17 | 0.9890 | 0.9704 |
| rs1882958 | 77098856 | G | T | 0.17 | 0.9892 | 0.9710 |
| rs17706509 | 77099224 | A | C | 0.16 | 0.9895 | 0.9721 |
| rs4257220 | 77101949 | A | G | 0.28 | 0.9862 | 0.9746 |
| rs11643490 | 77102458 | G | A | 0.26 | 0.9862 | 0.9719 |
| rs11643787 | 77102487 | C | T | 0.28 | 0.9877 | 0.9751 |
| rs1111683 | 77103726 | C | T | 0.29 | 0.9806 | 0.9567 |
| rs11150083 | 77103839 | G | C | 0.18 | 0.9449 | 0.8485 |
| rs2458031 | 77104310 | T | G | 0.31 | 0.9618 | 0.9207 |
| rs2459109 | 77104979 | G | C | 0.29 | 0.9565 | 0.9048 |
| rs1877284 | 77105156 | G | C | 0.34 | 0.9041 | 0.8177 |
| rs7195479 | 77105219 | A | G | 0.16 | 0.9699 | 0.9156 |
| rs1877285 | 77105299 | C | G | 0.28 | 0.9426 | 0.8734 |
| rs2738741 | 77105882 | A | G | 0.16 | 0.9631 | 0.8985 |
| rs8058087 | 77109997 | C | T | 0.10 | 0.8836 | 0.5393 |
| rs4319778 | 77110455 | A | G | 0.33 | 0.8213 | 0.6676 |
| rs16947913 | 77111719 | A | C | 0.12 | 0.9269 | 0.7686 |
| rs2941954 | 77114143 | A | G | 0.23 | 0.9000 | 0.8049 |
| rs9928512 | 77114500 | T | C | 0.12 | 0.9301 | 0.7790 |
| rs9930467 | 77115098 | A | G | 0.12 | 0.9353 | 0.7968 |
| rs2738744 | 77115740 | A | G | 0.22 | 0.9094 | 0.8205 |
| rs2941948 | 77117341 | C | G | 0.12 | 0.9611 | 0.8757 |
| rs2978632 | 77117381 | T | C | 0.30 | 0.8914 | 0.7857 |
| rs2738747 | 77118672 | G | C | 0.23 | 0.9209 | 0.8048 |
| rs11150084 | 77119321 | C | G | 0.32 | 0.8883 | 0.7744 |
| rs2859640 | 77121441 | A | T | 0.37 | 0.8682 | 0.7614 |
| rs8051498 | 77121942 | G | A | 0.14 | 0.9197 | 0.7312 |
| rs2859642 | 77124302 | T | C | 0.13 | 0.9521 | 0.8351 |
| rs2860236 | 77125177 | G | C | 0.21 | 0.9467 | 0.8608 |
| rs2738498 | 77125771 | T | C | 0.23 | 0.9464 | 0.8658 |
| rs2738499 | 77125917 | A | G | 0.21 | 0.9714 | 0.9346 |
| rs2667627 | 77125931 | C | T | 0.19 | 0.9730 | 0.9323 |
| rs2738501 | 77126571 | T | C | 0.23 | 0.9524 | 0.8810 |
| rs1106217 | 77129743 | C | T | 0.30 | 0.9940 | 0.9871 |
| rs1317575 | 77129756 | G | C | 0.30 | 0.9849 | 0.9705 |
| rs1105314 | 77130043 | A | G | 0.30 | 0.9828 | 0.9669 |
| rs1105313 | 77130110 | A | G | 0.10 | 0.9824 | 0.9341 |
| rs2859645 | 77130383 | C | G | 0.35 | 0.9121 | 0.8349 |
| rs2667632 | 77130805 | A | G | 0.10 | 0.9835 | 0.9375 |
| rs2667634 | 77131129 | C | G | 0.30 | 0.9733 | 0.9579 |
| rs12598440 | 77133608 | C | T | 0.42 | 0.8081 | 0.6855 |
| rs2667646 | 77135083 | C | T | 0.35 | 0.8861 | 0.8002 |
| rs12930620 | 77135779 | C | A | 0.35 | 0.8916 | 0.8108 |
| rs2978621 | 77136101 | C | T | 0.34 | 0.8787 | 0.7916 |
| rs11641880 | 77136401 | G | C | 0.13 | 0.9168 | 0.7167 |
| rs2667649 | 77138664 | C | T | 0.33 | 0.8674 | 0.7532 |
| rs2667650 | 77138707 | G | A | 0.33 | 0.9026 | 0.8197 |
| rs2738510 | 77141793 | G | A | 0.23 | 0.9564 | 0.8938 |
| rs2458029 | 77146033 | G | A | 0.20 | 0.9100 | 0.7658 |
| rs7203676 | 77148426 | T | C | 0.23 | 0.8837 | 0.7256 |
| rs9923592 | 77149054 | G | A | 0.12 | 0.9139 | 0.6586 |
| rs9933688 | 77149385 | G | C | 0.23 | 0.8577 | 0.6754 |
| rs2459108 | 77150377 | C | G | 0.36 | 0.7754 | 0.5988 |
| rs9925067 | 77153653 | G | C | 0.11 | 0.8745 | 0.5237 |
| rs12930179 | 77154699 | A | C | 0.11 | 0.8397 | 0.4511 |
| rs16948096 | 77154816 | C | T | 0.12 | 0.7835 | 0.3789 |
| rs2738521 | 77155150 | G | A | 0.12 | 0.7829 | 0.3774 |
| rs2673779 | 77155247 | T | C | 0.22 | 0.7523 | 0.4639 |
| rs10438629 | 77156653 | G | C | 0.15 | 0.8292 | 0.5013 |
| rs3853362 | 77162332 | G | T | 0.11 | 0.8648 | 0.5539 |
| rs2346007 | 77164753 | G | A | 0.25 | 0.7784 | 0.5539 |
| rs2943768 | 77170415 | C | A | 0.16 | 0.9295 | 0.7934 |
| rs2941935 | 77171262 | G | C | 0.15 | 0.9836 | 0.9425 |
| rs8056137 | 77171928 | G | C | 0.12 | 0.8494 | 0.5396 |
| rs9938843 | 77171952 | G | A | 0.15 | 0.9953 | 0.9862 |
| rs9921093 | 77172410 | G | T | 0.15 | 0.9972 | 0.9909 |
| rs9939269 | 77172473 | G | A | 0.15 | 0.9770 | 0.9201 |
| rs9928840 | 77172531 | T | C | 0.15 | 0.9947 | 0.9815 |
| rs9923563 | 77172961 | C | T | 0.22 | 0.8901 | 0.7386 |
| rs2247294 | 77174477 | C | G | 0.41 | 0.6990 | 0.5106 |
| rs4578663 | 77175432 | A | C | 0.17 | 0.8567 | 0.6175 |
| rs7196526 | 77177225 | G | C | 0.16 | 0.8444 | 0.5751 |
| rs16948137 | 77180189 | G | A | 0.11 | 0.8828 | 0.6002 |
| rs1828518 | 77192694 | C | T | 0.20 | 0.8941 | 0.7388 |
| rs2667542 | 77193196 | G | A | 0.35 | 0.8916 | 0.8048 |
| rs2287951 | 77197686 | C | G | 0.17 | 0.8497 | 0.6426 |
| rs1126185 | 77199241 | G | A | 0.26 | 0.8687 | 0.7395 |
| rs2738555 | 77203705 | A | G | 0.43 | 0.8497 | 0.7779 |
| rs2550581 | 77204286 | C | A | 0.43 | 0.8496 | 0.7779 |
| rs2550599 | 77212252 | C | G | 0.42 | 0.8781 | 0.8188 |
| rs2548876 | 77212320 | C | T | 0.17 | 0.8960 | 0.7096 |
| rs2738566 | 77213355 | A | C | 0.18 | 0.8691 | 0.6918 |
| rs2673775 | 77213512 | A | G | 0.43 | 0.8827 | 0.8253 |
| rs2738568 | 77213990 | T | C | 0.32 | 0.8972 | 0.8181 |
| rs2550604 | 77215086 | C | T | 0.42 | 0.8764 | 0.8112 |
| rs2738570 | 77215228 | G | T | 0.41 | 0.8535 | 0.7747 |
| rs2548861 | 77215894 | T | G | 0.37 | 0.8843 | 0.8094 |
| rs2550606 | 77217132 | C | G | 0.37 | 0.8961 | 0.8345 |
| rs2738573 | 77217831 | T | C | 0.26 | 0.9154 | 0.8358 |
| rs2738576 | 77218684 | A | C | 0.20 | 0.8873 | 0.7424 |
| rs2738577 | 77218875 | G | A | 0.36 | 0.8783 | 0.8087 |
| rs2550608 | 77219165 | C | G | 0.22 | 0.8574 | 0.7030 |
| rs2548866 | 77220759 | G | A | 0.17 | 0.8889 | 0.7302 |
| rs12598987 | 77222048 | C | G | 0.42 | 0.8840 | 0.8293 |
| rs4622523 | 77222243 | C | G | 0.27 | 0.8303 | 0.6871 |
| rs2550612 | 77222489 | A | G | 0.42 | 0.8848 | 0.8311 |
| rs10514439 | 77223246 | T | A | 0.43 | 0.8872 | 0.8356 |
| rs2550613 | 77223309 | T | G | 0.43 | 0.8888 | 0.8377 |
| rs1079323 | 77225347 | C | T | 0.13 | 0.8449 | 0.5442 |
| rs1110519 | 77225392 | G | A | 0.42 | 0.9091 | 0.8611 |
| rs12716856 | 77225709 | T | C | 0.11 | 0.8738 | 0.5846 |
| rs2550615 | 77225816 | G | C | 0.13 | 0.8644 | 0.5797 |
| rs7193983 | 77228393 | C | G | 0.25 | 0.9115 | 0.8037 |
| rs7196183 | 77228455 | G | A | 0.26 | 0.9224 | 0.8324 |
| rs2550619 | 77229995 | C | G | 0.42 | 0.9442 | 0.9050 |
| rs2550620 | 77230099 | A | C | 0.42 | 0.9482 | 0.9114 |
| rs6564571 | 77230157 | C | G | 0.33 | 0.9336 | 0.8752 |
| rs2432241 | 77231892 | C | T | 0.42 | 0.9799 | 0.9671 |
| rs2550621 | 77233032 | C | T | 0.42 | 0.9687 | 0.9475 |
| rs1107455 | 77234640 | G | A | 0.23 | 0.9705 | 0.9315 |
| rs2548873 | 77236321 | T | G | 0.42 | 0.9896 | 0.9836 |
| rs7189040 | 77236564 | T | G | 0.36 | 0.9697 | 0.9492 |
| rs7190546 | 77236632 | T | C | 0.27 | 0.9668 | 0.9377 |
| rs2881375 | 77236992 | C | T | 0.23 | 0.9765 | 0.9481 |
| rs2738589 | 77238261 | A | G | 0.42 | 0.9677 | 0.9475 |
| rs1124434 | 77238834 | C | T | 0.42 | 0.9606 | 0.9380 |
| rs1124433 | 77239012 | C | T | 0.42 | 0.9591 | 0.9361 |
| rs2738591 | 77239356 | C | T | 0.42 | 0.9545 | 0.9308 |
| rs8046010 | 77239458 | T | C | 0.23 | 0.9664 | 0.9345 |
| rs2548843 | 77239537 | C | A | 0.42 | 0.9464 | 0.9216 |
| rs9931114 | 77240965 | G | T | 0.36 | 0.9214 | 0.8749 |
| rs1574442 | 77241830 | A | G | 0.23 | 0.9685 | 0.9350 |
| rs1107984 | 77241875 | G | A | 0.23 | 0.9565 | 0.9026 |
| rs1125670 | 77248633 | T | C | 0.29 | 0.9701 | 0.9403 |
| rs1125671 | 77248787 | G | A | 0.44 | 0.9384 | 0.9134 |
| rs1109876 | 77253758 | C | T | 0.38 | 0.7611 | 0.5714 |
| rs11862140 | 77254936 | A | G | 0.12 | 0.9753 | 0.9051 |
| rs1364295 | 77258490 | T | C | 0.13 | 0.9474 | 0.7988 |
| rs16948273 | 77259908 | T | G | 0.11 | 0.9255 | 0.6967 |
| rs2550647 | 77260129 | C | T | 0.47 | 0.8818 | 0.8026 |
| rs2548836 | 77260157 | A | G | 0.40 | 0.8673 | 0.7688 |
| rs7194800 | 77263005 | T | C | 0.11 | 0.8763 | 0.5343 |
| rs8054120 | 77263482 | T | C | 0.11 | 0.8756 | 0.5259 |
| rs2194340 | 77264326 | C | T | 0.11 | 0.8613 | 0.4756 |
| rs6564575 | 77268743 | T | C | 0.17 | 0.8144 | 0.5432 |
| rs17708443 | 77270568 | G | C | 0.12 | 0.9098 | 0.6623 |
| rs2550655 | 77271027 | G | A | 0.32 | 0.8744 | 0.7611 |
| rs2738625 | 77274178 | G | A | 0.32 | 0.8700 | 0.7544 |
| rs9923705 | 77274362 | A | T | 0.42 | 0.8883 | 0.7903 |
| rs2738627 | 77275453 | G | A | 0.20 | 0.8234 | 0.6021 |
| rs12926298 | 77275528 | G | A | 0.44 | 0.7823 | 0.6161 |
| rs3751881 | 77276700 | C | T | 0.12 | 0.9121 | 0.6571 |
| rs4888826 | 77286563 | G | T | 0.50 | 0.8704 | 0.7645 |
| rs4887974 | 77288950 | A | C | 0.46 | 0.8401 | 0.7324 |
| rs12917793 | 77290412 | T | A | 0.49 | 0.8203 | 0.7069 |
| rs1110556 | 77291412 | G | A | 0.50 | 0.9181 | 0.8580 |
| rs1424159 | 77295094 | A | G | 0.50 | 0.9531 | 0.9275 |
| rs2042432 | 77298065 | T | A | 0.50 | 0.9364 | 0.8921 |
| rs7206203 | 77300240 | C | T | 0.50 | 0.9243 | 0.8753 |
| rs11645548 | 77311898 | C | T | 0.22 | 0.8706 | 0.7345 |
| rs4888831 | 77312188 | C | T | 0.24 | 0.8694 | 0.7099 |
| rs7187364 | 77312851 | C | A | 0.24 | 0.8728 | 0.7174 |
| rs11860176 | 77313635 | T | C | 0.20 | 0.8858 | 0.7147 |
| rs9925569 | 77313829 | A | G | 0.42 | 0.8718 | 0.8039 |
| rs7199640 | 77357683 | G | A | 0.31 | 0.8373 | 0.7049 |
| rs1469134 | 77358975 | G | A | 0.45 | 0.8474 | 0.7535 |
| rs7201295 | 77360990 | G | A | 0.42 | 0.9855 | 0.9736 |
| rs12716860 | 77361957 | G | C | 0.11 | 0.9779 | 0.9159 |
| rs13335618 | 77362357 | A | G | 0.12 | 0.8967 | 0.6433 |
| rs2161719 | 77362701 | T | C | 0.11 | 0.9787 | 0.9193 |
| rs1125678 | 77362743 | A | C | 0.14 | 0.9361 | 0.8034 |
| rs7198930 | 77364570 | G | C | 0.14 | 0.9359 | 0.8031 |
| rs7199334 | 77364735 | G | A | 0.14 | 0.9354 | 0.8017 |
| rs7206356 | 77364757 | T | G | 0.41 | 0.9317 | 0.8812 |
| rs7205435 | 77364835 | C | T | 0.43 | 0.9322 | 0.8834 |
| rs17709200 | 77365271 | G | A | 0.30 | 0.9945 | 0.9899 |
| rs9928973 | 77366013 | A | G | 0.30 | 0.9925 | 0.9852 |
| rs12445943 | 77366573 | A | G | 0.10 | 0.9530 | 0.7947 |
| rs9934620 | 77367817 | C | G | 0.28 | 0.9754 | 0.9424 |
| rs9925933 | 77368505 | G | A | 0.28 | 0.9181 | 0.8128 |
| rs9931387 | 77370149 | T | A | 0.28 | 0.9077 | 0.7886 |
| rs1477411 | 77371221 | G | A | 0.27 | 0.8510 | 0.6633 |
| rs1477412 | 77371460 | C | G | 0.27 | 0.8504 | 0.6621 |
| rs1477413 | 77371575 | T | C | 0.27 | 0.8454 | 0.6520 |
| rs1477414 | 77371616 | T | C | 0.27 | 0.8434 | 0.6478 |
| rs4035780 | 77371660 | G | A | 0.27 | 0.8399 | 0.6406 |
| rs4887986 | 77371886 | T | G | 0.25 | 0.8243 | 0.5990 |
| rs4887987 | 77371955 | A | G | 0.27 | 0.8377 | 0.6362 |
| rs4888847 | 77372130 | A | G | 0.27 | 0.8366 | 0.6339 |
| rs7185036 | 77383696 | A | G | 0.15 | 0.8131 | 0.4811 |
| rs2737302 | 77384321 | G | C | 0.25 | 0.7507 | 0.4704 |
| rs7198122 | 77384558 | C | T | 0.23 | 0.7976 | 0.5335 |
| rs9940926 | 77384816 | C | T | 0.15 | 0.8723 | 0.5877 |
| rs7197824 | 77384930 | G | C | 0.31 | 0.7661 | 0.5420 |
| rs12448575 | 77385034 | C | A | 0.16 | 0.8748 | 0.6068 |
| rs1110559 | 77385366 | A | G | 0.42 | 0.7097 | 0.4785 |
| rs9936415 | 77386046 | T | C | 0.31 | 0.7965 | 0.5827 |
| rs2737301 | 77386213 | C | T | 0.50 | 0.7705 | 0.5913 |
| rs2737297 | 77387476 | T | C | 0.42 | 0.7893 | 0.6332 |
| rs2737296 | 77387606 | G | C | 0.40 | 0.8284 | 0.6902 |
| rs9932188 | 77387916 | G | A | 0.15 | 0.8555 | 0.5445 |
| rs8063748 | 77388178 | G | C | 0.48 | 0.8381 | 0.7062 |
| rs7184456 | 77388632 | G | C | 0.16 | 0.8864 | 0.6594 |
| rs2737295 | 77389031 | T | C | 0.40 | 0.8439 | 0.7193 |
| rs1118736 | 77390649 | C | T | 0.24 | 0.8180 | 0.5955 |
| rs4888852 | 77390676 | T | A | 0.15 | 0.9041 | 0.6793 |
| rs1118735 | 77390825 | A | G | 0.41 | 0.8684 | 0.7650 |
| rs1118734 | 77390847 | T | A | 0.39 | 0.8564 | 0.7409 |
| rs2216730 | 77390953 | G | T | 0.15 | 0.8642 | 0.6068 |
| rs2737292 | 77391159 | A | G | 0.49 | 0.9067 | 0.8310 |
| rs11860793 | 77391933 | G | T | 0.35 | 0.8742 | 0.7589 |
| rs8050074 | 77392916 | G | C | 0.17 | 0.9254 | 0.7872 |
| rs9931801 | 77394531 | T | G | 0.16 | 0.9783 | 0.9206 |
| rs12443658 | 77395010 | T | C | 0.18 | 0.9524 | 0.8667 |
| rs3817671 | 77395244 | G | A | 0.18 | 0.9562 | 0.8790 |
| rs2293902 | 77395273 | T | C | 0.14 | 0.9277 | 0.7623 |
| rs2293901 | 77395439 | C | A | 0.49 | 0.9428 | 0.9009 |
| rs2293900 | 77395453 | T | G | 0.16 | 0.9897 | 0.9643 |
| rs2293897 | 77396251 | C | G | 0.16 | 0.9722 | 0.9199 |
| rs1124808 | 77397304 | C | G | 0.33 | 0.9795 | 0.9649 |
| rs1123882 | 77397336 | C | A | 0.15 | 0.9734 | 0.9186 |
| rs1554978 | 77397618 | G | T | 0.15 | 0.9737 | 0.9197 |
| rs1554977 | 77397829 | G | C | 0.15 | 0.9951 | 0.9836 |
| rs1554976 | 77397924 | T | A | 0.25 | 0.9948 | 0.9892 |
| rs6564594 | 77398246 | G | A | 0.25 | 0.9950 | 0.9894 |
| rs7501059 | 77399749 | T | A | 0.37 | 0.9832 | 0.9705 |
| rs6564595 | 77399849 | T | C | 0.15 | 0.9966 | 0.9879 |
| rs2293894 | 77401017 | G | C | 0.14 | 0.9919 | 0.9718 |
| rs9923322 | 77401370 | A | G | 0.16 | 0.9761 | 0.9272 |
| rs9936644 | 77401443 | G | T | 0.16 | 0.9752 | 0.9247 |
| rs3946180 | 77401485 | A | G | 0.16 | 0.9819 | 0.9464 |
| rs8052915 | 77402331 | C | T | 0.39 | 0.9755 | 0.9559 |
| rs12927430 | 77402643 | A | G | 0.34 | 0.9719 | 0.9507 |
| rs11643767 | 77403298 | C | G | 0.38 | 0.9576 | 0.9281 |
| rs12925461 | 77405456 | C | G | 0.30 | 0.9437 | 0.8904 |
| rs1530 | 77405595 | G | T | 0.40 | 0.9371 | 0.8925 |
| rs6564596 | 77406232 | A | C | 0.18 | 0.9803 | 0.9515 |
| rs7404312 | 77406876 | C | T | 0.49 | 0.9601 | 0.9391 |
| rs7205028 | 77409152 | C | G | 0.13 | 0.9890 | 0.9639 |
| rs6564597 | 77409425 | G | C | 0.13 | 0.9897 | 0.9657 |
| rs12447302 | 77410168 | A | C | 0.19 | 0.9261 | 0.8287 |
| rs12445110 | 77410183 | C | T | 0.19 | 0.9258 | 0.8272 |
| rs12928190 | 77411571 | G | A | 0.22 | 0.9805 | 0.9535 |
| rs7196220 | 77411729 | A | C | 0.30 | 0.9528 | 0.9141 |
| rs7199947 | 77412029 | G | A | 0.13 | 0.9934 | 0.9796 |
| rs12446194 | 77412372 | G | A | 0.15 | 0.9850 | 0.9636 |
| rs9940043 | 77412718 | C | G | 0.28 | 0.9538 | 0.9083 |
| rs12443743 | 77412830 | A | G | 0.15 | 0.9857 | 0.9642 |
| rs1126341 | 77413062 | A | G | 0.15 | 0.9860 | 0.9645 |
| rs6420407 | 77414081 | C | A | 0.34 | 0.9488 | 0.9091 |
| rs6564599 | 77414237 | G | C | 0.42 | 0.9533 | 0.9224 |
| rs7197765 | 77414462 | A | T | 0.24 | 0.9456 | 0.8770 |
| rs7191005 | 77414547 | G | A | 0.42 | 0.9531 | 0.9217 |
| rs2037961 | 77414800 | C | T | 0.42 | 0.9414 | 0.9021 |
| rs2037960 | 77415313 | G | A | 0.16 | 0.9764 | 0.9311 |
| rs7192635 | 77415860 | T | C | 0.16 | 0.9770 | 0.9323 |
| rs7193003 | 77416061 | C | A | 0.14 | 0.9575 | 0.8640 |
| rs1072247 | 77416144 | G | A | 0.25 | 0.9627 | 0.9168 |
| rs9936829 | 77416631 | A | C | 0.34 | 0.9800 | 0.9616 |
| rs9929243 | 77416735 | C | T | 0.34 | 0.9806 | 0.9626 |
| rs12930924 | 77417091 | A | G | 0.34 | 0.9829 | 0.9663 |
| rs12918472 | 77417161 | C | T | 0.37 | 0.9741 | 0.9495 |
| rs7206468 | 77417999 | T | A | 0.22 | 0.9576 | 0.8929 |
| rs7184196 | 77418249 | A | C | 0.12 | 0.9787 | 0.9214 |
| rs6564600 | 77418281 | T | C | 0.34 | 0.9869 | 0.9731 |
| rs7189479 | 77418436 | A | T | 0.34 | 0.9876 | 0.9744 |
| rs7184760 | 77418516 | A | C | 0.13 | 0.9630 | 0.8718 |
| rs7189824 | 77418631 | G | T | 0.16 | 0.9861 | 0.9569 |
| rs7499843 | 77419701 | A | T | 0.34 | 0.9963 | 0.9920 |
| rs6564603 | 77419863 | G | A | 0.36 | 0.9619 | 0.9267 |
| rs11150105 | 77419998 | T | C | 0.34 | 0.9958 | 0.9906 |
| rs1079635 | 77421116 | C | T | 0.34 | 0.9658 | 0.9244 |
| rs6564604 | 77421147 | A | G | 0.12 | 0.9675 | 0.8607 |
| rs9941131 | 77421314 | A | G | 0.46 | 0.9259 | 0.8539 |
| rs6420409 | 77421796 | T | C | 0.12 | 0.9450 | 0.7709 |
| rs8051656 | 77423251 | T | C | 0.27 | 0.8373 | 0.6605 |
| rs7404730 | 77423875 | C | G | 0.48 | 0.8129 | 0.6859 |
| rs8056057 | 77424158 | C | G | 0.32 | 0.8732 | 0.7792 |
| rs13338670 | 77424513 | G | C | 0.23 | 0.8918 | 0.7605 |
| rs1554981 | 77425610 | T | C | 0.24 | 0.9250 | 0.8403 |
| rs1126340 | 77425769 | A | G | 0.30 | 0.9685 | 0.9347 |
| rs12932650 | 77426617 | G | C | 0.34 | 0.8653 | 0.7729 |
| rs4888854 | 77426961 | A | C | 0.50 | 0.9742 | 0.9613 |
| rs1554979 | 77427254 | T | C | 0.24 | 0.9261 | 0.8426 |
| rs7185014 | 77427473 | T | C | 0.20 | 0.9629 | 0.9016 |
| rs12920972 | 77427741 | T | C | 0.24 | 0.9013 | 0.7800 |
| rs12923682 | 77427902 | C | G | 0.24 | 0.9173 | 0.8214 |
| rs13332126 | 77428556 | G | A | 0.24 | 0.9076 | 0.8001 |
| rs8060300 | 77428930 | C | T | 0.13 | 0.8803 | 0.6144 |
| rs6564607 | 77429337 | C | G | 0.43 | 0.8289 | 0.7344 |
| rs11150106 | 77432058 | C | T | 0.21 | 0.7883 | 0.5234 |
| rs7405423 | 77433428 | T | C | 0.12 | 0.9135 | 0.6995 |
| rs7405283 | 77433901 | G | A | 0.12 | 0.9143 | 0.7036 |
| rs9922613 | 77434319 | A | T | 0.12 | 0.9109 | 0.7064 |
| rs4888865 | 77434919 | C | A | 0.18 | 0.9093 | 0.7685 |
| rs6420411 | 77435119 | A | C | 0.24 | 0.9757 | 0.9383 |
| rs1543296 | 77436011 | A | G | 0.24 | 0.9938 | 0.9839 |
| rs7501409 | 77439717 | A | G | 0.23 | 0.9412 | 0.8587 |
| rs12447303 | 77440263 | C | G | 0.21 | 0.7660 | 0.4909 |
| rs7190122 | 77440619 | A | G | 0.21 | 0.7657 | 0.4900 |
| rs11643930 | 77441655 | C | T | 0.20 | 0.7635 | 0.4296 |
| rs4145518 | 77441867 | G | A | 0.15 | 0.8955 | 0.6899 |
| rs8060856 | 77442683 | C | A | 0.19 | 0.7587 | 0.4186 |
| rs12149540 | 77443030 | C | A | 0.13 | 0.7823 | 0.4017 |
| rs11150107 | 77443065 | G | T | 0.14 | 0.8832 | 0.6348 |
| rs11640465 | 77443564 | G | C | 0.19 | 0.7714 | 0.4311 |
| rs11645747 | 77443705 | G | A | 0.19 | 0.7710 | 0.4304 |
| rs12448376 | 77444363 | A | T | 0.19 | 0.7700 | 0.4279 |
| rs12444620 | 77444404 | A | G | 0.19 | 0.7688 | 0.4252 |
| rs1125814 | 77445747 | C | A | 0.13 | 0.7748 | 0.3767 |
| rs4888867 | 77449191 | T | G | 0.22 | 0.7529 | 0.4660 |
| rs8054190 | 77454882 | T | C | 0.13 | 0.7741 | 0.3746 |
| rs8054201 | 77454899 | T | C | 0.13 | 0.7736 | 0.3730 |
| rs2737290 | 77455007 | A | C | 0.12 | 0.7851 | 0.3866 |
| rs7197238 | 77455243 | G | A | 0.13 | 0.7726 | 0.3698 |
| rs11643146 | 77456545 | T | C | 0.14 | 0.8478 | 0.5418 |
| rs12448371 | 77456839 | C | T | 0.21 | 0.7329 | 0.4213 |
| rs2656634 | 77457346 | A | G | 0.11 | 0.7775 | 0.3688 |
| rs2142334 | 77459112 | C | G | 0.41 | 0.7756 | 0.6261 |
| rs2737288 | 77459158 | T | C | 0.32 | 0.7130 | 0.5137 |
| rs2178952 | 77459224 | G | A | 0.44 | 0.8180 | 0.7097 |
| rs7194588 | 77459825 | G | A | 0.13 | 0.9820 | 0.9313 |
| rs8051859 | 77460182 | G | A | 0.50 | 0.8287 | 0.7262 |
| rs1504885 | 77460769 | C | T | 0.40 | 0.8182 | 0.7171 |
| rs2737283 | 77461562 | A | G | 0.40 | 0.8166 | 0.7141 |
| rs2737282 | 77461719 | G | A | 0.40 | 0.8160 | 0.7130 |
| rs2252435 | 77463522 | A | T | 0.40 | 0.8153 | 0.7137 |
| rs17633136 | 77465881 | G | T | 0.17 | 0.7700 | 0.4568 |
| rs1079636 | 77467963 | C | G | 0.35 | 0.9025 | 0.8086 |
| rs1106616 | 77468342 | C | T | 0.34 | 0.9127 | 0.8270 |
| rs1079634 | 77468635 | G | T | 0.35 | 0.9278 | 0.8551 |
| rs2656630 | 77468827 | T | C | 0.35 | 0.9332 | 0.8653 |
| rs2656629 | 77469334 | T | A | 0.35 | 0.8834 | 0.7808 |
| rs11865743 | 77469494 | G | C | 0.12 | 0.8900 | 0.6050 |
| rs2656628 | 77469571 | A | C | 0.34 | 0.9601 | 0.9184 |
| rs2656626 | 77469615 | G | C | 0.34 | 0.9645 | 0.9274 |
| rs11862750 | 77469673 | C | T | 0.12 | 0.8928 | 0.6191 |
| rs2656624 | 77470231 | A | G | 0.35 | 0.9831 | 0.9651 |
| rs2656623 | 77470496 | G | A | 0.35 | 0.9925 | 0.9848 |
| rs2656620 | 77470888 | A | C | 0.36 | 0.9743 | 0.9618 |
| rs9923225 | 77471501 | A | C | 0.23 | 0.9160 | 0.8012 |
| rs16948720 | 77471812 | A | T | 0.13 | 0.9189 | 0.7139 |
| rs7404729 | 77473155 | A | G | 0.12 | 0.9007 | 0.6278 |
| rs7404742 | 77473227 | A | G | 0.12 | 0.9008 | 0.6285 |
| rs4435265 | 77473661 | G | A | 0.12 | 0.9012 | 0.6302 |
| rs17706982 | 77476484 | G | C | 0.35 | 0.9332 | 0.8924 |
| rs4887990 | 77478402 | G | A | 0.35 | 0.9334 | 0.8910 |
| rs4887991 | 77478564 | G | A | 0.36 | 0.9238 | 0.8753 |
| rs10492909 | 77480575 | G | A | 0.21 | 0.9025 | 0.7606 |
| rs11861122 | 77480823 | G | A | 0.22 | 0.9133 | 0.7939 |
| rs12926355 | 77481326 | G | T | 0.22 | 0.9140 | 0.7961 |
| rs11639743 | 77483496 | T | G | 0.42 | 0.9617 | 0.9416 |
| rs1124356 | 77486814 | C | A | 0.46 | 0.9661 | 0.9434 |
| rs2174403 | 77486959 | C | T | 0.16 | 0.9333 | 0.8121 |
| rs11150110 | 77489180 | T | C | 0.45 | 0.9901 | 0.9802 |
| rs8047431 | 77489323 | A | C | 0.37 | 0.9265 | 0.8715 |
| rs7192392 | 77489879 | A | G | 0.34 | 0.9108 | 0.8479 |
| rs9939235 | 77489915 | C | T | 0.39 | 0.8593 | 0.7705 |
| rs8052368 | 77490585 | C | G | 0.45 | 0.9437 | 0.8869 |
| rs2062894 | 77490988 | C | T | 0.46 | 0.9323 | 0.8648 |
| rs2062895 | 77491002 | G | A | 0.17 | 0.9053 | 0.7184 |
| rs11864605 | 77493231 | A | G | 0.14 | 0.8797 | 0.6023 |
| rs2047925 | 77493409 | C | G | 0.14 | 0.8759 | 0.5887 |
| rs16948787 | 77493607 | T | G | 0.12 | 0.9031 | 0.5858 |
| rs4888873 | 77494639 | C | T | 0.14 | 0.8731 | 0.5787 |
| rs6564621 | 77496319 | T | A | 0.43 | 0.7811 | 0.6042 |
| rs16948799 | 77497159 | G | A | 0.15 | 0.8667 | 0.5642 |
| rs4888874 | 77497189 | G | A | 0.34 | 0.8017 | 0.5951 |
| rs16948801 | 77497768 | G | C | 0.17 | 0.8606 | 0.5737 |
| rs16948804 | 77497905 | C | G | 0.35 | 0.7626 | 0.5323 |
| rs11861377 | 77498248 | C | T | 0.17 | 0.7696 | 0.3801 |
| rs8053244 | 77498288 | G | T | 0.32 | 0.7576 | 0.5213 |
| rs1014101 | 77502767 | C | T | 0.11 | 0.8346 | 0.3503 |
| rs7189505 | 77506867 | C | G | 0.15 | 0.7892 | 0.3951 |
| rs8055573 | 77508554 | T | C | 0.31 | 0.7550 | 0.5097 |
| rs2221434 | 77508627 | A | G | 0.19 | 0.9152 | 0.8021 |
| rs1125630 | 77509264 | C | G | 0.23 | 0.7025 | 0.4301 |
| rs7192452 | 77511517 | T | G | 0.21 | 0.8753 | 0.6895 |
| rs11645638 | 77517824 | T | C | 0.36 | 0.7175 | 0.5337 |
| rs12930134 | 77523606 | C | T | 0.17 | 0.9895 | 0.9614 |
| rs12928676 | 77523826 | C | G | 0.17 | 0.8909 | 0.6940 |
| rs1349 | 77527961 | G | C | 0.45 | 0.8762 | 0.7707 |
| rs2062897 | 77529503 | A | T | 0.46 | 0.8341 | 0.6912 |
| rs12598030 | 77530589 | A | G | 0.45 | 0.8097 | 0.6375 |
| rs12596126 | 77531330 | G | A | 0.46 | 0.7846 | 0.5964 |
| rs12596284 | 77531468 | C | G | 0.46 | 0.7826 | 0.5926 |
| rs11643794 | 77538855 | A | G | 0.38 | 0.7227 | 0.4931 |
| rs11642227 | 77541138 | T | A | 0.36 | 0.7968 | 0.6120 |
| rs10871357 | 77541221 | C | G | 0.42 | 0.7063 | 0.5119 |
| rs4888887 | 77541431 | T | G | 0.36 | 0.8123 | 0.6421 |
| rs1995549 | 77541637 | C | T | 0.40 | 0.7363 | 0.5544 |
| rs8063186 | 77542541 | T | C | 0.50 | 0.7328 | 0.5549 |
| rs11648482 | 77542574 | A | G | 0.36 | 0.8555 | 0.7265 |
| rs7205567 | 77546564 | C | G | 0.36 | 0.7705 | 0.6014 |
| rs16949032 | 77546850 | G | A | 0.11 | 0.9718 | 0.8691 |
| rs17709147 | 77548087 | A | T | 0.11 | 0.9383 | 0.7547 |
| rs17635945 | 77548287 | G | A | 0.11 | 0.9376 | 0.7519 |
| rs11150119 | 77548544 | A | G | 0.46 | 0.8133 | 0.7147 |
| rs9319530 | 77549079 | G | C | 0.50 | 0.7953 | 0.6805 |
| rs13334115 | 77549296 | G | C | 0.37 | 0.8441 | 0.7328 |
| rs10871358 | 77550043 | G | A | 0.50 | 0.7912 | 0.6730 |
| rs12596756 | 77550293 | T | A | 0.50 | 0.7904 | 0.6700 |
| rs12925319 | 77550925 | G | C | 0.37 | 0.8847 | 0.7904 |
| rs16949036 | 77552383 | G | A | 0.36 | 0.8635 | 0.7606 |
| rs12447067 | 77552975 | C | T | 0.24 | 0.7381 | 0.4372 |
| rs9941223 | 77553010 | C | T | 0.36 | 0.8579 | 0.7361 |
| rs7189021 | 77554351 | G | A | 0.10 | 0.8033 | 0.3890 |
| rs17636170 | 77554912 | G | C | 0.37 | 0.7881 | 0.6211 |
| rs8052880 | 77555114 | C | T | 0.38 | 0.8210 | 0.7013 |
| rs4600477 | 77555364 | G | A | 0.37 | 0.8241 | 0.6987 |
| rs4888888 | 77556002 | T | C | 0.12 | 0.7447 | 0.3078 |
| rs4888889 | 77556085 | G | C | 0.38 | 0.8235 | 0.7007 |
| rs8052893 | 77557050 | A | G | 0.48 | 0.8065 | 0.6831 |
| rs11150120 | 77558265 | C | G | 0.40 | 0.7898 | 0.6710 |
| rs13339083 | 77558424 | A | G | 0.48 | 0.8078 | 0.6851 |
| rs4888892 | 77558492 | G | C | 0.38 | 0.8104 | 0.6855 |
| rs13330742 | 77559187 | A | C | 0.37 | 0.9149 | 0.8453 |
| rs16944152 | 77559398 | C | T | 0.39 | 0.8102 | 0.6904 |
| rs2047927 | 77559442 | G | A | 0.50 | 0.7969 | 0.6794 |
| rs5024396 | 77559693 | A | G | 0.47 | 0.8143 | 0.6967 |
| rs2062900 | 77560484 | G | A | 0.37 | 0.7460 | 0.5625 |
| rs8057659 | 77561123 | A | G | 0.49 | 0.7206 | 0.5571 |
| rs12716865 | 77561730 | T | C | 0.35 | 0.7323 | 0.5256 |
| rs1467067 | 77561984 | C | A | 0.32 | 0.8123 | 0.6345 |
| rs11860206 | 77562298 | T | C | 0.36 | 0.7157 | 0.4934 |
| rs1110891 | 77563088 | G | A | 0.41 | 0.7171 | 0.5144 |
| rs1110894 | 77563252 | T | C | 0.33 | 0.7118 | 0.4720 |
| rs9930939 | 77567350 | T | C | 0.12 | 0.8805 | 0.5526 |
| rs1875940 | 77568367 | G | T | 0.49 | 0.8529 | 0.7533 |
| rs11150125 | 77568753 | C | G | 0.22 | 0.9633 | 0.9079 |
| rs11647122 | 77569510 | C | G | 0.13 | 0.9536 | 0.8202 |
| rs8063569 | 77570962 | C | G | 0.40 | 0.8639 | 0.7584 |
| rs924870 | 77571088 | T | C | 0.21 | 0.9291 | 0.8172 |
| rs8062872 | 77571223 | G | C | 0.45 | 0.8967 | 0.8267 |
| rs12919788 | 77571413 | G | C | 0.47 | 0.8345 | 0.7096 |
| rs12444526 | 77571963 | C | T | 0.50 | 0.7401 | 0.5704 |
| rs16949121 | 77572043 | T | A | 0.20 | 0.9022 | 0.7251 |
| rs2014980 | 77572260 | G | C | 0.40 | 0.7800 | 0.5917 |
| rs3751834 | 77573909 | G | A | 0.12 | 0.8422 | 0.4916 |
| rs2347080 | 77591484 | C | G | 0.26 | 0.7439 | 0.4512 |
| rs10492905 | 77593245 | G | A | 0.25 | 0.7199 | 0.4015 |
| rs905775 | 77595472 | G | C | 0.24 | 0.7969 | 0.5205 |
| rs2006902 | 77595532 | G | T | 0.25 | 0.7733 | 0.4927 |
| rs13338273 | 77596323 | G | A | 0.25 | 0.7756 | 0.4976 |
| rs13332888 | 77596461 | C | G | 0.25 | 0.8214 | 0.5823 |
| rs13332891 | 77596492 | A | G | 0.25 | 0.7774 | 0.5016 |
| rs9652678 | 77597067 | G | C | 0.15 | 0.8609 | 0.5522 |
| rs8056452 | 77600054 | A | G | 0.17 | 0.8762 | 0.6076 |
| rs1469135 | 77601455 | C | G | 0.18 | 0.8637 | 0.5943 |
| rs7184417 | 77601614 | T | C | 0.18 | 0.8578 | 0.5788 |
| rs7185147 | 77601679 | G | A | 0.18 | 0.8571 | 0.5768 |
| rs11645605 | 77604969 | T | C | 0.20 | 0.9004 | 0.7658 |
| rs12598471 | 77605036 | T | G | 0.15 | 0.9474 | 0.8301 |
| rs1862841 | 77606024 | T | A | 0.35 | 0.9142 | 0.8337 |
| rs16949214 | 77606478 | T | C | 0.15 | 0.9738 | 0.9143 |
| rs2550724 | 77606678 | T | G | 0.34 | 0.9072 | 0.8184 |
| rs12447246 | 77606842 | C | A | 0.30 | 0.8873 | 0.7973 |
| rs2550723 | 77606893 | C | G | 0.34 | 0.8993 | 0.8009 |
| rs2113305 | 77609498 | T | G | 0.28 | 0.7687 | 0.5137 |
| rs7185820 | 77610444 | G | A | 0.18 | 0.8128 | 0.5057 |
| rs8064141 | 77610545 | A | G | 0.44 | 0.8084 | 0.6597 |
| rs16949238 | 77611012 | A | G | 0.17 | 0.8459 | 0.5636 |
| rs16949240 | 77611111 | A | G | 0.17 | 0.8476 | 0.5685 |
| rs2656653 | 77611413 | T | C | 0.29 | 0.8166 | 0.6101 |
| rs2550718 | 77611507 | A | C | 0.29 | 0.8241 | 0.6277 |
| rs2550717 | 77611521 | A | G | 0.44 | 0.8238 | 0.6860 |
| rs2550716 | 77612599 | A | G | 0.29 | 0.8049 | 0.5837 |
| rs16949251 | 77613249 | G | A | 0.18 | 0.8550 | 0.6097 |
| rs8047300 | 77614450 | C | A | 0.28 | 0.9972 | 0.9944 |
| rs1808447 | 77616501 | T | C | 0.28 | 0.8945 | 0.7578 |
| rs905781 | 77616627 | G | C | 0.13 | 0.9533 | 0.8422 |
| rs8053936 | 77617158 | T | C | 0.43 | 0.8202 | 0.6645 |
| rs2550702 | 77618744 | C | A | 0.28 | 0.8715 | 0.7121 |
| rs2550701 | 77618863 | C | G | 0.44 | 0.8134 | 0.6659 |
| rs2656649 | 77619030 | G | A | 0.14 | 0.9793 | 0.9336 |
| rs11645630 | 77619102 | A | C | 0.45 | 0.7744 | 0.5927 |
| rs16949276 | 77619263 | A | G | 0.11 | 0.8815 | 0.5730 |
| rs2656646 | 77620360 | C | T | 0.14 | 0.9996 | 0.9984 |
| rs1862840 | 77620757 | C | G | 0.50 | 0.7908 | 0.6364 |
| rs1862839 | 77620818 | G | C | 0.37 | 0.8028 | 0.6303 |
| rs2656645 | 77621304 | T | C | 0.35 | 0.7693 | 0.5506 |
| rs12445245 | 77623837 | G | A | 0.13 | 0.9707 | 0.9007 |
| rs1120114 | 77625818 | T | C | 0.27 | 0.7412 | 0.4562 |
| rs2550694 | 77626238 | C | G | 0.15 | 0.9478 | 0.8512 |
| rs8055815 | 77626307 | G | A | 0.42 | 0.7021 | 0.4771 |
| rs2550692 | 77626862 | G | A | 0.37 | 0.6881 | 0.4468 |
| rs2550691 | 77627177 | T | A | 0.43 | 0.6673 | 0.4210 |
| rs2550690 | 77628479 | G | C | 0.41 | 0.6648 | 0.4095 |
| rs2550689 | 77630453 | G | T | 0.35 | 0.6742 | 0.4040 |
| rs2656614 | 77631530 | C | A | 0.12 | 0.9487 | 0.8439 |
| rs905780 | 77632003 | T | C | 0.12 | 0.9507 | 0.8445 |
| rs16949354 | 77632101 | G | A | 0.10 | 0.9237 | 0.7024 |
| rs2247465 | 77632812 | A | G | 0.16 | 0.8996 | 0.7584 |
| rs2656613 | 77633125 | G | C | 0.23 | 0.9232 | 0.8186 |
| rs2656612 | 77633983 | G | A | 0.18 | 0.9700 | 0.9202 |
| rs9940793 | 77634004 | T | A | 0.12 | 0.7957 | 0.3519 |
| rs2202422 | 77634339 | G | A | 0.18 | 0.9720 | 0.9242 |
| rs1110898 | 77634659 | G | A | 0.25 | 0.9216 | 0.8285 |
| rs2250443 | 77635041 | G | A | 0.20 | 0.9427 | 0.8628 |
| rs1110897 | 77636292 | C | T | 0.18 | 0.9792 | 0.9406 |
| rs13335371 | 77640271 | G | C | 0.13 | 0.8525 | 0.5278 |
| rs13335415 | 77640369 | G | C | 0.13 | 0.8522 | 0.5266 |
| rs9972825 | 77652446 | T | C | 0.13 | 0.8787 | 0.5745 |
| rs9940608 | 77653349 | T | C | 0.13 | 0.8801 | 0.5790 |
| rs17647978 | 77657555 | C | T | 0.12 | 0.9075 | 0.6109 |
| rs11150132 | 77658957 | A | T | 0.13 | 0.9491 | 0.7918 |
| rs12324967 | 77659211 | T | C | 0.13 | 0.8958 | 0.6058 |
| rs17726834 | 77659614 | T | C | 0.12 | 0.9348 | 0.7246 |
| rs9930132 | 77662123 | A | G | 0.14 | 0.7197 | 0.3816 |
| rs12149527 | 77668097 | T | C | 0.48 | 0.7386 | 0.5138 |
| rs8049292 | 77669238 | C | G | 0.47 | 0.7257 | 0.4843 |
| rs12926028 | 77669335 | T | C | 0.49 | 0.7326 | 0.4986 |
| rs12924981 | 77669364 | C | G | 0.49 | 0.7412 | 0.5142 |
| rs12716866 | 77669439 | G | C | 0.47 | 0.7596 | 0.5449 |
| rs12925253 | 77669515 | C | G | 0.47 | 0.7622 | 0.5503 |
| rs17648647 | 77669698 | C | A | 0.47 | 0.7624 | 0.5502 |
| rs12102852 | 77671120 | G | A | 0.49 | 0.8250 | 0.6692 |
| rs4888920 | 77673432 | G | A | 0.49 | 0.8434 | 0.7056 |
| rs17727594 | 77673793 | C | G | 0.47 | 0.8439 | 0.7095 |
| rs7199945 | 77674380 | A | G | 0.47 | 0.9551 | 0.9168 |
| rs9635581 | 77677101 | G | C | 0.11 | 0.7366 | 0.3011 |
| rs17727687 | 77681077 | T | G | 0.12 | 0.8743 | 0.5269 |
| rs11864213 | 77684697 | G | A | 0.41 | 0.7487 | 0.5363 |
| rs9933420 | 77686511 | C | G | 0.11 | 0.8510 | 0.4765 |
| rs1424112 | 77729457 | G | A | 0.14 | 0.9227 | 0.7642 |
| rs11647886 | 77730182 | T | G | 0.11 | 0.9509 | 0.7919 |
| rs9938556 | 77730351 | G | C | 0.23 | 0.9374 | 0.8474 |
| rs7200334 | 77734025 | T | C | 0.31 | 0.9404 | 0.8826 |
| rs12449066 | 77734794 | G | A | 0.12 | 0.9455 | 0.7852 |
| rs9925100 | 77735616 | C | T | 0.34 | 0.8903 | 0.8026 |
| rs7200529 | 77736034 | T | G | 0.34 | 0.9206 | 0.8531 |
| rs7202835 | 77736126 | T | A | 0.34 | 0.9126 | 0.8400 |
| rs8045284 | 77736905 | G | A | 0.36 | 0.9151 | 0.8418 |
| rs8044764 | 77736940 | G | C | 0.36 | 0.9160 | 0.8435 |
| rs8050239 | 77737173 | C | T | 0.36 | 0.9213 | 0.8544 |
| rs7190436 | 77737948 | C | G | 0.33 | 0.9441 | 0.9032 |
| rs7191404 | 77738424 | C | G | 0.32 | 0.9649 | 0.9354 |
| rs8056647 | 77739785 | A | G | 0.25 | 0.9863 | 0.9667 |
| rs12050992 | 77739884 | C | T | 0.28 | 0.9585 | 0.9086 |
| rs7188059 | 77740217 | C | T | 0.28 | 0.9582 | 0.9080 |
| rs7204022 | 77740270 | C | G | 0.29 | 0.9544 | 0.9052 |
| rs7184152 | 77740719 | G | A | 0.28 | 0.9793 | 0.9567 |
| rs9924268 | 77740853 | G | A | 0.30 | 0.9380 | 0.8794 |
| rs8046140 | 77740909 | A | G | 0.35 | 0.9386 | 0.8789 |
| rs7190400 | 77741635 | C | A | 0.32 | 0.9469 | 0.8900 |
| rs7190432 | 77741701 | T | A | 0.32 | 0.9490 | 0.8944 |
| rs7194608 | 77741847 | G | C | 0.28 | 0.9905 | 0.9804 |
| rs8052122 | 77742005 | A | G | 0.31 | 0.9819 | 0.9598 |
| rs4309411 | 77742254 | G | C | 0.32 | 0.9975 | 0.9950 |
| rs4309413 | 77742322 | T | C | 0.32 | 0.9975 | 0.9952 |
| rs4381615 | 77742501 | C | T | 0.32 | 0.9822 | 0.9636 |
| rs4459555 | 77742697 | C | T | 0.32 | 0.9977 | 0.9960 |
| rs4622524 | 77742714 | C | T | 0.32 | 0.9984 | 0.9969 |
| rs4315347 | 77742768 | T | C | 0.32 | 0.9987 | 0.9974 |
| rs13339141 | 77743269 | G | C | 0.28 | 0.9906 | 0.9801 |
| rs12934227 | 77743453 | G | C | 0.32 | 0.9721 | 0.9460 |
| rs1117007 | 77743697 | C | T | 0.33 | 0.9764 | 0.9511 |
| rs8050187 | 77744387 | C | T | 0.32 | 0.9746 | 0.9459 |
| rs9922411 | 77744426 | C | G | 0.24 | 0.9644 | 0.9157 |
| rs6564651 | 77751639 | T | C | 0.48 | 0.7840 | 0.6272 |
| rs8046352 | 77751761 | G | C | 0.47 | 0.8272 | 0.7070 |
| rs8050958 | 77752025 | G | C | 0.48 | 0.9070 | 0.8325 |
| rs2347567 | 77753693 | C | A | 0.49 | 0.9166 | 0.8571 |
| rs12443788 | 77754099 | C | G | 0.49 | 0.9047 | 0.8384 |
| rs12443833 | 77754290 | C | T | 0.50 | 0.9141 | 0.8575 |
| rs4888931 | 77756305 | A | G | 0.49 | 0.9180 | 0.8649 |
| rs1110544 | 77756611 | T | C | 0.49 | 0.9069 | 0.8499 |
| rs1110543 | 77756873 | C | T | 0.50 | 0.8484 | 0.7475 |
| rs1110542 | 77756915 | T | A | 0.50 | 0.8447 | 0.7438 |
| rs9972790 | 77757069 | C | G | 0.49 | 0.8320 | 0.7262 |
| rs9972791 | 77757091 | C | G | 0.49 | 0.8392 | 0.7374 |
| rs8051054 | 77757409 | C | T | 0.50 | 0.8346 | 0.7318 |
| rs9972794 | 77757509 | A | G | 0.49 | 0.8265 | 0.7168 |
| rs12051388 | 77757788 | C | T | 0.49 | 0.8302 | 0.7299 |
| rs11150140 | 77757915 | A | G | 0.50 | 0.8235 | 0.7155 |
| rs11150141 | 77757979 | A | G | 0.49 | 0.8240 | 0.7163 |
| rs17656178 | 77758680 | A | G | 0.48 | 0.8417 | 0.7423 |
| rs7203399 | 77759167 | G | A | 0.47 | 0.8840 | 0.8197 |
| rs7205445 | 77759193 | A | G | 0.48 | 0.8754 | 0.8039 |
| rs8046276 | 77760150 | G | C | 0.48 | 0.8852 | 0.8157 |
| rs1116525 | 77760691 | C | T | 0.48 | 0.8965 | 0.8187 |
| rs4888932 | 77762356 | C | G | 0.49 | 0.9725 | 0.9524 |
| rs12920362 | 77762586 | A | T | 0.48 | 0.9227 | 0.8637 |
| rs6564652 | 77762867 | C | G | 0.45 | 0.8602 | 0.7687 |
| rs6564653 | 77764739 | C | G | 0.49 | 0.9932 | 0.9893 |
| rs1111415 | 77766640 | G | C | 0.49 | 0.9746 | 0.9552 |
| rs1812063 | 77766723 | A | T | 0.49 | 0.9924 | 0.9885 |
| rs2011394 | 77767268 | C | G | 0.49 | 0.9787 | 0.9647 |
| rs1110546 | 77767463 | C | G | 0.49 | 0.9785 | 0.9645 |
| rs2011200 | 77767927 | C | G | 0.50 | 0.9922 | 0.9890 |
| rs1963839 | 77768048 | C | T | 0.50 | 0.9860 | 0.9776 |
| rs12444578 | 77769120 | A | G | 0.49 | 0.9632 | 0.9392 |
| rs8049330 | 77769305 | A | C | 0.49 | 0.9719 | 0.9553 |
| rs8053844 | 77769396 | T | C | 0.49 | 0.9621 | 0.9393 |
| rs12716871 | 77770740 | C | G | 0.49 | 0.9569 | 0.9338 |
| rs12934392 | 77770836 | A | T | 0.49 | 0.9547 | 0.9319 |
| rs12935684 | 77771615 | A | G | 0.49 | 0.9523 | 0.9339 |
| rs11860626 | 77773005 | A | G | 0.34 | 0.8640 | 0.7684 |
| rs4888014 | 77773722 | A | G | 0.25 | 0.7714 | 0.5361 |
| rs1424106 | 77774294 | A | G | 0.47 | 0.7812 | 0.6520 |
| rs1424107 | 77774441 | C | G | 0.47 | 0.7811 | 0.6518 |
| rs16949819 | 77777084 | G | A | 0.27 | 0.7746 | 0.5627 |
| rs17730134 | 77777161 | A | G | 0.40 | 0.8392 | 0.7209 |
| rs1108663 | 77779393 | A | G | 0.40 | 0.8621 | 0.7483 |
| rs446684 | 77779788 | G | A | 0.38 | 0.7838 | 0.6382 |
| rs1110910 | 77780499 | G | C | 0.46 | 0.9857 | 0.9717 |
| rs436387 | 77782992 | T | C | 0.48 | 0.7294 | 0.5538 |
| rs386497 | 77783493 | T | C | 0.44 | 0.7756 | 0.6009 |
| rs375992 | 77784354 | G | A | 0.23 | 0.8626 | 0.6560 |
| rs7189876 | 77785069 | T | C | 0.20 | 0.8365 | 0.5714 |
| rs378819 | 77785184 | G | A | 0.23 | 0.8662 | 0.6640 |
| rs388512 | 77787235 | G | C | 0.43 | 0.8817 | 0.7812 |
| rs382888 | 77787324 | A | C | 0.36 | 0.8351 | 0.7004 |
| rs385160 | 77787383 | G | C | 0.44 | 0.8769 | 0.7690 |
| rs383673 | 77789947 | A | C | 0.41 | 0.8236 | 0.6828 |
| rs448302 | 77791896 | T | A | 0.43 | 0.8969 | 0.8067 |
| rs424074 | 77792088 | G | A | 0.39 | 0.8993 | 0.8151 |
| rs410233 | 77792188 | T | C | 0.38 | 0.8991 | 0.8142 |
| rs59344 | 77792446 | A | C | 0.39 | 0.9190 | 0.8529 |
| rs398255 | 77792535 | A | G | 0.49 | 0.9512 | 0.9148 |
| rs454120 | 77793305 | A | G | 0.43 | 0.9617 | 0.9283 |
| rs445862 | 77793946 | C | G | 0.42 | 0.9446 | 0.9068 |
| rs400497 | 77794068 | A | C | 0.43 | 0.9346 | 0.8934 |
| rs870 | 77795088 | G | A | 0.48 | 0.9965 | 0.9943 |
| rs2016545 | 77795260 | T | C | 0.19 | 0.9953 | 0.9866 |
| rs12935369 | 77795402 | T | C | 0.47 | 0.9667 | 0.9504 |
| rs7203866 | 77795511 | T | C | 0.24 | 0.9710 | 0.9272 |
| rs7197664 | 77795861 | T | C | 0.28 | 0.9706 | 0.9362 |
| rs442608 | 77796467 | T | C | 0.22 | 0.9037 | 0.7682 |
| rs417711 | 77796873 | A | G | 0.41 | 0.9706 | 0.9506 |
| rs17642520 | 77797327 | C | T | 0.42 | 0.9522 | 0.9194 |
| rs17796342 | 77797365 | T | C | 0.23 | 0.9527 | 0.8962 |
| rs409150 | 77797889 | G | A | 0.47 | 0.9639 | 0.9439 |
| rs384228 | 77797937 | C | A | 0.47 | 0.9637 | 0.9437 |
| rs420196 | 77798109 | C | G | 0.47 | 0.9630 | 0.9434 |
| rs403632 | 77799255 | G | T | 0.23 | 0.9515 | 0.8936 |
| rs421405 | 77799640 | T | A | 0.28 | 0.9447 | 0.8874 |
| rs407083 | 77799945 | T | C | 0.48 | 0.9633 | 0.9440 |
| rs368920 | 77800003 | C | G | 0.48 | 0.9633 | 0.9441 |
| rs450829 | 77800271 | A | G | 0.48 | 0.9636 | 0.9446 |
| rs413263 | 77801308 | G | T | 0.49 | 0.9198 | 0.8735 |
| rs12446313 | 77801523 | G | A | 0.11 | 0.9231 | 0.7165 |
| rs386776 | 77801677 | G | C | 0.35 | 0.9013 | 0.8312 |
| rs438254 | 77801798 | C | T | 0.48 | 0.9689 | 0.9516 |
| rs418017 | 77802646 | T | C | 0.37 | 0.9849 | 0.9729 |
| rs384216 | 77802966 | T | C | 0.47 | 0.9751 | 0.9611 |
| rs383362 | 77803321 | G | T | 0.47 | 0.9764 | 0.9632 |
| rs2288034 | 77803457 | C | G | 0.41 | 0.9897 | 0.9822 |
| rs2288033 | 77803462 | T | C | 0.41 | 0.9914 | 0.9856 |
| rs391870 | 77804036 | T | C | 0.43 | 0.9841 | 0.9761 |
| rs368299 | 77804510 | A | C | 0.37 | 0.9968 | 0.9948 |
| rs409183 | 77804959 | T | G | 0.37 | 0.9645 | 0.9337 |
| rs435617 | 77805091 | G | C | 0.41 | 0.9934 | 0.9890 |
| rs17642789 | 77806843 | G | A | 0.47 | 0.9811 | 0.9709 |

Table S1. Quality metrics for imputed SNPs.

Quality: The average posterior probability for the most likely genotype. Rsq (r^2^) : A better quality measured which estimates the squared correlation between imputed and true genotypes.
