# Supplementary material for: WWOX gene is associated with HDL cholesterol and triglyceride levels
Source: BMC Med Genet. 2010 Oct 14;11:148. doi: 10.1186/1471-2350-11-148 (PMC2967537; doi:10.1186/1471-2350-11-148)
Supplement: Additional file 4 — Figure S3: LD map at WWOX region 1 (Chr.16: 76,689,775-76,716,533 bp) [file 1471-2350-11-148-S4.PDF]

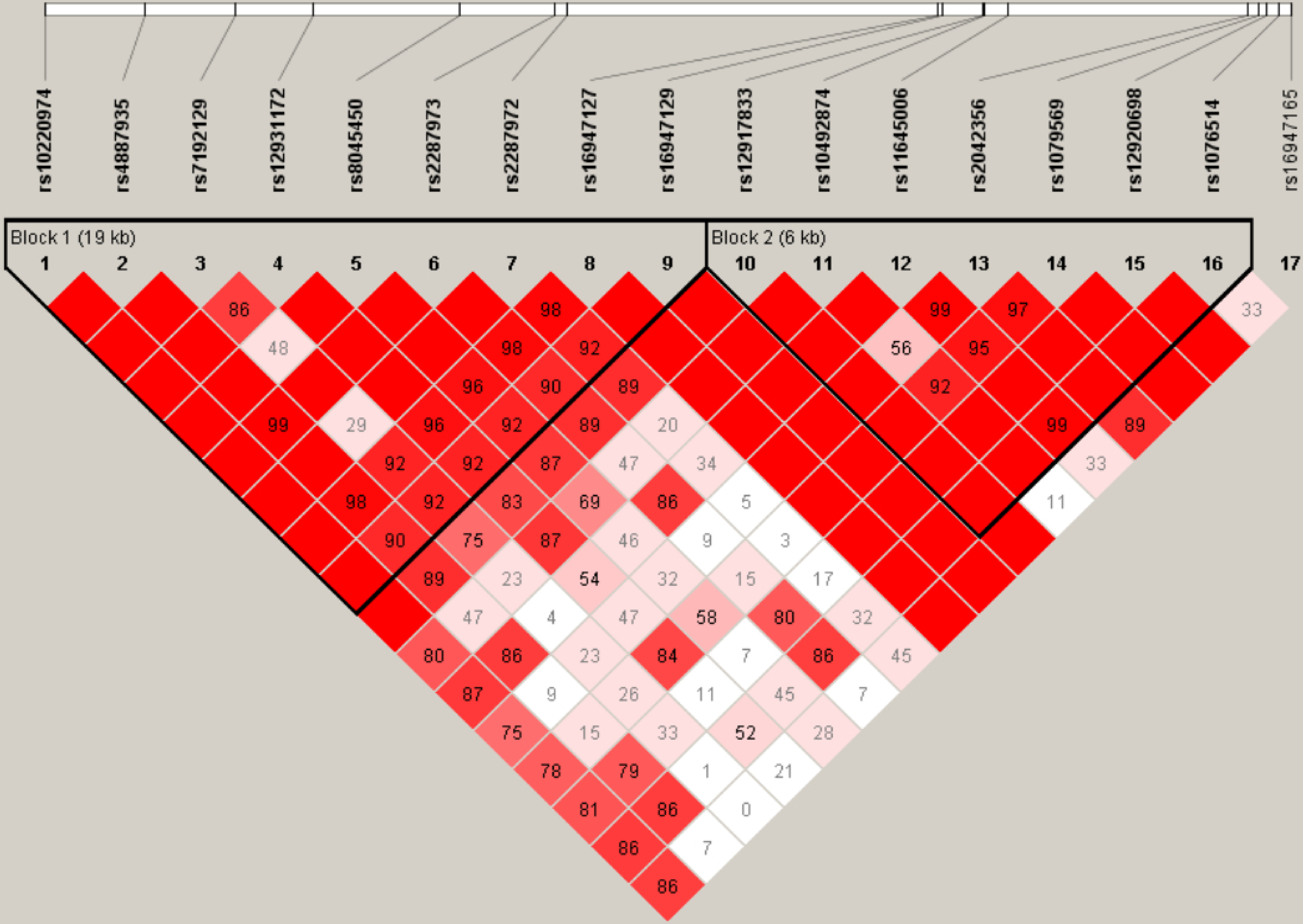
rs10220974
rs4887935
rs7192129
rs12931172
rs8045450
rs2287973
rs2287972
rs16947127
rs16947129
rs12917833
rs10492874
rs11645006
rs2042356
rs1079569
rs12920698
rs1076514
rs16947165
Block 1 (19 kb)
Block 2 (6 kb)
1 2 3 4 5 6 7 8 9 10 11 12 13 14 15 16 17
