# Supplementary material for: WWOX gene is associated with HDL cholesterol and triglyceride levels
Source: BMC Med Genet. 2010 Oct 14;11:148. doi: 10.1186/1471-2350-11-148 (PMC2967537; doi:10.1186/1471-2350-11-148)
Supplement: Additional file 9 — Table S3: Association analysis of the WWOX gene markers with triglycerides [file 1471-2350-11-148-S9.DOC]

| **SNP** | **BP** | **A1** | **A2** | **MAF** | **BETA** | **L95** | **U95** | **P** | **eP** | **STATUS** |
| --- | --- | --- | --- | --- | --- | --- | --- | --- | --- | --- |
| *rs10220974* | *76689775* | *T* | *C* | *0.15* | *8.35* | *-1.25* | *17.95* | *0.089* | *0.082* | *Imputed* |
| *rs4887935* | *76691956* | *C* | *T* | *0.39* | *9.44* | *2.36* | *16.52* | *0.009* | *0.010* | *Imputed* |
| *rs7192129* | *76693915* | *C* | *T* | *0.32* | *1.79* | *-5.60* | *9.19* | *0.635* | *0.800* | *Genotyped* |
| *rs12931172* | *76695591* | *C* | *G* | *0.30* | *-11.29* | *-18.75* | *-3.82* | *0.003* | *0.003* | *Genotyped* |
| *rs8045450* | *76698765* | *C* | *A* | *0.34* | *-12.67* | *-19.79* | *-5.54* | *0.001* | *0.000* | *Imputed* |
| *rs2287973* | *76700822* | *G* | *T* | *0.39* | *9.27* | *2.19* | *16.34* | *0.010* | *0.014* | *Imputed* |
| *rs2287972* | *76701108* | *C* | *T* | *0.49* | *-7.48* | *-14.38* | *-0.58* | *0.034* | *0.031* | *Imputed* |
| *rs16947127* | *76709149* | *C* | *T* | *0.14* | *-9.75* | *-19.46* | *-0.04* | *0.049* | *0.061* | *Imputed* |
| *rs16947129* | *76709247* | *C* | *T* | *0.15* | *-9.66* | *-19.14* | *-0.19* | *0.046* | *0.053* | *Imputed* |
| *rs12917833* | *76710102* | *G* | *C* | *0.16* | *-9.08* | *-18.38* | *0.23* | *0.056* | *0.071* | *Imputed* |
| *rs10492874* | *76710132* | *G* | *C* | *0.47* | *10.94* | *4.23* | *17.64* | *0.001* | *0.001* | *Imputed* |
| *rs11645006* | *76710658* | *G* | *A* | *0.33* | *-13.81* | *-21.16* | *-6.47* | *2.4E-04* | *1.8E-04* | *Imputed* |
| *rs2042356* | *76715856* | *T* | *C* | *0.44* | *7.97* | *1.05* | *14.88* | *0.024* | *0.026* | *Imputed* |
| *rs1079569* | *76716080* | *G* | *A* | *0.35* | *8.49* | *1.44* | *15.55* | *0.019* | *0.014* | *Imputed* |
| *rs12920698* | *76716255* | *C* | *G* | *0.17* | *-11.83* | *-20.87* | *-2.79* | *0.010* | *0.009* | *Imputed* |
| *rs1076514* | *76716533* | *A* | *G* | *0.33* | *-13.20* | *-20.59* | *-5.82* | *4.8E-04* | *3.7E-04* | *Imputed* |
| rs16947165 | 76716778 | G | A | 0.31 | -6.57 | -13.82 | 0.68 | 0.076 | 0.070 | Genotyped |
| rs8057015 | 76717963 | C | A | 0.42 | 5.72 | -1.19 | 12.63 | 0.105 | 0.102 | Imputed |
| rs9319518 | 76718209 | G | C | 0.26 | 5.82 | -2.03 | 13.66 | 0.147 | 0.156 | Imputed |
| rs12931246 | 76718455 | C | G | 0.17 | -11.83 | -20.87 | -2.79 | 0.010 | 0.009 | Imputed |
| rs9319519 | 76720755 | A | G | 0.37 | -4.08 | -11.34 | 3.18 | 0.271 | 0.200 | Imputed |
| rs11644207 | 76721736 | G | T | 0.22 | 2.89 | -5.28 | 11.06 | 0.489 | 0.383 | Imputed |
| rs13334300 | 76721852 | C | A | 0.22 | 2.60 | -5.65 | 10.84 | 0.537 | 0.425 | Imputed |
| rs7203218 | 76722306 | A | G | 0.20 | 5.37 | -3.19 | 13.92 | 0.219 | 0.198 | Imputed |
| rs12716848 | 76725159 | G | A | 0.42 | 4.74 | -2.19 | 11.67 | 0.180 | 0.166 | Genotyped |
| rs12716849 | 76725191 | G | A | 0.45 | 7.29 | 0.43 | 14.14 | 0.037 | 0.036 | Imputed |
| rs13338697 | 76725407 | G | A | 0.26 | 6.07 | -1.80 | 13.95 | 0.131 | 0.151 | Genotyped |
| rs13333314 | 76725431 | A | G | 0.26 | 6.07 | -1.80 | 13.95 | 0.131 | 0.151 | Genotyped |
| rs12926275 | 76725923 | T | G | 0.16 | -8.14 | -17.41 | 1.13 | 0.086 | 0.106 | Imputed |
| rs12928563 | 76726351 | T | A | 0.16 | -6.94 | -16.09 | 2.21 | 0.137 | 0.132 | Imputed |
| rs9929762 | 76727176 | G | A | 0.39 | -1.98 | -8.94 | 4.99 | 0.578 | 0.560 | Imputed |
| rs1074963 | 76727661 | C | G | 0.42 | 4.74 | -2.19 | 11.67 | 0.180 | 0.166 | Imputed |
| rs1074964 | 76727759 | C | T | 0.42 | 4.74 | -2.19 | 11.67 | 0.180 | 0.166 | Imputed |
| rs9922536 | 76728567 | T | C | 0.39 | -1.98 | -8.94 | 4.99 | 0.578 | 0.560 | Imputed |
| rs9933156 | 76728809 | G | A | 0.39 | -1.98 | -8.94 | 4.99 | 0.578 | 0.560 | Imputed |
| rs9937449 | 76728877 | C | T | 0.39 | -1.98 | -8.94 | 4.99 | 0.578 | 0.560 | Imputed |
| rs16947173 | 76729096 | G | A | 0.23 | 2.82 | -5.38 | 11.02 | 0.501 | 0.391 | Imputed |
| rs4888748 | 76730655 | A | G | 0.48 | 2.11 | -4.78 | 9.00 | 0.548 | 0.714 | Imputed |
| rs8062483 | 76731553 | A | G | 0.42 | 4.74 | -2.19 | 11.67 | 0.180 | 0.166 | Imputed |
| rs4888750 | 76735216 | C | G | 0.34 | 1.73 | -5.46 | 8.92 | 0.638 | 0.857 | Imputed |
| rs3764299 | 76738266 | C | T | 0.17 | -7.84 | -16.89 | 1.21 | 0.090 | 0.096 | Imputed |
| rs3764298 | 76738301 | A | G | 0.17 | -7.55 | -16.65 | 1.56 | 0.105 | 0.099 | Imputed |
| rs3764297 | 76738442 | G | T | 0.17 | -7.39 | -16.41 | 1.64 | 0.109 | 0.126 | Imputed |
| rs3764295 | 76738609 | G | T | 0.17 | -7.39 | -16.41 | 1.64 | 0.109 | 0.126 | Imputed |
| rs17650073 | 76738796 | G | C | 0.18 | 4.55 | -4.31 | 13.41 | 0.315 | 0.318 | Imputed |
| rs12935345 | 76739133 | C | T | 0.17 | -7.66 | -16.68 | 1.35 | 0.096 | 0.126 | Imputed |
| rs11648121 | 76739487 | A | G | 0.17 | -7.66 | -16.68 | 1.35 | 0.096 | 0.126 | Imputed |
| rs11648242 | 76739729 | C | G | 0.17 | -7.20 | -16.21 | 1.80 | 0.118 | 0.146 | Imputed |
| rs11644853 | 76740383 | G | A | 0.17 | -7.27 | -16.23 | 1.68 | 0.112 | 0.142 | Imputed |
| rs17650116 | 76741114 | A | G | 0.11 | -2.90 | -13.72 | 7.93 | 0.600 | 0.560 | Genotyped |
| rs8048266 | 76741315 | C | A | 0.38 | 2.95 | -4.13 | 10.03 | 0.414 | 0.419 | Genotyped |
| rs9924381 | 76741701 | T | C | 0.42 | -3.46 | -10.47 | 3.55 | 0.333 | 0.313 | Imputed |
| rs7194147 | 76742625 | G | A | 0.41 | 3.45 | -3.54 | 10.43 | 0.334 | 0.313 | Imputed |
| rs7359490 | 76742855 | T | C | 0.41 | 3.45 | -3.54 | 10.43 | 0.334 | 0.313 | Imputed |
| rs1079573 | 76744555 | A | G | 0.44 | 1.07 | -5.92 | 8.07 | 0.764 | 0.857 | Imputed |
| rs1079572 | 76744639 | G | A | 0.44 | 1.07 | -5.92 | 8.07 | 0.764 | 0.857 | Imputed |
| rs1541690 | 76744651 | T | A | 0.20 | 1.24 | -7.29 | 9.77 | 0.776 | 0.857 | Imputed |
| rs11860867 | 76745239 | T | A | 0.24 | 0.95 | -6.93 | 8.83 | 0.813 | 0.857 | Imputed |
| rs12716850 | 76745404 | A | G | 0.47 | 0.65 | -6.38 | 7.67 | 0.857 | 0.857 | Imputed |
| rs8056446 | 76745997 | G | A | 0.47 | 0.42 | -6.61 | 7.46 | 0.906 | 1.000 | Imputed |
| rs12716852 | 76746239 | G | A | 0.47 | 0.42 | -6.61 | 7.46 | 0.906 | 1.000 | Imputed |
| rs12716853 | 76746301 | A | G | 0.47 | 0.58 | -6.43 | 7.60 | 0.870 | 0.857 | Imputed |
| rs8059793 | 76746799 | A | C | 0.40 | 3.08 | -4.02 | 10.17 | 0.396 | 0.391 | Imputed |
| rs7206273 | 76747735 | G | A | 0.20 | 1.31 | -7.22 | 9.85 | 0.763 | 0.857 | Imputed |
| rs9927362 | 76748253 | G | T | 0.19 | 2.39 | -6.31 | 11.09 | 0.591 | 0.632 | Imputed |
| rs10871347 | 76748607 | C | A | 0.20 | 1.41 | -7.17 | 9.99 | 0.747 | 0.857 | Imputed |
| rs1922618 | 76749271 | T | G | 0.20 | 1.41 | -7.17 | 9.99 | 0.747 | 0.857 | Imputed |
| rs11645786 | 76750975 | T | A | 0.18 | -3.43 | -12.41 | 5.56 | 0.455 | 0.857 | Imputed |
| rs12932880 | 76753394 | G | A | 0.25 | 1.32 | -6.83 | 9.47 | 0.751 | 0.600 | Imputed |
| rs719743 | 76754788 | C | A | 0.25 | 0.07 | -7.80 | 7.93 | 0.987 | 1.000 | Imputed |
| rs12923935 | 76754985 | A | C | 0.11 | -8.52 | -19.34 | 2.30 | 0.123 | 0.092 | Imputed |
| rs2303190 | 76755737 | A | G | 0.17 | -3.92 | -12.91 | 5.07 | 0.393 | 0.857 | Imputed |
| rs2113163 | 76756034 | A | G | 0.11 | -8.56 | -19.49 | 2.38 | 0.126 | 0.102 | Imputed |
| rs4887940 | 76757590 | T | C | 0.14 | -3.43 | -13.38 | 6.52 | 0.499 | 0.500 | Imputed |
| rs12926108 | 76757600 | G | C | 0.11 | -8.56 | -19.49 | 2.38 | 0.126 | 0.102 | Imputed |
| rs4888754 | 76757603 | G | A | 0.17 | -3.82 | -12.80 | 5.16 | 0.404 | 0.857 | Imputed |
| rs4887941 | 76757779 | G | A | 0.17 | -3.82 | -12.80 | 5.16 | 0.404 | 0.857 | Imputed |
| rs11150044 | 76758793 | A | C | 0.37 | -7.50 | -14.63 | -0.36 | 0.040 | 0.044 | Imputed |
| rs11150045 | 76759031 | G | C | 0.24 | -2.85 | -10.84 | 5.14 | 0.485 | 0.460 | Imputed |
| rs11150046 | 76759162 | T | C | 0.17 | -3.99 | -13.02 | 5.04 | 0.386 | 0.388 | Imputed |
| rs11150047 | 76759254 | A | C | 0.24 | -2.83 | -10.82 | 5.16 | 0.488 | 0.500 | Imputed |
| rs2113160 | 76759454 | G | A | 0.24 | 0.31 | -7.61 | 8.23 | 0.939 | 1.000 | Imputed |
| rs12923504 | 76760660 | G | C | 0.10 | -8.07 | -19.19 | 3.04 | 0.155 | 0.120 | Imputed |
| rs8048466 | 76760681 | A | G | 0.25 | 0.58 | -7.38 | 8.53 | 0.887 | 1.000 | Imputed |
| rs7206823 | 76762452 | T | A | 0.16 | -1.58 | -11.00 | 7.85 | 0.743 | 0.857 | Imputed |
| rs7201414 | 76762567 | T | C | 0.18 | -3.56 | -12.49 | 5.37 | 0.435 | 0.857 | Genotyped |
| rs7206730 | 76762965 | T | C | 0.18 | -3.56 | -12.49 | 5.37 | 0.435 | 0.857 | Imputed |
| rs7189984 | 76763229 | C | T | 0.18 | -3.57 | -12.50 | 5.37 | 0.434 | 0.857 | Imputed |
| rs8051687 | 76766177 | A | G | 0.17 | -2.18 | -11.31 | 6.95 | 0.640 | 0.857 | Imputed |
| rs11643308 | 76766570 | G | T | 0.34 | 0.68 | -6.63 | 8.00 | 0.855 | 0.857 | Imputed |
| rs7501067 | 76767164 | G | T | 0.19 | -2.06 | -10.73 | 6.62 | 0.643 | 0.857 | Imputed |
| rs11862871 | 76771719 | G | C | 0.19 | -2.73 | -11.42 | 5.96 | 0.538 | 0.560 | Imputed |
| rs2042354 | 76771795 | T | C | 0.19 | -2.06 | -10.73 | 6.62 | 0.643 | 0.857 | Imputed |
| rs2042352 | 76772063 | C | G | 0.21 | -2.00 | -10.33 | 6.33 | 0.637 | 0.500 | Imputed |
| rs16947244 | 76772276 | A | G | 0.19 | -2.06 | -10.73 | 6.62 | 0.643 | 0.857 | Imputed |
| rs4887942 | 76772453 | G | A | 0.19 | -2.06 | -10.73 | 6.62 | 0.643 | 0.857 | Imputed |
| rs4888755 | 76773002 | A | G | 0.19 | -2.06 | -10.73 | 6.62 | 0.643 | 0.857 | Imputed |
| rs17573164 | 76774455 | G | A | 0.11 | 4.88 | -6.14 | 15.90 | 0.385 | 0.339 | Imputed |
| rs4888757 | 76774708 | G | A | 0.19 | -2.06 | -10.73 | 6.62 | 0.643 | 0.857 | Genotyped |
| rs17573298 | 76775517 | C | T | 0.20 | -4.69 | -13.14 | 3.76 | 0.277 | 0.346 | Genotyped |
| rs4888759 | 76775582 | G | T | 0.19 | -2.06 | -10.73 | 6.62 | 0.643 | 0.857 | Imputed |
| rs10521024 | 76779219 | G | A | 0.11 | 4.55 | -6.45 | 15.55 | 0.418 | 0.600 | Imputed |
| rs10492873 | 76779471 | C | G | 0.11 | 0.66 | -10.47 | 11.79 | 0.907 | 1.000 | Imputed |
| rs2345442 | 76780226 | G | T | 0.11 | 4.55 | -6.45 | 15.55 | 0.418 | 0.600 | Imputed |
| rs4888761 | 76780490 | A | G | 0.33 | 1.38 | -5.99 | 8.74 | 0.714 | 0.857 | Imputed |
| rs889420 | 76789480 | A | G | 0.14 | -1.45 | -10.94 | 8.05 | 0.765 | 1.000 | Genotyped |
| rs7192037 | 76790899 | A | G | 0.18 | -2.92 | -11.65 | 5.80 | 0.512 | 0.857 | Imputed |
| rs7190387 | 76793549 | G | T | 0.22 | -3.61 | -11.83 | 4.62 | 0.390 | 0.388 | Imputed |
| rs12924899 | 76793572 | C | T | 0.22 | -3.61 | -11.83 | 4.62 | 0.390 | 0.388 | Imputed |
| rs12925637 | 76803021 | A | C | 0.19 | -4.41 | -13.14 | 4.31 | 0.322 | 0.383 | Imputed |
| rs13336080 | 76807967 | T | G | 0.19 | -1.63 | -10.09 | 6.82 | 0.705 | 0.857 | Imputed |
| rs17652533 | 76809117 | G | A | 0.21 | -3.62 | -11.78 | 4.53 | 0.384 | 0.429 | Imputed |
| rs17574299 | 76810187 | G | A | 0.21 | -3.62 | -11.78 | 4.53 | 0.384 | 0.429 | Imputed |
| rs9933348 | 76810835 | G | A | 0.21 | -3.62 | -11.78 | 4.53 | 0.384 | 0.429 | Imputed |
| rs4888764 | 76811577 | T | G | 0.31 | 2.69 | -4.53 | 9.91 | 0.465 | 0.600 | Genotyped |
| rs7188086 | 76815309 | A | G | 0.34 | 4.10 | -3.20 | 11.40 | 0.271 | 0.284 | Imputed |
| rs4887946 | 76818022 | T | C | 0.21 | -3.63 | -11.84 | 4.57 | 0.386 | 0.457 | Imputed |
| rs4887948 | 76818242 | G | C | 0.21 | -3.28 | -11.44 | 4.87 | 0.430 | 0.560 | Imputed |
| rs34592201 | 76820588 | A | G | 0.14 | -1.13 | -10.74 | 8.47 | 0.817 | 1.000 | Genotyped |
| rs2161635 | 76823824 | C | G | 0.31 | 2.97 | -4.32 | 10.25 | 0.425 | 0.560 | Imputed |
| rs9935796 | 76824598 | G | A | 0.20 | -2.63 | -10.89 | 5.62 | 0.532 | 0.800 | Imputed |
| rs1035530 | 76826414 | A | G | 0.33 | 2.08 | -5.24 | 9.40 | 0.578 | 0.857 | Imputed |
| rs16947307 | 76827579 | T | C | 0.22 | -0.76 | -8.97 | 7.44 | 0.855 | 1.000 | Imputed |
| rs7204887 | 76827833 | C | A | 0.22 | -0.47 | -8.59 | 7.64 | 0.909 | 1.000 | Imputed |
| rs11642674 | 76830531 | T | C | 0.35 | -2.07 | -9.12 | 4.98 | 0.565 | 0.647 | Genotyped |
| rs8048681 | 76837677 | C | T | 0.19 | -2.10 | -10.50 | 6.31 | 0.625 | 0.800 | Genotyped |
| rs4888769 | 76837946 | T | C | 0.44 | -0.88 | -7.78 | 6.03 | 0.803 | 0.600 | Imputed |
| rs8047321 | 76838148 | A | G | 0.16 | 2.12 | -6.88 | 11.12 | 0.644 | 0.857 | Imputed |
| rs1079192 | 76841262 | A | G | 0.29 | 3.44 | -3.80 | 10.68 | 0.352 | 0.359 | Imputed |
| rs1079191 | 76841349 | G | A | 0.29 | 3.44 | -3.80 | 10.68 | 0.352 | 0.359 | Imputed |
| rs4377167 | 76842979 | T | C | 0.29 | 3.44 | -3.80 | 10.68 | 0.352 | 0.359 | Imputed |
| rs4888770 | 76843781 | G | A | 0.29 | 3.56 | -3.68 | 10.80 | 0.335 | 0.359 | Genotyped |
| rs7193919 | 76849354 | G | A | 0.47 | -1.64 | -8.55 | 5.27 | 0.643 | 0.750 | Imputed |
| rs7196681 | 76853299 | C | A | 0.40 | 6.35 | -0.42 | 13.11 | 0.066 | 0.053 | Imputed |
| rs8054537 | 76853337 | C | G | 0.40 | -1.94 | -8.85 | 4.98 | 0.583 | 0.471 | Imputed |
| rs7189213 | 76857692 | A | G | 0.40 | -1.94 | -8.85 | 4.98 | 0.583 | 0.471 | Genotyped |
| rs12923794 | 76859900 | C | T | 0.13 | 4.52 | -5.53 | 14.57 | 0.378 | 0.365 | Imputed |
| rs4508428 | 76860767 | C | T | 0.15 | 3.91 | -5.53 | 13.34 | 0.417 | 0.400 | Imputed |
| rs12929792 | 76861451 | G | T | 0.14 | 4.02 | -5.88 | 13.91 | 0.427 | 0.425 | Imputed |
| rs6564520 | 76863720 | A | G | 0.41 | -1.94 | -8.82 | 4.93 | 0.580 | 0.471 | Imputed |
| rs9926888 | 76866441 | T | C | 0.48 | -0.27 | -6.89 | 6.35 | 0.936 | 0.857 | Genotyped |
| rs1124584 | 76866621 | T | C | 0.48 | -0.27 | -6.89 | 6.35 | 0.936 | 0.857 | Imputed |
| rs4536491 | 76868101 | T | C | 0.39 | -3.27 | -10.09 | 3.56 | 0.348 | 0.309 | Imputed |
| rs4270188 | 76868189 | C | A | 0.39 | -3.31 | -10.13 | 3.50 | 0.341 | 0.292 | Imputed |
| rs4243149 | 76869274 | A | G | 0.39 | -3.27 | -10.09 | 3.56 | 0.348 | 0.309 | Imputed |
| rs4243150 | 76869300 | G | A | 0.39 | -3.27 | -10.09 | 3.56 | 0.348 | 0.309 | Imputed |
| rs11150053 | 76873767 | G | A | 0.21 | -1.73 | -10.15 | 6.69 | 0.687 | 0.800 | Imputed |
| rs12927416 | 76874384 | C | G | 0.20 | -0.44 | -8.98 | 8.10 | 0.920 | 1.000 | Imputed |
| rs12446017 | 76877135 | G | A | 0.36 | -1.64 | -8.77 | 5.49 | 0.652 | 0.600 | Imputed |
| rs4427816 | 76878154 | C | T | 0.11 | 1.90 | -8.83 | 12.63 | 0.728 | 0.750 | Imputed |
| rs4581713 | 76878167 | C | G | 0.11 | 2.61 | -8.15 | 13.38 | 0.635 | 0.565 | Imputed |
| rs9928997 | 76879999 | C | A | 0.11 | 3.22 | -7.76 | 14.19 | 0.566 | 0.591 | Imputed |
| rs11643583 | 76880159 | G | A | 0.11 | 3.22 | -7.76 | 14.19 | 0.566 | 0.591 | Imputed |
| rs13333906 | 76880566 | T | A | 0.11 | 3.22 | -7.76 | 14.19 | 0.566 | 0.591 | Imputed |
| rs9941255 | 76881031 | A | G | 0.11 | 3.56 | -7.43 | 14.55 | 0.526 | 0.539 | Imputed |
| rs9934482 | 76881478 | A | T | 0.10 | 5.22 | -5.91 | 16.35 | 0.358 | 0.359 | Imputed |
| rs4447442 | 76901946 | C | T | 0.12 | 2.27 | -8.24 | 12.78 | 0.673 | 0.857 | Imputed |
| rs4887952 | 76904717 | A | G | 0.11 | 1.71 | -9.16 | 12.59 | 0.758 | 0.857 | Imputed |
| rs9635572 | 76909405 | G | A | 0.10 | 6.64 | -4.75 | 18.03 | 0.254 | 0.250 | Imputed |
| rs13339052 | 76935541 | G | C | 0.11 | 4.69 | -6.00 | 15.38 | 0.390 | 0.328 | Imputed |
| rs4624193 | 76943796 | C | T | 0.45 | -3.35 | -10.31 | 3.61 | 0.346 | 0.318 | Imputed |
| rs8064138 | 76947953 | C | T | 0.20 | 2.31 | -6.06 | 10.67 | 0.589 | 0.565 | Imputed |
| rs8044888 | 76948718 | C | T | 0.20 | 2.31 | -6.06 | 10.67 | 0.589 | 0.565 | Genotyped |
| rs12598990 | 76948879 | T | C | 0.11 | 5.48 | -5.30 | 16.25 | 0.320 | 0.239 | Imputed |
| rs11648339 | 76950623 | C | T | 0.37 | -0.72 | -7.59 | 6.16 | 0.838 | 0.857 | Imputed |
| rs4888786 | 76951541 | A | G | 0.37 | -0.72 | -7.59 | 6.16 | 0.838 | 0.857 | Imputed |
| rs4243154 | 76954721 | C | T | 0.25 | -2.87 | -10.77 | 5.03 | 0.477 | 0.485 | Imputed |
| rs7201630 | 76956767 | A | G | 0.13 | -3.53 | -13.73 | 6.68 | 0.498 | 0.391 | Imputed |
| rs12917864 | 76959503 | T | G | 0.36 | -0.28 | -7.18 | 6.61 | 0.936 | 1.000 | Genotyped |
| rs12918436 | 76960368 | C | T | 0.37 | -0.31 | -7.18 | 6.56 | 0.930 | 1.000 | Imputed |
| rs12932569 | 76962019 | T | C | 0.34 | -0.24 | -7.27 | 6.80 | 0.948 | 1.000 | Imputed |
| rs4412982 | 76962550 | A | T | 0.37 | 0.03 | -6.80 | 6.86 | 0.992 | 1.000 | Imputed |
| rs11649021 | 76963690 | G | C | 0.16 | 1.70 | -7.36 | 10.76 | 0.713 | 0.750 | Genotyped |
| rs11639904 | 76964024 | G | A | 0.37 | 0.52 | -6.31 | 7.35 | 0.882 | 0.857 | Imputed |
| rs11150069 | 76964564 | A | C | 0.27 | -0.26 | -7.74 | 7.22 | 0.946 | 1.000 | Imputed |
| rs13337989 | 76964954 | T | C | 0.27 | -0.26 | -7.74 | 7.22 | 0.946 | 1.000 | Imputed |
| rs13338044 | 76965068 | T | C | 0.36 | 0.05 | -6.88 | 6.99 | 0.988 | 0.857 | Imputed |
| rs11645844 | 76966473 | C | T | 0.37 | -0.74 | -7.64 | 6.15 | 0.833 | 1.000 | Imputed |
| rs4888798 | 76968135 | C | T | 0.38 | -0.32 | -7.14 | 6.50 | 0.927 | 1.000 | Imputed |
| rs4887961 | 76968429 | T | C | 0.22 | 0.46 | -7.55 | 8.48 | 0.910 | 1.000 | Imputed |
| rs7189636 | 76969888 | A | G | 0.16 | -0.90 | -9.99 | 8.18 | 0.846 | 1.000 | Imputed |
| rs4887962 | 76970217 | C | T | 0.38 | -0.67 | -7.52 | 6.19 | 0.849 | 0.857 | Imputed |
| rs4888800 | 76970344 | C | T | 0.38 | -0.82 | -7.68 | 6.03 | 0.814 | 0.857 | Imputed |
| rs4243156 | 76971397 | G | A | 0.17 | 0.48 | -8.35 | 9.31 | 0.915 | 1.000 | Imputed |
| rs8058540 | 76971907 | G | C | 0.17 | -0.12 | -8.88 | 8.64 | 0.978 | 1.000 | Imputed |
| rs7186569 | 76973049 | A | G | 0.17 | 0.48 | -8.35 | 9.31 | 0.915 | 1.000 | Imputed |
| rs7184686 | 76973139 | G | A | 0.38 | -0.09 | -6.85 | 6.66 | 0.979 | 1.000 | Genotyped |
| rs9930967 | 76974815 | T | C | 0.16 | -0.36 | -9.41 | 8.69 | 0.938 | 1.000 | Genotyped |
| rs8055871 | 76975211 | G | A | 0.23 | 2.13 | -5.63 | 9.88 | 0.591 | 0.857 | Imputed |
| rs8062393 | 76975312 | T | G | 0.16 | -0.36 | -9.41 | 8.69 | 0.938 | 1.000 | Imputed |
| rs11643643 | 76977006 | C | G | 0.38 | -0.13 | -6.88 | 6.61 | 0.969 | 1.000 | Genotyped |
| rs9928955 | 76977239 | G | C | 0.17 | 0.33 | -8.49 | 9.16 | 0.941 | 1.000 | Imputed |
| rs11643855 | 76977459 | C | T | 0.23 | 1.64 | -6.11 | 9.39 | 0.679 | 0.647 | Genotyped |
| rs12918952 | 76978276 | G | A | 0.38 | -0.82 | -7.58 | 5.94 | 0.812 | 0.857 | Imputed |
| rs3897139 | 76986890 | G | A | 0.15 | -4.84 | -14.28 | 4.61 | 0.316 | 0.373 | Imputed |
| rs4083383 | 76988134 | C | T | 0.17 | -0.25 | -9.11 | 8.61 | 0.956 | 1.000 | Imputed |
| rs4609864 | 76988341 | G | A | 0.24 | -2.50 | -10.23 | 5.23 | 0.526 | 0.565 | Imputed |
| rs11150075 | 76989993 | T | C | 0.15 | 1.15 | -8.53 | 10.82 | 0.817 | 0.800 | Imputed |
| rs4129721 | 77016847 | C | A | 0.12 | -5.91 | -16.51 | 4.68 | 0.274 | 0.253 | Genotyped |
| rs3764342 | 77024259 | C | A | 0.13 | -7.84 | -17.88 | 2.21 | 0.127 | 0.118 | Imputed |
| rs8051477 | 77026670 | C | T | 0.13 | -7.28 | -17.31 | 2.75 | 0.155 | 0.135 | Genotyped |
| rs7200731 | 77030931 | C | T | 0.16 | -6.92 | -16.34 | 2.50 | 0.150 | 0.140 | Imputed |
| rs9929319 | 77031908 | C | G | 0.16 | -7.03 | -16.51 | 2.46 | 0.147 | 0.137 | Genotyped |
| rs7185902 | 77033277 | G | C | 0.16 | -7.03 | -16.51 | 2.46 | 0.147 | 0.137 | Imputed |
| rs7199023 | 77035049 | G | A | 0.16 | -7.03 | -16.51 | 2.46 | 0.147 | 0.137 | Imputed |
| rs7197248 | 77047175 | C | G | 0.11 | -7.49 | -18.37 | 3.39 | 0.177 | 0.200 | Imputed |
| rs2667657 | 77054377 | T | C | 0.13 | -1.63 | -11.96 | 8.70 | 0.757 | 1.000 | Imputed |
| rs2941934 | 77056079 | A | G | 0.13 | -1.69 | -12.00 | 8.62 | 0.748 | 1.000 | Imputed |
| rs2738664 | 77056971 | G | A | 0.21 | -2.75 | -11.31 | 5.81 | 0.529 | 0.857 | Imputed |
| rs2738666 | 77057410 | T | A | 0.18 | -1.28 | -10.25 | 7.70 | 0.781 | 0.714 | Imputed |
| rs2738673 | 77058753 | G | A | 0.25 | -1.35 | -9.33 | 6.64 | 0.741 | 0.800 | Imputed |
| rs2667543 | 77059477 | C | T | 0.25 | -1.35 | -9.33 | 6.64 | 0.741 | 0.800 | Imputed |
| rs9928087 | 77059778 | A | C | 0.14 | -0.20 | -10.07 | 9.66 | 0.968 | 1.000 | Imputed |
| rs2667546 | 77060074 | G | C | 0.25 | -1.35 | -9.33 | 6.64 | 0.741 | 0.800 | Imputed |
| rs2667547 | 77060170 | A | G | 0.25 | -1.35 | -9.33 | 6.64 | 0.741 | 0.800 | Genotyped |
| rs2738674 | 77060238 | T | C | 0.25 | -1.35 | -9.33 | 6.64 | 0.741 | 0.800 | Imputed |
| rs2738675 | 77060570 | A | G | 0.11 | -2.24 | -13.07 | 8.59 | 0.686 | 0.688 | Imputed |
| rs9933282 | 77061049 | T | C | 0.14 | -0.20 | -10.07 | 9.66 | 0.968 | 1.000 | Imputed |
| rs7186413 | 77061189 | G | C | 0.14 | -0.20 | -10.07 | 9.66 | 0.968 | 1.000 | Genotyped |
| rs2247099 | 77061446 | A | G | 0.25 | -1.35 | -9.33 | 6.64 | 0.741 | 0.800 | Genotyped |
| rs17638504 | 77061888 | G | C | 0.14 | -0.20 | -10.07 | 9.66 | 0.968 | 1.000 | Imputed |
| rs2738679 | 77062499 | C | T | 0.25 | -1.35 | -9.33 | 6.64 | 0.741 | 0.800 | Imputed |
| rs2738680 | 77062909 | G | A | 0.25 | -1.43 | -9.42 | 6.56 | 0.726 | 0.800 | Imputed |
| rs2667552 | 77063407 | T | C | 0.21 | 0.17 | -8.38 | 8.71 | 0.970 | 1.000 | Imputed |
| rs16947630 | 77063859 | G | T | 0.21 | 0.05 | -8.49 | 8.59 | 0.991 | 0.857 | Imputed |
| rs9937755 | 77065802 | C | T | 0.14 | 0.32 | -9.60 | 10.24 | 0.950 | 1.000 | Genotyped |
| rs9931884 | 77066163 | C | G | 0.15 | 0.62 | -8.75 | 9.99 | 0.897 | 0.857 | Imputed |
| rs13336293 | 77066858 | T | G | 0.14 | 0.32 | -9.60 | 10.24 | 0.950 | 1.000 | Imputed |
| rs2667555 | 77069538 | T | G | 0.17 | 0.77 | -8.39 | 9.92 | 0.869 | 1.000 | Imputed |
| rs9931636 | 77069589 | C | A | 0.18 | 0.86 | -8.16 | 9.88 | 0.852 | 1.000 | Imputed |
| rs8051225 | 77069814 | C | T | 0.21 | -0.31 | -8.85 | 8.23 | 0.943 | 0.857 | Imputed |
| rs9936255 | 77070041 | G | T | 0.29 | -0.79 | -8.54 | 6.96 | 0.841 | 1.000 | Imputed |
| rs2667556 | 77070054 | G | C | 0.17 | 0.33 | -8.80 | 9.45 | 0.944 | 1.000 | Imputed |
| rs2667557 | 77070692 | T | C | 0.16 | 0.56 | -8.69 | 9.80 | 0.906 | 1.000 | Imputed |
| rs4888804 | 77071161 | C | G | 0.20 | -0.88 | -9.65 | 7.88 | 0.844 | 0.857 | Imputed |
| rs2667558 | 77071569 | G | A | 0.17 | 0.48 | -8.66 | 9.62 | 0.918 | 1.000 | Genotyped |
| rs2738685 | 77071688 | G | C | 0.20 | 1.01 | -7.41 | 9.43 | 0.813 | 0.750 | Genotyped |
| rs2667561 | 77071989 | A | G | 0.20 | 1.01 | -7.41 | 9.43 | 0.813 | 0.750 | Genotyped |
| rs2667562 | 77072659 | G | A | 0.20 | 0.96 | -7.47 | 9.38 | 0.824 | 0.750 | Genotyped |
| rs2738686 | 77072873 | C | G | 0.20 | 0.96 | -7.47 | 9.38 | 0.824 | 0.750 | Imputed |
| rs2738690 | 77074528 | T | G | 0.19 | 0.62 | -8.17 | 9.40 | 0.891 | 1.000 | Genotyped |
| rs2667568 | 77074710 | G | T | 0.19 | 0.62 | -8.17 | 9.40 | 0.891 | 1.000 | Imputed |
| rs2667569 | 77074878 | G | A | 0.19 | 0.62 | -8.17 | 9.40 | 0.891 | 1.000 | Imputed |
| rs2738691 | 77075102 | A | C | 0.19 | 0.62 | -8.17 | 9.40 | 0.891 | 1.000 | Imputed |
| rs2738692 | 77075116 | A | T | 0.19 | 1.56 | -7.30 | 10.43 | 0.730 | 1.000 | Imputed |
| rs2667572 | 77075383 | T | G | 0.19 | 0.62 | -8.17 | 9.40 | 0.891 | 1.000 | Imputed |
| rs2738693 | 77075481 | C | T | 0.19 | 0.62 | -8.17 | 9.40 | 0.891 | 1.000 | Imputed |
| rs2667573 | 77075655 | G | A | 0.19 | 0.62 | -8.17 | 9.40 | 0.891 | 1.000 | Imputed |
| rs2738694 | 77076358 | A | G | 0.19 | 0.69 | -8.07 | 9.46 | 0.877 | 1.000 | Imputed |
| rs2738695 | 77076383 | C | T | 0.19 | 0.69 | -8.07 | 9.46 | 0.877 | 1.000 | Imputed |
| rs2738696 | 77076412 | A | G | 0.19 | 0.69 | -8.07 | 9.46 | 0.877 | 1.000 | Imputed |
| rs16947701 | 77076491 | A | G | 0.12 | 6.07 | -4.02 | 16.15 | 0.239 | 0.373 | Imputed |
| rs1540757 | 77077190 | C | T | 0.24 | 1.94 | -6.07 | 9.94 | 0.636 | 0.857 | Imputed |
| rs2738697 | 77077546 | G | A | 0.21 | 2.38 | -5.97 | 10.72 | 0.577 | 0.714 | Imputed |
| rs2254564 | 77077657 | C | T | 0.20 | 1.10 | -7.28 | 9.48 | 0.797 | 0.750 | Imputed |
| rs2667579 | 77078840 | T | C | 0.20 | 1.10 | -7.28 | 9.48 | 0.797 | 0.750 | Genotyped |
| rs2738706 | 77080114 | C | T | 0.20 | 1.19 | -7.19 | 9.57 | 0.781 | 0.750 | Imputed |
| rs10871351 | 77080407 | G | A | 0.23 | 0.23 | -7.90 | 8.35 | 0.956 | 1.000 | Genotyped |
| rs2738708 | 77080865 | C | T | 0.15 | 4.56 | -4.93 | 14.05 | 0.347 | 0.600 | Genotyped |
| rs4888805 | 77081119 | A | T | 0.15 | 4.56 | -4.93 | 14.05 | 0.347 | 0.600 | Imputed |
| rs2175473 | 77081266 | A | T | 0.15 | 4.56 | -4.93 | 14.05 | 0.347 | 0.600 | Imputed |
| rs2738710 | 77081600 | G | A | 0.15 | 4.41 | -5.08 | 13.89 | 0.363 | 0.600 | Genotyped |
| rs16947728 | 77081831 | A | G | 0.12 | 6.04 | -4.05 | 16.13 | 0.241 | 0.373 | Imputed |
| rs12444091 | 77082641 | T | C | 0.12 | 6.04 | -4.05 | 16.13 | 0.241 | 0.373 | Imputed |
| rs2738712 | 77082670 | C | G | 0.15 | 4.41 | -5.08 | 13.89 | 0.363 | 0.600 | Imputed |
| rs12716855 | 77083313 | A | G | 0.15 | 4.41 | -5.08 | 13.89 | 0.363 | 0.600 | Imputed |
| rs4887965 | 77083622 | G | A | 0.15 | 4.41 | -5.08 | 13.89 | 0.363 | 0.600 | Imputed |
| rs2738716 | 77084505 | T | C | 0.15 | 4.41 | -5.08 | 13.89 | 0.363 | 0.600 | Genotyped |
| rs3115956 | 77084533 | A | C | 0.15 | 4.41 | -5.08 | 13.89 | 0.363 | 0.600 | Imputed |
| rs2738717 | 77084554 | C | G | 0.15 | 4.41 | -5.08 | 13.89 | 0.363 | 0.600 | Imputed |
| rs2738719 | 77084661 | C | A | 0.15 | 4.41 | -5.08 | 13.89 | 0.363 | 0.600 | Imputed |
| rs4888807 | 77085010 | A | G | 0.15 | 4.49 | -5.07 | 14.06 | 0.357 | 0.600 | Imputed |
| rs4888808 | 77085037 | C | A | 0.23 | 0.36 | -7.76 | 8.48 | 0.931 | 1.000 | Imputed |
| rs4888809 | 77085126 | C | T | 0.15 | 4.41 | -5.08 | 13.89 | 0.363 | 0.600 | Imputed |
| rs2978629 | 77085559 | C | G | 0.16 | 4.05 | -5.27 | 13.36 | 0.395 | 0.632 | Imputed |
| rs2859636 | 77085653 | G | C | 0.15 | 4.41 | -5.08 | 13.89 | 0.363 | 0.600 | Imputed |
| rs2738722 | 77086120 | C | T | 0.15 | 4.41 | -5.08 | 13.89 | 0.363 | 0.600 | Imputed |
| rs2738723 | 77086177 | T | G | 0.15 | 4.52 | -4.99 | 14.04 | 0.352 | 0.632 | Imputed |
| rs5019441 | 77086367 | G | A | 0.15 | 4.41 | -5.08 | 13.89 | 0.363 | 0.600 | Imputed |
| rs2738724 | 77087419 | C | T | 0.15 | 4.41 | -5.08 | 13.89 | 0.363 | 0.600 | Imputed |
| rs2738726 | 77087562 | A | G | 0.15 | 4.70 | -4.95 | 14.33 | 0.340 | 0.600 | Imputed |
| rs2667586 | 77087858 | A | C | 0.15 | 4.41 | -5.08 | 13.89 | 0.363 | 0.600 | Imputed |
| rs2667587 | 77087915 | G | C | 0.15 | 4.41 | -5.08 | 13.89 | 0.363 | 0.600 | Imputed |
| rs2667589 | 77088386 | A | C | 0.15 | 4.41 | -5.08 | 13.89 | 0.363 | 0.600 | Genotyped |
| rs2738729 | 77088622 | A | G | 0.15 | 4.41 | -5.08 | 13.89 | 0.363 | 0.600 | Imputed |
| rs3419 | 77089133 | G | T | 0.15 | 4.41 | -5.08 | 13.89 | 0.363 | 0.600 | Imputed |
| rs2738736 | 77092324 | A | C | 0.17 | 1.38 | -7.64 | 10.41 | 0.764 | 1.000 | Imputed |
| rs12931826 | 77092704 | T | A | 0.33 | -0.69 | -8.04 | 6.66 | 0.854 | 1.000 | Imputed |
| rs2245201 | 77092871 | C | G | 0.17 | 1.38 | -7.64 | 10.41 | 0.764 | 1.000 | Imputed |
| rs8062956 | 77094080 | C | G | 0.26 | -2.47 | -10.16 | 5.23 | 0.530 | 0.457 | Imputed |
| rs11150082 | 77094198 | T | C | 0.20 | 0.23 | -8.36 | 8.82 | 0.958 | 1.000 | Genotyped |
| rs12443611 | 77094647 | G | A | 0.27 | -1.00 | -8.63 | 6.64 | 0.799 | 0.714 | Genotyped |
| rs12928065 | 77094975 | T | G | 0.28 | -1.02 | -8.65 | 6.61 | 0.794 | 0.714 | Genotyped |
| rs11863365 | 77096077 | G | A | 0.22 | 1.94 | -6.43 | 10.32 | 0.650 | 0.591 | Imputed |
| rs1877281 | 77096916 | A | G | 0.20 | 0.03 | -8.53 | 8.59 | 0.995 | 1.000 | Imputed |
| rs8054925 | 77097883 | C | T | 0.20 | 0.03 | -8.53 | 8.59 | 0.995 | 1.000 | Imputed |
| rs8055733 | 77097927 | A | C | 0.20 | 0.03 | -8.53 | 8.59 | 0.995 | 1.000 | Imputed |
| rs1882957 | 77098833 | A | T | 0.17 | 1.62 | -7.40 | 10.63 | 0.726 | 0.857 | Imputed |
| rs1882958 | 77098856 | G | T | 0.17 | 1.62 | -7.40 | 10.63 | 0.726 | 0.857 | Imputed |
| rs17706509 | 77099224 | A | C | 0.16 | 1.52 | -7.50 | 10.54 | 0.741 | 0.857 | Imputed |
| rs6564564 | 77101097 | T | C | 0.16 | 1.52 | -7.50 | 10.54 | 0.741 | 0.857 | Genotyped |
| rs4257220 | 77101949 | A | G | 0.28 | -0.33 | -7.91 | 7.26 | 0.933 | 1.000 | Imputed |
| rs11643490 | 77102458 | G | A | 0.26 | -1.89 | -9.61 | 5.83 | 0.632 | 0.591 | Imputed |
| rs11643787 | 77102487 | C | T | 0.28 | -0.33 | -7.91 | 7.26 | 0.933 | 1.000 | Imputed |
| rs1465099 | 77102849 | A | G | 0.22 | 1.69 | -6.65 | 10.02 | 0.692 | 0.857 | Genotyped |
| rs1111683 | 77103726 | C | T | 0.29 | -0.80 | -8.37 | 6.77 | 0.836 | 0.857 | Imputed |
| rs11150083 | 77103839 | G | C | 0.18 | -1.36 | -10.03 | 7.31 | 0.758 | 0.714 | Imputed |
| rs2458031 | 77104310 | T | G | 0.31 | 0.02 | -7.35 | 7.40 | 0.995 | 1.000 | Imputed |
| rs2459109 | 77104979 | G | C | 0.29 | -0.80 | -8.37 | 6.77 | 0.836 | 0.857 | Imputed |
| rs1877284 | 77105156 | G | C | 0.34 | 1.14 | -6.10 | 8.38 | 0.758 | 0.714 | Imputed |
| rs7195479 | 77105219 | A | G | 0.16 | 1.52 | -7.50 | 10.54 | 0.741 | 0.857 | Imputed |
| rs1877285 | 77105299 | C | G | 0.28 | -0.35 | -7.99 | 7.28 | 0.928 | 1.000 | Imputed |
| rs2738741 | 77105882 | A | G | 0.16 | 1.94 | -7.14 | 11.02 | 0.675 | 0.800 | Imputed |
| rs8058087 | 77109997 | C | T | 0.10 | -8.89 | -20.13 | 2.36 | 0.122 | 0.127 | Imputed |
| rs4319778 | 77110455 | A | G | 0.33 | -3.62 | -10.96 | 3.73 | 0.335 | 0.591 | Imputed |
| rs16947913 | 77111719 | A | C | 0.12 | -5.77 | -16.29 | 4.75 | 0.283 | 0.256 | Imputed |
| rs2941954 | 77114143 | A | G | 0.23 | 0.63 | -7.50 | 8.76 | 0.879 | 1.000 | Imputed |
| rs9928512 | 77114500 | T | C | 0.12 | -5.38 | -15.92 | 5.16 | 0.318 | 0.288 | Imputed |
| rs9930467 | 77115098 | A | G | 0.12 | -5.38 | -15.92 | 5.16 | 0.318 | 0.288 | Imputed |
| rs9923611 | 77115270 | G | T | 0.10 | -4.11 | -15.46 | 7.24 | 0.478 | 0.857 | Genotyped |
| rs2738744 | 77115740 | A | G | 0.22 | 0.84 | -7.38 | 9.07 | 0.841 | 0.857 | Imputed |
| rs2941948 | 77117341 | C | G | 0.12 | 5.53 | -4.51 | 15.56 | 0.281 | 0.436 | Imputed |
| rs2978632 | 77117381 | T | C | 0.30 | -1.76 | -9.19 | 5.66 | 0.642 | 0.714 | Imputed |
| rs2738747 | 77118672 | G | C | 0.23 | 0.87 | -7.05 | 8.79 | 0.830 | 0.714 | Imputed |
| rs11150084 | 77119321 | C | G | 0.32 | -2.41 | -9.72 | 4.91 | 0.520 | 0.714 | Imputed |
| rs2859640 | 77121441 | A | T | 0.37 | -3.04 | -10.02 | 3.93 | 0.393 | 0.591 | Imputed |
| rs8051498 | 77121942 | G | A | 0.14 | -7.99 | -17.94 | 1.96 | 0.116 | 0.105 | Imputed |
| rs2859642 | 77124302 | T | C | 0.13 | 9.21 | -0.50 | 18.92 | 0.063 | 0.068 | Imputed |
| rs11150085 | 77124595 | A | G | 0.11 | -3.81 | -14.68 | 7.06 | 0.492 | 0.857 | Genotyped |
| rs2860236 | 77125177 | G | C | 0.21 | 0.69 | -7.59 | 8.96 | 0.871 | 1.000 | Imputed |
| rs2738498 | 77125771 | T | C | 0.23 | 0.96 | -6.95 | 8.88 | 0.812 | 0.750 | Imputed |
| rs2738499 | 77125917 | A | G | 0.21 | 3.33 | -4.90 | 11.55 | 0.428 | 0.857 | Imputed |
| rs2667627 | 77125931 | C | T | 0.19 | 2.53 | -6.13 | 11.19 | 0.567 | 0.857 | Imputed |
| rs2738501 | 77126571 | T | C | 0.23 | 0.96 | -6.95 | 8.88 | 0.812 | 0.750 | Imputed |
| rs2738502 | 77128042 | G | C | 0.30 | -4.26 | -11.56 | 3.05 | 0.254 | 0.500 | Genotyped |
| rs1106217 | 77129743 | C | T | 0.30 | -4.08 | -11.39 | 3.23 | 0.274 | 0.517 | Imputed |
| rs1317575 | 77129756 | G | C | 0.30 | -3.22 | -10.58 | 4.14 | 0.391 | 0.565 | Imputed |
| rs1105314 | 77130043 | A | G | 0.30 | -3.22 | -10.58 | 4.14 | 0.391 | 0.565 | Imputed |
| rs1105313 | 77130110 | A | G | 0.10 | 10.60 | -0.21 | 21.41 | 0.055 | 0.067 | Imputed |
| rs2859645 | 77130383 | C | G | 0.35 | -3.37 | -10.46 | 3.72 | 0.352 | 0.632 | Imputed |
| rs2667632 | 77130805 | A | G | 0.10 | 10.60 | -0.21 | 21.41 | 0.055 | 0.067 | Imputed |
| rs2667634 | 77131129 | C | G | 0.30 | -3.58 | -10.86 | 3.70 | 0.335 | 0.536 | Imputed |
| rs12598440 | 77133608 | C | T | 0.42 | -5.23 | -12.10 | 1.64 | 0.136 | 0.177 | Imputed |
| rs2667646 | 77135083 | C | T | 0.35 | -2.34 | -9.47 | 4.79 | 0.520 | 0.647 | Imputed |
| rs12930620 | 77135779 | C | A | 0.35 | -3.08 | -10.20 | 4.04 | 0.396 | 0.457 | Imputed |
| rs2978621 | 77136101 | C | T | 0.34 | -2.58 | -9.70 | 4.54 | 0.478 | 0.565 | Imputed |
| rs11641880 | 77136401 | G | C | 0.13 | -9.32 | -19.68 | 1.04 | 0.078 | 0.068 | Imputed |
| rs2667648 | 77138639 | T | C | 0.21 | 1.45 | -6.98 | 9.87 | 0.737 | 0.857 | Genotyped |
| rs2667649 | 77138664 | C | T | 0.33 | -2.33 | -9.49 | 4.84 | 0.525 | 0.647 | Imputed |
| rs2667650 | 77138707 | G | A | 0.33 | -2.35 | -9.51 | 4.80 | 0.520 | 0.647 | Imputed |
| rs2738510 | 77141793 | G | A | 0.23 | 1.51 | -6.58 | 9.60 | 0.715 | 0.857 | Imputed |
| rs2458029 | 77146033 | G | A | 0.20 | 2.55 | -5.94 | 11.04 | 0.556 | 0.565 | Imputed |
| rs7203676 | 77148426 | T | C | 0.23 | 2.21 | -5.84 | 10.25 | 0.591 | 0.600 | Imputed |
| rs9923592 | 77149054 | G | A | 0.12 | -1.59 | -12.33 | 9.16 | 0.773 | 0.857 | Imputed |
| rs9933688 | 77149385 | G | C | 0.23 | 5.35 | -2.65 | 13.34 | 0.190 | 0.197 | Imputed |
| rs2459108 | 77150377 | C | G | 0.36 | -2.27 | -9.45 | 4.91 | 0.535 | 0.485 | Imputed |
| rs9925067 | 77153653 | G | C | 0.11 | -1.02 | -12.22 | 10.17 | 0.858 | 1.000 | Imputed |
| rs12930179 | 77154699 | A | C | 0.11 | -1.57 | -12.61 | 9.47 | 0.780 | 0.800 | Imputed |
| rs16948096 | 77154816 | C | T | 0.12 | 7.54 | -2.68 | 17.76 | 0.149 | 0.189 | Imputed |
| rs2738521 | 77155150 | G | A | 0.12 | 7.54 | -2.68 | 17.76 | 0.149 | 0.189 | Imputed |
| rs2673779 | 77155247 | T | C | 0.22 | 3.49 | -4.84 | 11.83 | 0.412 | 0.471 | Imputed |
| rs10438629 | 77156653 | G | C | 0.15 | -1.69 | -11.53 | 8.16 | 0.737 | 0.714 | Imputed |
| rs3853362 | 77162332 | G | T | 0.11 | -1.82 | -12.73 | 9.10 | 0.745 | 0.857 | Imputed |
| rs2346007 | 77164753 | G | A | 0.25 | 2.87 | -5.04 | 10.77 | 0.478 | 0.857 | Imputed |
| rs2943768 | 77170415 | C | A | 0.16 | -2.43 | -11.88 | 7.01 | 0.614 | 0.688 | Imputed |
| rs2941935 | 77171262 | G | C | 0.15 | -0.90 | -10.40 | 8.61 | 0.853 | 1.000 | Imputed |
| rs8056137 | 77171928 | G | C | 0.12 | 8.36 | -1.59 | 18.31 | 0.100 | 0.135 | Imputed |
| rs9938843 | 77171952 | G | A | 0.15 | -0.90 | -10.40 | 8.61 | 0.853 | 1.000 | Imputed |
| rs7197903 | 77172009 | T | C | 0.15 | -0.90 | -10.40 | 8.61 | 0.853 | 1.000 | Genotyped |
| rs9927747 | 77172025 | C | G | 0.15 | -0.81 | -10.38 | 8.76 | 0.869 | 1.000 | Genotyped |
| rs9921093 | 77172410 | G | T | 0.15 | -0.81 | -10.38 | 8.76 | 0.869 | 1.000 | Imputed |
| rs9939269 | 77172473 | G | A | 0.15 | -0.81 | -10.38 | 8.76 | 0.869 | 1.000 | Imputed |
| rs9928840 | 77172531 | T | C | 0.15 | -0.81 | -10.38 | 8.76 | 0.869 | 1.000 | Imputed |
| rs9923563 | 77172961 | C | T | 0.22 | -6.31 | -14.32 | 1.71 | 0.123 | 0.107 | Imputed |
| rs2247294 | 77174477 | C | G | 0.41 | -3.02 | -9.96 | 3.92 | 0.394 | 0.436 | Imputed |
| rs4578663 | 77175432 | A | C | 0.17 | -0.24 | -9.31 | 8.82 | 0.958 | 1.000 | Imputed |
| rs7196526 | 77177225 | G | C | 0.16 | 7.23 | -2.00 | 16.46 | 0.125 | 0.174 | Imputed |
| rs16948137 | 77180189 | G | A | 0.11 | -4.35 | -15.55 | 6.84 | 0.446 | 0.447 | Imputed |
| rs2738545 | 77186821 | G | A | 0.29 | -5.51 | -12.90 | 1.89 | 0.145 | 0.178 | Genotyped |
| rs1828518 | 77192694 | C | T | 0.20 | -6.37 | -14.78 | 2.05 | 0.138 | 0.155 | Imputed |
| rs2667542 | 77193196 | G | A | 0.35 | -9.23 | -16.26 | -2.21 | 0.010 | 0.008 | Imputed |
| rs2287951 | 77197686 | C | G | 0.17 | -5.76 | -14.58 | 3.07 | 0.201 | 0.207 | Imputed |
| rs1126185 | 77199241 | G | A | 0.26 | -6.41 | -14.07 | 1.25 | 0.102 | 0.103 | Imputed |
| rs2548879 | 77200307 | C | A | 0.43 | -5.98 | -12.58 | 0.63 | 0.077 | 0.091 | Genotyped |
| rs2738555 | 77203705 | A | G | 0.43 | -4.77 | -11.40 | 1.86 | 0.159 | 0.149 | Imputed |
| rs2550581 | 77204286 | C | A | 0.43 | -4.80 | -11.43 | 1.84 | 0.157 | 0.149 | Imputed |
| rs2550599 | 77212252 | C | G | 0.42 | -4.41 | -11.02 | 2.20 | 0.191 | 0.175 | Imputed |
| rs2548876 | 77212320 | C | T | 0.17 | -4.19 | -13.01 | 4.64 | 0.353 | 0.313 | Imputed |
| rs2738566 | 77213355 | A | C | 0.18 | -1.94 | -10.49 | 6.60 | 0.656 | 0.857 | Imputed |
| rs2673775 | 77213512 | A | G | 0.43 | -4.98 | -11.62 | 1.67 | 0.143 | 0.120 | Imputed |
| rs2738568 | 77213990 | T | C | 0.32 | -2.91 | -9.75 | 3.94 | 0.406 | 0.400 | Imputed |
| rs2550604 | 77215086 | C | T | 0.42 | -4.77 | -11.46 | 1.92 | 0.162 | 0.166 | Imputed |
| rs2738570 | 77215228 | G | T | 0.41 | -2.87 | -9.70 | 3.97 | 0.412 | 0.318 | Imputed |
| rs2738571 | 77215328 | G | A | 0.28 | -2.28 | -9.48 | 4.92 | 0.534 | 0.565 | Genotyped |
| ***rs2548861*** | ***77215894*** | ***T*** | ***G*** | ***0.37*** | ***-2.42*** | ***-9.39*** | ***4.54*** | ***0.495*** | ***0.591*** | ***Imputed*** |
| rs2550606 | 77217132 | C | G | 0.37 | -2.43 | -9.40 | 4.55 | 0.495 | 0.565 | Imputed |
| rs2738573 | 77217831 | T | C | 0.26 | -2.60 | -9.95 | 4.74 | 0.488 | 0.471 | Imputed |
| rs2738576 | 77218684 | A | C | 0.20 | -0.27 | -8.45 | 7.91 | 0.949 | 1.000 | Imputed |
| rs2738577 | 77218875 | G | A | 0.36 | -2.60 | -9.62 | 4.42 | 0.468 | 0.565 | Imputed |
| rs2550608 | 77219165 | C | G | 0.22 | -2.53 | -10.50 | 5.45 | 0.535 | 0.409 | Imputed |
| rs2548866 | 77220759 | G | A | 0.17 | -5.04 | -13.99 | 3.91 | 0.270 | 0.265 | Imputed |
| rs12598987 | 77222048 | C | G | 0.42 | -4.02 | -10.67 | 2.63 | 0.236 | 0.183 | Imputed |
| rs4622523 | 77222243 | C | G | 0.27 | -0.53 | -8.11 | 7.05 | 0.890 | 0.857 | Imputed |
| rs2550612 | 77222489 | A | G | 0.42 | -3.94 | -10.57 | 2.68 | 0.244 | 0.239 | Imputed |
| rs10514439 | 77223246 | T | A | 0.43 | -3.86 | -10.54 | 2.81 | 0.257 | 0.273 | Imputed |
| rs2550613 | 77223309 | T | G | 0.43 | -3.43 | -10.14 | 3.28 | 0.316 | 0.391 | Imputed |
| rs1981881 | 77223653 | C | A | 0.42 | -3.88 | -10.57 | 2.82 | 0.257 | 0.272 | Genotyped |
| rs1079323 | 77225347 | C | T | 0.13 | -8.54 | -18.71 | 1.63 | 0.100 | 0.107 | Imputed |
| rs1110519 | 77225392 | G | A | 0.42 | -3.58 | -10.21 | 3.05 | 0.291 | 0.318 | Imputed |
| rs12716856 | 77225709 | T | C | 0.11 | 1.59 | -9.31 | 12.48 | 0.776 | 0.857 | Imputed |
| rs2550615 | 77225816 | G | C | 0.13 | -8.03 | -18.22 | 2.15 | 0.123 | 0.123 | Imputed |
| rs7193983 | 77228393 | C | G | 0.25 | -6.34 | -14.07 | 1.40 | 0.109 | 0.103 | Imputed |
| rs7196183 | 77228455 | G | A | 0.26 | -6.28 | -13.91 | 1.34 | 0.107 | 0.120 | Imputed |
| rs2550619 | 77229995 | C | G | 0.42 | -3.45 | -10.08 | 3.19 | 0.309 | 0.318 | Imputed |
| rs2550620 | 77230099 | A | C | 0.42 | -3.45 | -10.08 | 3.19 | 0.309 | 0.318 | Imputed |
| rs6564571 | 77230157 | C | G | 0.33 | -2.46 | -9.62 | 4.71 | 0.502 | 1.000 | Imputed |
| rs2432241 | 77231892 | C | T | 0.42 | -3.45 | -10.08 | 3.19 | 0.309 | 0.318 | Imputed |
| rs2550621 | 77233032 | C | T | 0.42 | -3.45 | -10.08 | 3.19 | 0.309 | 0.318 | Imputed |
| rs9923771 | 77233380 | A | T | 0.24 | -4.48 | -12.40 | 3.45 | 0.269 | 0.242 | Genotyped |
| rs1107455 | 77234640 | G | A | 0.23 | -4.09 | -12.05 | 3.87 | 0.314 | 0.275 | Imputed |
| rs2550626 | 77236228 | C | T | 0.42 | -3.17 | -9.80 | 3.46 | 0.349 | 0.391 | Genotyped |
| rs2548873 | 77236321 | T | G | 0.42 | -3.17 | -9.80 | 3.46 | 0.349 | 0.391 | Imputed |
| rs7189040 | 77236564 | T | G | 0.36 | -2.57 | -9.43 | 4.30 | 0.464 | 0.425 | Imputed |
| rs7190546 | 77236632 | T | C | 0.27 | -4.12 | -11.52 | 3.29 | 0.276 | 0.262 | Imputed |
| rs2881375 | 77236992 | C | T | 0.23 | -4.13 | -12.08 | 3.83 | 0.310 | 0.275 | Imputed |
| rs2738589 | 77238261 | A | G | 0.42 | -3.17 | -9.80 | 3.46 | 0.349 | 0.391 | Imputed |
| rs1124434 | 77238834 | C | T | 0.42 | -3.17 | -9.80 | 3.46 | 0.349 | 0.391 | Imputed |
| rs1124433 | 77239012 | C | T | 0.42 | -3.17 | -9.80 | 3.46 | 0.349 | 0.391 | Imputed |
| rs2738591 | 77239356 | C | T | 0.42 | -3.26 | -9.90 | 3.38 | 0.336 | 0.365 | Imputed |
| rs8046010 | 77239458 | T | C | 0.23 | -3.57 | -11.59 | 4.46 | 0.384 | 0.388 | Imputed |
| rs2548843 | 77239537 | C | A | 0.42 | -3.05 | -9.68 | 3.58 | 0.367 | 0.425 | Imputed |
| rs9931114 | 77240965 | G | T | 0.36 | -2.58 | -9.50 | 4.34 | 0.466 | 0.565 | Imputed |
| rs1574442 | 77241830 | A | G | 0.23 | -3.57 | -11.64 | 4.50 | 0.386 | 0.460 | Imputed |
| rs1107984 | 77241875 | G | A | 0.23 | -3.57 | -11.64 | 4.50 | 0.386 | 0.460 | Imputed |
| rs1125670 | 77248633 | T | C | 0.29 | -4.14 | -11.46 | 3.17 | 0.268 | 0.250 | Imputed |
| rs1125671 | 77248787 | G | A | 0.44 | -3.70 | -10.28 | 2.90 | 0.272 | 0.318 | Imputed |
| rs12929743 | 77251379 | C | G | 0.23 | -4.35 | -12.50 | 3.81 | 0.296 | 0.276 | Genotyped |
| rs1109876 | 77253758 | C | T | 0.38 | 0.53 | -6.53 | 7.58 | 0.884 | 1.000 | Imputed |
| rs11862140 | 77254936 | A | G | 0.12 | -4.55 | -15.23 | 6.12 | 0.404 | 0.352 | Imputed |
| rs11150093 | 77255388 | G | C | 0.14 | -6.00 | -15.62 | 3.63 | 0.223 | 0.177 | Genotyped |
| rs1364295 | 77258490 | T | C | 0.13 | -5.98 | -15.95 | 3.98 | 0.240 | 0.181 | Imputed |
| rs16948273 | 77259908 | T | G | 0.11 | -5.90 | -16.40 | 4.60 | 0.271 | 0.205 | Imputed |
| rs2550647 | 77260129 | C | T | 0.47 | -1.88 | -8.54 | 4.79 | 0.581 | 0.647 | Imputed |
| rs2548836 | 77260157 | A | G | 0.40 | -0.35 | -7.21 | 6.50 | 0.920 | 1.000 | Imputed |
| rs1077963 | 77261700 | C | T | 0.49 | 3.47 | -3.10 | 10.04 | 0.300 | 0.279 | Genotyped |
| rs7194800 | 77263005 | T | C | 0.11 | -6.43 | -17.07 | 4.20 | 0.236 | 0.198 | Imputed |
| rs8054120 | 77263482 | T | C | 0.11 | -6.16 | -16.85 | 4.53 | 0.259 | 0.200 | Imputed |
| rs2194340 | 77264326 | C | T | 0.11 | -5.93 | -16.85 | 4.99 | 0.287 | 0.275 | Imputed |
| rs6564575 | 77268743 | T | C | 0.17 | -4.67 | -13.50 | 4.17 | 0.301 | 0.365 | Imputed |
| rs17708443 | 77270568 | G | C | 0.12 | 10.78 | 0.25 | 21.31 | 0.045 | 0.052 | Imputed |
| rs2550655 | 77271027 | G | A | 0.32 | 0.71 | -6.61 | 8.02 | 0.850 | 0.714 | Imputed |
| rs11150094 | 77272068 | T | G | 0.30 | -8.84 | -16.40 | -1.27 | 0.022 | 0.024 | Genotyped |
| rs2738622 | 77273087 | T | G | 0.17 | -4.58 | -13.38 | 4.22 | 0.308 | 0.244 | Genotyped |
| rs2738625 | 77274178 | G | A | 0.32 | 0.71 | -6.62 | 8.05 | 0.849 | 0.714 | Imputed |
| rs9923705 | 77274362 | A | T | 0.42 | -1.82 | -8.91 | 5.27 | 0.616 | 0.857 | Imputed |
| rs2738627 | 77275453 | G | A | 0.20 | -4.97 | -13.47 | 3.53 | 0.252 | 0.219 | Imputed |
| rs12926298 | 77275528 | G | A | 0.44 | -2.64 | -9.71 | 4.42 | 0.464 | 0.591 | Imputed |
| rs16948307 | 77276096 | C | A | 0.11 | -7.40 | -18.21 | 3.41 | 0.180 | 0.230 | Genotyped |
| rs3751881 | 77276700 | C | T | 0.12 | 9.99 | -0.51 | 20.49 | 0.062 | 0.069 | Imputed |
| rs4888826 | 77286563 | G | T | 0.50 | -1.51 | -8.23 | 5.20 | 0.659 | 0.857 | Imputed |
| rs4887974 | 77288950 | A | C | 0.46 | -2.69 | -9.47 | 4.10 | 0.437 | 0.647 | Imputed |
| rs12917793 | 77290412 | T | A | 0.49 | -3.62 | -10.48 | 3.25 | 0.302 | 0.284 | Imputed |
| rs1110556 | 77291412 | G | A | 0.50 | 2.34 | -4.48 | 9.15 | 0.502 | 0.647 | Imputed |
| rs9927805 | 77293757 | G | A | 0.48 | -1.87 | -8.63 | 4.88 | 0.587 | 0.688 | Genotyped |
| rs1424159 | 77295094 | A | G | 0.50 | -2.15 | -8.94 | 4.65 | 0.536 | 0.800 | Imputed |
| rs2042432 | 77298065 | T | A | 0.50 | -2.03 | -8.74 | 4.68 | 0.554 | 0.714 | Imputed |
| rs7206203 | 77300240 | C | T | 0.50 | 2.15 | -4.64 | 8.94 | 0.535 | 0.647 | Imputed |
| rs11645548 | 77311898 | C | T | 0.22 | -5.03 | -13.09 | 3.02 | 0.221 | 0.373 | Imputed |
| rs4888831 | 77312188 | C | T | 0.24 | 0.86 | -7.25 | 8.96 | 0.836 | 1.000 | Imputed |
| rs7187364 | 77312851 | C | A | 0.24 | -0.42 | -8.46 | 7.62 | 0.919 | 1.000 | Imputed |
| rs11860176 | 77313635 | T | C | 0.20 | 0.08 | -8.47 | 8.64 | 0.985 | 1.000 | Imputed |
| rs9925569 | 77313829 | A | G | 0.42 | -2.61 | -9.46 | 4.24 | 0.455 | 0.714 | Imputed |
| rs1477417 | 77318865 | G | A | 0.21 | -4.74 | -13.05 | 3.56 | 0.264 | 0.373 | Genotyped |
| rs1110560 | 77320323 | T | G | 0.48 | -4.17 | -10.96 | 2.62 | 0.229 | 0.253 | Genotyped |
| rs11643459 | 77355588 | C | T | 0.17 | 4.22 | -4.89 | 13.33 | 0.364 | 0.352 | Genotyped |
| rs7198511 | 77355868 | T | C | 0.17 | 2.97 | -6.19 | 12.13 | 0.525 | 0.447 | Genotyped |
| rs7199640 | 77357683 | G | A | 0.31 | 0.68 | -6.58 | 7.94 | 0.855 | 0.857 | Imputed |
| rs1469134 | 77358975 | G | A | 0.45 | -1.51 | -8.36 | 5.34 | 0.666 | 0.688 | Imputed |
| rs9938250 | 77360196 | G | A | 0.18 | 2.01 | -6.60 | 10.62 | 0.647 | 0.750 | Genotyped |
| rs17709176 | 77360622 | T | C | 0.41 | -0.19 | -7.05 | 6.68 | 0.958 | 1.000 | Genotyped |
| rs7201295 | 77360990 | G | A | 0.42 | -1.31 | -8.18 | 5.57 | 0.710 | 0.800 | Imputed |
| rs12716860 | 77361957 | G | C | 0.11 | -0.13 | -10.85 | 10.58 | 0.980 | 1.000 | Imputed |
| rs13335618 | 77362357 | A | G | 0.12 | -2.99 | -13.40 | 7.43 | 0.574 | 0.471 | Imputed |
| rs2161719 | 77362701 | T | C | 0.11 | -0.13 | -10.85 | 10.58 | 0.980 | 1.000 | Imputed |
| rs1125678 | 77362743 | A | C | 0.14 | -0.61 | -10.25 | 9.03 | 0.902 | 1.000 | Imputed |
| rs4888846 | 77363264 | T | A | 0.29 | -0.20 | -7.49 | 7.08 | 0.957 | 1.000 | Genotyped |
| rs7198930 | 77364570 | G | C | 0.14 | -0.61 | -10.25 | 9.03 | 0.902 | 1.000 | Imputed |
| rs7199334 | 77364735 | G | A | 0.14 | -0.61 | -10.25 | 9.03 | 0.902 | 1.000 | Imputed |
| rs7206356 | 77364757 | T | G | 0.41 | 0.50 | -6.37 | 7.38 | 0.886 | 0.857 | Imputed |
| rs7205435 | 77364835 | C | T | 0.43 | -0.49 | -7.36 | 6.39 | 0.889 | 0.800 | Imputed |
| rs17709200 | 77365271 | G | A | 0.30 | -0.41 | -7.63 | 6.81 | 0.911 | 1.000 | Imputed |
| rs9929519 | 77365884 | A | C | 0.30 | -0.41 | -7.63 | 6.81 | 0.911 | 1.000 | Genotyped |
| rs9928973 | 77366013 | A | G | 0.30 | -0.41 | -7.63 | 6.81 | 0.911 | 1.000 | Imputed |
| rs12445943 | 77366573 | A | G | 0.10 | 0.18 | -10.93 | 11.29 | 0.975 | 1.000 | Imputed |
| rs17642004 | 77366625 | A | G | 0.28 | -0.85 | -8.24 | 6.53 | 0.821 | 0.857 | Genotyped |
| rs9934620 | 77367817 | C | G | 0.28 | -0.85 | -8.24 | 6.53 | 0.821 | 0.857 | Imputed |
| rs9925933 | 77368505 | G | A | 0.28 | -0.61 | -8.11 | 6.89 | 0.873 | 0.857 | Imputed |
| rs9931387 | 77370149 | T | A | 0.28 | -0.62 | -8.14 | 6.90 | 0.872 | 0.857 | Imputed |
| rs10514446 | 77370483 | A | G | 0.11 | 0.78 | -10.05 | 11.61 | 0.888 | 1.000 | Genotyped |
| rs9940973 | 77370512 | G | C | 0.12 | 0.31 | -10.15 | 10.77 | 0.954 | 0.857 | Genotyped |
| rs1477411 | 77371221 | G | A | 0.27 | -0.37 | -7.98 | 7.24 | 0.925 | 0.857 | Imputed |
| rs1477412 | 77371460 | C | G | 0.27 | -0.37 | -7.98 | 7.24 | 0.925 | 0.857 | Imputed |
| rs1477413 | 77371575 | T | C | 0.27 | -0.37 | -7.98 | 7.24 | 0.925 | 0.857 | Imputed |
| rs1477414 | 77371616 | T | C | 0.27 | 0.04 | -7.65 | 7.73 | 0.992 | 1.000 | Imputed |
| rs4035780 | 77371660 | G | A | 0.27 | 0.19 | -7.52 | 7.90 | 0.961 | 1.000 | Imputed |
| rs4887986 | 77371886 | T | G | 0.25 | 1.56 | -6.32 | 9.44 | 0.698 | 0.857 | Imputed |
| rs4887987 | 77371955 | A | G | 0.27 | 0.19 | -7.52 | 7.90 | 0.961 | 1.000 | Imputed |
| rs4888847 | 77372130 | A | G | 0.27 | 0.19 | -7.52 | 7.90 | 0.961 | 1.000 | Imputed |
| rs7185036 | 77383696 | A | G | 0.15 | -0.26 | -10.00 | 9.48 | 0.958 | 1.000 | Imputed |
| rs2737302 | 77384321 | G | C | 0.25 | 4.99 | -3.03 | 13.00 | 0.223 | 0.194 | Imputed |
| rs7198122 | 77384558 | C | T | 0.23 | 5.22 | -3.04 | 13.47 | 0.216 | 0.200 | Imputed |
| rs9940926 | 77384816 | C | T | 0.15 | -1.85 | -11.39 | 7.69 | 0.704 | 0.750 | Imputed |
| rs7197824 | 77384930 | G | C | 0.31 | 4.67 | -2.57 | 11.91 | 0.206 | 0.250 | Imputed |
| rs12448575 | 77385034 | C | A | 0.16 | -2.05 | -11.45 | 7.36 | 0.670 | 0.632 | Imputed |
| rs1110559 | 77385366 | A | G | 0.42 | 1.79 | -5.30 | 8.89 | 0.620 | 0.714 | Imputed |
| rs9936415 | 77386046 | T | C | 0.31 | 5.10 | -2.28 | 12.49 | 0.176 | 0.253 | Imputed |
| rs2737301 | 77386213 | C | T | 0.50 | 1.49 | -5.37 | 8.34 | 0.671 | 0.857 | Imputed |
| rs2737297 | 77387476 | T | C | 0.42 | -6.42 | -13.26 | 0.41 | 0.066 | 0.084 | Imputed |
| rs2737296 | 77387606 | G | C | 0.40 | -6.37 | -13.25 | 0.51 | 0.070 | 0.095 | Imputed |
| rs9932188 | 77387916 | G | A | 0.15 | 8.08 | -1.43 | 17.59 | 0.096 | 0.131 | Imputed |
| rs8063748 | 77388178 | G | C | 0.48 | 1.32 | -5.47 | 8.11 | 0.703 | 0.800 | Imputed |
| rs7184456 | 77388632 | G | C | 0.16 | -0.66 | -10.02 | 8.70 | 0.890 | 1.000 | Imputed |
| rs2737295 | 77389031 | T | C | 0.40 | -6.47 | -13.34 | 0.39 | 0.065 | 0.082 | Imputed |
| rs1118736 | 77390649 | C | T | 0.24 | 8.21 | 0.35 | 16.06 | 0.041 | 0.040 | Imputed |
| rs4888852 | 77390676 | T | A | 0.15 | 7.22 | -2.30 | 16.74 | 0.137 | 0.197 | Imputed |
| rs1118735 | 77390825 | A | G | 0.41 | -6.92 | -13.77 | -0.07 | 0.048 | 0.082 | Imputed |
| rs1118734 | 77390847 | T | A | 0.39 | 2.41 | -4.53 | 9.34 | 0.497 | 0.539 | Imputed |
| rs2216730 | 77390953 | G | T | 0.15 | 4.04 | -5.49 | 13.56 | 0.406 | 0.419 | Imputed |
| rs2737292 | 77391159 | A | G | 0.49 | -5.81 | -12.62 | 0.99 | 0.094 | 0.131 | Imputed |
| rs11860793 | 77391933 | G | T | 0.35 | 3.17 | -3.89 | 10.22 | 0.380 | 0.536 | Imputed |
| rs8050074 | 77392916 | G | C | 0.17 | -0.39 | -9.66 | 8.87 | 0.934 | 1.000 | Imputed |
| rs9931801 | 77394531 | T | G | 0.16 | -0.51 | -10.04 | 9.01 | 0.916 | 1.000 | Imputed |
| rs12443658 | 77395010 | T | C | 0.18 | -1.33 | -10.29 | 7.63 | 0.772 | 1.000 | Imputed |
| rs3817671 | 77395244 | G | A | 0.18 | -1.33 | -10.29 | 7.63 | 0.772 | 1.000 | Imputed |
| rs2293902 | 77395273 | T | C | 0.14 | -2.24 | -12.11 | 7.64 | 0.657 | 0.632 | Imputed |
| rs2293901 | 77395439 | C | A | 0.49 | -2.24 | -8.92 | 4.43 | 0.510 | 0.565 | Imputed |
| rs2293900 | 77395453 | T | G | 0.16 | -0.51 | -10.04 | 9.01 | 0.916 | 1.000 | Imputed |
| rs2293899 | 77395552 | G | C | 0.41 | -6.53 | -13.30 | 0.24 | 0.059 | 0.082 | Genotyped |
| rs2293897 | 77396251 | C | G | 0.16 | -3.26 | -12.75 | 6.23 | 0.501 | 0.436 | Imputed |
| rs6564592 | 77396857 | A | G | 0.25 | 5.21 | -2.62 | 13.05 | 0.193 | 0.189 | Genotyped |
| rs1124808 | 77397304 | C | G | 0.33 | -6.77 | -13.92 | 0.39 | 0.064 | 0.104 | Imputed |
| rs1123882 | 77397336 | C | A | 0.15 | 6.77 | -2.72 | 16.27 | 0.162 | 0.259 | Imputed |
| rs1554978 | 77397618 | G | T | 0.15 | 6.77 | -2.72 | 16.27 | 0.162 | 0.259 | Imputed |
| rs1554977 | 77397829 | G | C | 0.15 | -0.73 | -10.35 | 8.89 | 0.882 | 1.000 | Imputed |
| rs1554976 | 77397924 | T | A | 0.25 | 5.05 | -2.84 | 12.94 | 0.210 | 0.188 | Imputed |
| rs6564594 | 77398246 | G | A | 0.25 | 5.05 | -2.84 | 12.94 | 0.210 | 0.188 | Imputed |
| rs4145519 | 77398567 | A | C | 0.47 | -2.40 | -9.20 | 4.40 | 0.489 | 0.539 | Genotyped |
| rs1554974 | 77399594 | T | C | 0.48 | -2.64 | -9.41 | 4.13 | 0.445 | 0.500 | Genotyped |
| rs7499973 | 77399684 | G | C | 0.18 | 1.99 | -7.00 | 10.98 | 0.664 | 1.000 | Genotyped |
| rs7501059 | 77399749 | T | A | 0.37 | 3.72 | -3.53 | 10.98 | 0.315 | 0.400 | Imputed |
| rs6564595 | 77399849 | T | C | 0.15 | -0.65 | -10.18 | 8.88 | 0.894 | 1.000 | Imputed |
| rs1124595 | 77399937 | C | T | 0.25 | -2.86 | -10.70 | 4.97 | 0.474 | 0.400 | Genotyped |
| rs2293896 | 77400157 | G | T | 0.25 | 3.25 | -4.59 | 11.09 | 0.417 | 0.714 | Genotyped |
| rs1124596 | 77400279 | C | T | 0.48 | -2.97 | -9.73 | 3.79 | 0.389 | 0.471 | Genotyped |
| rs2293894 | 77401017 | G | C | 0.14 | 7.39 | -2.20 | 16.99 | 0.131 | 0.148 | Imputed |
| rs9923322 | 77401370 | A | G | 0.16 | 6.40 | -3.01 | 15.81 | 0.183 | 0.250 | Imputed |
| rs9936644 | 77401443 | G | T | 0.16 | 6.11 | -3.27 | 15.50 | 0.202 | 0.280 | Imputed |
| rs3946180 | 77401485 | A | G | 0.16 | -0.89 | -10.40 | 8.62 | 0.854 | 0.857 | Imputed |
| rs8052915 | 77402331 | C | T | 0.39 | -1.86 | -8.88 | 5.17 | 0.604 | 0.800 | Imputed |
| rs12927430 | 77402643 | A | G | 0.34 | -8.13 | -15.19 | -1.08 | 0.024 | 0.020 | Imputed |
| rs11643767 | 77403298 | C | G | 0.38 | -1.47 | -8.49 | 5.56 | 0.683 | 0.857 | Imputed |
| rs12925461 | 77405456 | C | G | 0.30 | 7.72 | 0.32 | 15.12 | 0.041 | 0.037 | Imputed |
| rs1530 | 77405595 | G | T | 0.40 | 0.25 | -6.72 | 7.23 | 0.943 | 1.000 | Imputed |
| rs6564596 | 77406232 | A | C | 0.18 | 1.73 | -7.23 | 10.69 | 0.706 | 1.000 | Imputed |
| rs7404312 | 77406876 | C | T | 0.49 | 4.03 | -2.74 | 10.79 | 0.244 | 0.323 | Imputed |
| rs7205028 | 77409152 | C | G | 0.13 | 7.07 | -2.99 | 17.14 | 0.169 | 0.272 | Imputed |
| rs6564597 | 77409425 | G | C | 0.13 | 7.07 | -2.99 | 17.14 | 0.169 | 0.272 | Imputed |
| rs12447302 | 77410168 | A | C | 0.19 | -2.70 | -11.43 | 6.02 | 0.544 | 0.436 | Imputed |
| rs12445110 | 77410183 | C | T | 0.19 | -3.18 | -11.90 | 5.54 | 0.475 | 0.425 | Imputed |
| rs12928190 | 77411571 | G | A | 0.22 | 9.88 | 1.87 | 17.89 | 0.016 | 0.017 | Imputed |
| rs7196220 | 77411729 | A | C | 0.30 | 2.99 | -4.38 | 10.36 | 0.427 | 0.485 | Imputed |
| rs7201888 | 77411988 | A | G | 0.13 | 7.20 | -2.89 | 17.28 | 0.162 | 0.250 | Genotyped |
| rs7199947 | 77412029 | G | A | 0.13 | 7.20 | -2.89 | 17.28 | 0.162 | 0.250 | Imputed |
| rs12446194 | 77412372 | G | A | 0.15 | -1.66 | -11.10 | 7.79 | 0.731 | 1.000 | Imputed |
| rs9940043 | 77412718 | C | G | 0.28 | 1.85 | -5.73 | 9.42 | 0.633 | 0.857 | Imputed |
| rs12443743 | 77412830 | A | G | 0.15 | -1.23 | -10.66 | 8.21 | 0.799 | 1.000 | Imputed |
| rs1126341 | 77413062 | A | G | 0.15 | -1.23 | -10.66 | 8.21 | 0.799 | 1.000 | Imputed |
| rs6420407 | 77414081 | C | A | 0.34 | 1.44 | -5.75 | 8.63 | 0.695 | 0.600 | Imputed |
| rs6564599 | 77414237 | G | C | 0.42 | 3.98 | -2.90 | 10.86 | 0.257 | 0.288 | Imputed |
| rs7197765 | 77414462 | A | T | 0.24 | 0.32 | -7.85 | 8.50 | 0.938 | 1.000 | Imputed |
| rs7191005 | 77414547 | G | A | 0.42 | 4.09 | -2.79 | 10.97 | 0.244 | 0.318 | Imputed |
| rs2037961 | 77414800 | C | T | 0.42 | 3.99 | -2.91 | 10.89 | 0.258 | 0.284 | Imputed |
| rs2037960 | 77415313 | G | A | 0.16 | -1.53 | -10.92 | 7.87 | 0.750 | 0.714 | Imputed |
| rs7192635 | 77415860 | T | C | 0.16 | -1.53 | -10.92 | 7.87 | 0.750 | 0.714 | Imputed |
| rs7193003 | 77416061 | C | A | 0.14 | 6.57 | -3.31 | 16.46 | 0.193 | 0.318 | Imputed |
| rs1072247 | 77416144 | G | A | 0.25 | 4.61 | -3.29 | 12.51 | 0.253 | 0.272 | Imputed |
| rs9936829 | 77416631 | A | C | 0.34 | -5.42 | -12.64 | 1.80 | 0.141 | 0.198 | Imputed |
| rs9929243 | 77416735 | C | T | 0.34 | -5.42 | -12.64 | 1.80 | 0.141 | 0.198 | Imputed |
| rs12930924 | 77417091 | A | G | 0.34 | -5.42 | -12.64 | 1.80 | 0.141 | 0.198 | Imputed |
| rs12918472 | 77417161 | C | T | 0.37 | -3.68 | -10.79 | 3.43 | 0.310 | 0.352 | Imputed |
| rs7206468 | 77417999 | T | A | 0.22 | -1.74 | -10.17 | 6.70 | 0.687 | 0.560 | Imputed |
| rs7184196 | 77418249 | A | C | 0.12 | 6.62 | -3.76 | 17.00 | 0.212 | 0.250 | Imputed |
| rs6564600 | 77418281 | T | C | 0.34 | -5.42 | -12.64 | 1.80 | 0.141 | 0.198 | Imputed |
| rs7189479 | 77418436 | A | T | 0.34 | -5.42 | -12.64 | 1.80 | 0.141 | 0.198 | Imputed |
| rs7184760 | 77418516 | A | C | 0.13 | 5.03 | -5.07 | 15.12 | 0.330 | 0.333 | Imputed |
| rs7189824 | 77418631 | G | T | 0.16 | -1.53 | -10.92 | 7.87 | 0.750 | 0.714 | Imputed |
| rs8043939 | 77418775 | A | C | 0.15 | -1.13 | -10.55 | 8.29 | 0.815 | 1.000 | Genotyped |
| rs8048957 | 77419288 | C | T | 0.12 | 5.90 | -4.42 | 16.22 | 0.263 | 0.328 | Genotyped |
| rs7499843 | 77419701 | A | T | 0.34 | -5.42 | -12.64 | 1.80 | 0.141 | 0.198 | Imputed |
| rs6564603 | 77419863 | G | A | 0.36 | 1.00 | -6.33 | 8.33 | 0.788 | 0.857 | Imputed |
| rs11150104 | 77419886 | T | C | 0.34 | -5.42 | -12.64 | 1.80 | 0.141 | 0.198 | Genotyped |
| rs11150105 | 77419998 | T | C | 0.34 | -5.42 | -12.64 | 1.80 | 0.141 | 0.198 | Imputed |
| rs1079635 | 77421116 | C | T | 0.34 | -5.40 | -12.61 | 1.81 | 0.143 | 0.213 | Imputed |
| rs6564604 | 77421147 | A | G | 0.12 | 6.44 | -3.92 | 16.80 | 0.223 | 0.250 | Imputed |
| rs9941131 | 77421314 | A | G | 0.46 | -2.13 | -9.12 | 4.86 | 0.550 | 0.857 | Imputed |
| rs6420409 | 77421796 | T | C | 0.12 | 6.24 | -4.07 | 16.56 | 0.236 | 0.296 | Imputed |
| rs8051656 | 77423251 | T | C | 0.27 | 2.66 | -5.16 | 10.48 | 0.505 | 0.857 | Imputed |
| rs7404730 | 77423875 | C | G | 0.48 | 2.55 | -4.39 | 9.48 | 0.472 | 0.714 | Imputed |
| rs8056057 | 77424158 | C | G | 0.32 | 0.56 | -6.61 | 7.72 | 0.879 | 1.000 | Imputed |
| rs13338670 | 77424513 | G | C | 0.23 | -0.17 | -8.32 | 7.99 | 0.968 | 0.800 | Imputed |
| rs1554983 | 77425340 | T | C | 0.49 | -0.69 | -7.38 | 6.00 | 0.839 | 1.000 | Genotyped |
| rs1554981 | 77425610 | T | C | 0.24 | 1.35 | -6.54 | 9.25 | 0.737 | 0.688 | Imputed |
| rs1126340 | 77425769 | A | G | 0.30 | 5.38 | -2.04 | 12.81 | 0.156 | 0.158 | Imputed |
| rs12932650 | 77426617 | G | C | 0.34 | -4.77 | -11.75 | 2.22 | 0.181 | 0.135 | Imputed |
| rs4888854 | 77426961 | A | C | 0.50 | -0.50 | -7.21 | 6.21 | 0.884 | 1.000 | Imputed |
| rs4888855 | 77427046 | T | C | 0.30 | 5.42 | -1.98 | 12.83 | 0.152 | 0.142 | Genotyped |
| rs1554979 | 77427254 | T | C | 0.24 | 1.92 | -5.94 | 9.78 | 0.632 | 0.500 | Imputed |
| rs7185014 | 77427473 | T | C | 0.20 | -3.50 | -11.85 | 4.86 | 0.413 | 0.391 | Imputed |
| rs12920972 | 77427741 | T | C | 0.24 | 2.12 | -5.80 | 10.04 | 0.600 | 0.500 | Imputed |
| rs12923682 | 77427902 | C | G | 0.24 | 1.92 | -5.94 | 9.78 | 0.632 | 0.500 | Imputed |
| rs13332126 | 77428556 | G | A | 0.24 | 2.00 | -5.87 | 9.86 | 0.619 | 0.500 | Imputed |
| rs8060300 | 77428930 | C | T | 0.13 | -0.24 | -10.26 | 9.78 | 0.962 | 1.000 | Imputed |
| rs6564607 | 77429337 | C | G | 0.43 | -2.05 | -8.77 | 4.67 | 0.550 | 0.517 | Imputed |
| rs11649397 | 77430360 | A | G | 0.20 | -3.50 | -11.85 | 4.86 | 0.413 | 0.391 | Genotyped |
| rs11150106 | 77432058 | C | T | 0.21 | -4.03 | -12.51 | 4.46 | 0.353 | 0.313 | Imputed |
| *rs7405423* | *77433428* | *T* | *C* | *0.12* | *-11.42* | *-21.61* | *-1.24* | *0.028* | *0.028* | *Imputed* |
| *rs7405283* | *77433901* | *G* | *A* | *0.12* | *-11.42* | *-21.61* | *-1.24* | *0.028* | *0.028* | *Imputed* |
| *rs9922613* | *77434319* | *A* | *T* | *0.12* | *-12.37* | *-22.39* | *-2.35* | *0.016* | *0.017* | *Imputed* |
| *rs4888865* | *77434919* | *C* | *A* | *0.18* | *-11.39* | *-20.04* | *-2.74* | *0.010* | *0.017* | *Imputed* |
| *rs6420411* | *77435119* | *A* | *C* | *0.24* | *-11.75* | *-19.42* | *-4.08* | *0.003* | *0.004* | *Imputed* |
| *rs1543296* | *77436011* | *A* | *G* | *0.24* | *-11.75* | *-19.42* | *-4.08* | *0.003* | *0.004* | *Imputed* |
| *rs7498176* | *77436331* | *G* | *C* | *0.24* | *-11.75* | *-19.42* | *-4.08* | *0.003* | *0.004* | *Genotyped* |
| *rs7501409* | *77439717* | *A* | *G* | *0.23* | *-10.59* | *-18.46* | *-2.73* | *0.008* | *0.013* | *Imputed* |
| *rs12447303* | *77440263* | *C* | *G* | *0.21* | *-4.30* | *-12.76* | *4.16* | *0.319* | *0.339* | *Imputed* |
| *rs7190122* | *77440619* | *A* | *G* | *0.21* | *-4.02* | *-12.48* | *4.44* | *0.352* | *0.409* | *Imputed* |
| *rs11643930* | *77441655* | *C* | *T* | *0.20* | *-1.78* | *-10.62* | *7.05* | *0.692* | *0.714* | *Imputed* |
| *rs4145518* | *77441867* | *G* | *A* | *0.15* | *-8.75* | *-18.06* | *0.55* | *0.066* | *0.054* | *Imputed* |
| rs8060856 | 77442683 | C | A | 0.19 | -1.86 | -10.73 | 7.01 | 0.682 | 0.714 | Imputed |
| rs12149540 | 77443030 | C | A | 0.13 | -1.61 | -11.62 | 8.40 | 0.753 | 0.800 | Imputed |
| rs11150107 | 77443065 | G | T | 0.14 | -8.09 | -17.83 | 1.65 | 0.104 | 0.114 | Imputed |
| rs11640465 | 77443564 | G | C | 0.19 | -1.79 | -10.75 | 7.16 | 0.695 | 0.714 | Imputed |
| rs11645747 | 77443705 | G | A | 0.19 | -1.96 | -10.92 | 7.00 | 0.669 | 0.714 | Imputed |
| rs12448376 | 77444363 | A | T | 0.19 | -1.96 | -10.92 | 7.00 | 0.669 | 0.714 | Imputed |
| rs12444620 | 77444404 | A | G | 0.19 | -1.96 | -10.92 | 7.00 | 0.669 | 0.714 | Imputed |
| rs1125814 | 77445747 | C | A | 0.13 | -3.32 | -13.41 | 6.76 | 0.518 | 0.565 | Imputed |
| rs4888867 | 77449191 | T | G | 0.22 | -6.07 | -14.55 | 2.42 | 0.162 | 0.170 | Imputed |
| rs8054190 | 77454882 | T | C | 0.13 | -3.80 | -13.95 | 6.37 | 0.464 | 0.565 | Imputed |
| rs8054201 | 77454899 | T | C | 0.13 | -3.76 | -13.93 | 6.42 | 0.470 | 0.565 | Imputed |
| rs2737290 | 77455007 | A | C | 0.12 | -1.45 | -11.83 | 8.94 | 0.785 | 0.857 | Imputed |
| rs7197238 | 77455243 | G | A | 0.13 | -3.28 | -13.64 | 7.09 | 0.536 | 0.485 | Imputed |
| rs11643146 | 77456545 | T | C | 0.14 | -9.56 | -19.15 | 0.02 | 0.051 | 0.043 | Imputed |
| rs12448371 | 77456839 | C | T | 0.21 | -3.82 | -12.42 | 4.79 | 0.385 | 0.429 | Imputed |
| rs2656634 | 77457346 | A | G | 0.11 | -0.70 | -11.31 | 9.92 | 0.898 | 1.000 | Imputed |
| rs2142334 | 77459112 | C | G | 0.41 | 3.73 | -3.30 | 10.76 | 0.299 | 0.279 | Imputed |
| rs2737288 | 77459158 | T | C | 0.32 | -6.62 | -14.04 | 0.80 | 0.081 | 0.072 | Imputed |
| rs2178952 | 77459224 | G | A | 0.44 | 5.96 | -0.89 | 12.81 | 0.089 | 0.076 | Imputed |
| rs7194588 | 77459825 | G | A | 0.13 | -2.47 | -12.55 | 7.62 | 0.632 | 1.000 | Imputed |
| rs9926713 | 77460102 | T | C | 0.13 | -3.25 | -13.51 | 7.01 | 0.535 | 1.000 | Genotyped |
| rs8051859 | 77460182 | G | A | 0.50 | -2.98 | -9.84 | 3.88 | 0.394 | 0.309 | Imputed |
| rs1504885 | 77460769 | C | T | 0.40 | 4.58 | -2.38 | 11.55 | 0.197 | 0.189 | Imputed |
| rs2737283 | 77461562 | A | G | 0.40 | 4.58 | -2.38 | 11.55 | 0.197 | 0.189 | Imputed |
| rs2737282 | 77461719 | G | A | 0.40 | 4.58 | -2.38 | 11.55 | 0.197 | 0.189 | Imputed |
| rs2252435 | 77463522 | A | T | 0.40 | 6.69 | -0.20 | 13.59 | 0.058 | 0.055 | Imputed |
| rs17633136 | 77465881 | G | T | 0.17 | -10.82 | -19.93 | -1.71 | 0.020 | 0.029 | Imputed |
| rs1079636 | 77467963 | C | G | 0.35 | 1.39 | -5.56 | 8.34 | 0.695 | 1.000 | Imputed |
| rs1106616 | 77468342 | C | T | 0.34 | 2.45 | -4.54 | 9.44 | 0.492 | 0.333 | Imputed |
| rs1079634 | 77468635 | G | T | 0.35 | 1.34 | -5.59 | 8.27 | 0.705 | 1.000 | Imputed |
| rs2656630 | 77468827 | T | C | 0.35 | 1.34 | -5.59 | 8.27 | 0.705 | 1.000 | Imputed |
| rs2656629 | 77469334 | T | A | 0.35 | 1.67 | -5.23 | 8.57 | 0.635 | 0.447 | Imputed |
| rs11865743 | 77469494 | G | C | 0.12 | -5.73 | -16.33 | 4.88 | 0.290 | 0.313 | Imputed |
| rs2656628 | 77469571 | A | C | 0.34 | 2.17 | -4.82 | 9.16 | 0.543 | 0.429 | Imputed |
| rs2656626 | 77469615 | G | C | 0.34 | 2.17 | -4.82 | 9.16 | 0.543 | 0.429 | Imputed |
| rs11862750 | 77469673 | C | T | 0.12 | -5.33 | -15.97 | 5.30 | 0.326 | 0.425 | Imputed |
| rs2656624 | 77470231 | A | G | 0.35 | 1.34 | -5.59 | 8.27 | 0.705 | 1.000 | Imputed |
| rs2656623 | 77470496 | G | A | 0.35 | 1.34 | -5.59 | 8.27 | 0.705 | 1.000 | Imputed |
| rs2656622 | 77470665 | G | C | 0.35 | 1.34 | -5.59 | 8.27 | 0.705 | 1.000 | Genotyped |
| rs2656621 | 77470816 | A | G | 0.35 | 1.34 | -5.59 | 8.27 | 0.705 | 1.000 | Genotyped |
| rs2656620 | 77470888 | A | C | 0.36 | 0.99 | -5.91 | 7.90 | 0.778 | 0.857 | Imputed |
| rs2656619 | 77470962 | A | G | 0.35 | 1.34 | -5.59 | 8.27 | 0.705 | 1.000 | Genotyped |
| rs9923225 | 77471501 | A | C | 0.23 | -0.04 | -7.97 | 7.89 | 0.992 | 1.000 | Imputed |
| rs16948720 | 77471812 | A | T | 0.13 | -3.76 | -13.97 | 6.44 | 0.470 | 0.536 | Imputed |
| rs7404729 | 77473155 | A | G | 0.12 | -5.80 | -16.52 | 4.91 | 0.289 | 0.296 | Imputed |
| rs7404742 | 77473227 | A | G | 0.12 | -5.66 | -16.37 | 5.04 | 0.300 | 0.304 | Imputed |
| rs4435265 | 77473661 | G | A | 0.12 | -5.66 | -16.37 | 5.04 | 0.300 | 0.304 | Imputed |
| rs17706982 | 77476484 | G | C | 0.35 | 1.89 | -5.06 | 8.84 | 0.594 | 0.471 | Imputed |
| rs4887990 | 77478402 | G | A | 0.35 | 1.94 | -5.05 | 8.92 | 0.587 | 0.436 | Imputed |
| rs4887991 | 77478564 | G | A | 0.36 | 1.36 | -5.61 | 8.32 | 0.702 | 0.560 | Imputed |
| rs10492909 | 77480575 | G | A | 0.21 | -0.11 | -8.51 | 8.29 | 0.979 | 1.000 | Imputed |
| rs11861122 | 77480823 | G | A | 0.22 | 0.02 | -8.09 | 8.13 | 0.996 | 1.000 | Imputed |
| rs12926355 | 77481326 | G | T | 0.22 | 0.02 | -8.09 | 8.13 | 0.996 | 1.000 | Imputed |
| rs11639743 | 77483496 | T | G | 0.42 | -0.93 | -7.58 | 5.72 | 0.784 | 0.857 | Imputed |
| rs1124356 | 77486814 | C | A | 0.46 | 3.54 | -3.32 | 10.40 | 0.312 | 0.275 | Imputed |
| rs2174403 | 77486959 | C | T | 0.16 | -7.06 | -16.25 | 2.13 | 0.133 | 0.145 | Imputed |
| rs2292124 | 77487717 | C | G | 0.33 | 2.08 | -4.99 | 9.15 | 0.565 | 0.857 | Genotyped |
| rs12449222 | 77488521 | A | G | 0.45 | 4.15 | -2.68 | 10.97 | 0.234 | 0.209 | Genotyped |
| rs11150110 | 77489180 | T | C | 0.45 | 4.15 | -2.68 | 10.97 | 0.234 | 0.209 | Imputed |
| rs8047431 | 77489323 | A | C | 0.37 | 0.79 | -6.33 | 7.91 | 0.828 | 0.714 | Imputed |
| rs7192392 | 77489879 | A | G | 0.34 | 2.17 | -4.93 | 9.27 | 0.550 | 0.436 | Imputed |
| rs9939235 | 77489915 | C | T | 0.39 | 1.39 | -5.63 | 8.40 | 0.698 | 0.800 | Imputed |
| rs8052368 | 77490585 | C | G | 0.45 | 4.15 | -2.68 | 10.97 | 0.234 | 0.209 | Imputed |
| rs2062894 | 77490988 | C | T | 0.46 | 3.39 | -3.45 | 10.24 | 0.332 | 0.323 | Imputed |
| rs2062895 | 77491002 | G | A | 0.17 | -7.83 | -16.76 | 1.10 | 0.086 | 0.089 | Imputed |
| rs11864605 | 77493231 | A | G | 0.14 | -4.97 | -14.79 | 4.84 | 0.321 | 0.383 | Imputed |
| rs2047925 | 77493409 | C | G | 0.14 | -5.47 | -15.29 | 4.36 | 0.276 | 0.313 | Imputed |
| rs16948787 | 77493607 | T | G | 0.12 | 3.17 | -7.54 | 13.88 | 0.562 | 0.714 | Imputed |
| rs4888873 | 77494639 | C | T | 0.14 | -5.43 | -15.26 | 4.41 | 0.280 | 0.239 | Imputed |
| rs6564621 | 77496319 | T | A | 0.43 | -1.64 | -8.67 | 5.38 | 0.646 | 0.647 | Imputed |
| rs16948799 | 77497159 | G | A | 0.15 | -5.59 | -15.26 | 4.07 | 0.257 | 0.276 | Imputed |
| rs4888874 | 77497189 | G | A | 0.34 | 1.89 | -5.27 | 9.05 | 0.605 | 0.857 | Imputed |
| rs16948801 | 77497768 | G | C | 0.17 | -5.45 | -14.53 | 3.64 | 0.241 | 0.230 | Imputed |
| rs16948804 | 77497905 | C | G | 0.35 | 0.74 | -6.39 | 7.87 | 0.839 | 0.857 | Imputed |
| rs11861377 | 77498248 | C | T | 0.17 | -6.92 | -16.21 | 2.37 | 0.145 | 0.155 | Imputed |
| rs8053244 | 77498288 | G | T | 0.32 | 2.46 | -4.68 | 9.59 | 0.500 | 0.714 | Imputed |
| rs1014101 | 77502767 | C | T | 0.11 | 5.36 | -5.97 | 16.70 | 0.354 | 0.380 | Imputed |
| rs7189505 | 77506867 | C | G | 0.15 | -8.65 | -18.13 | 0.82 | 0.074 | 0.068 | Imputed |
| rs16948912 | 77507755 | A | G | 0.19 | -4.47 | -12.97 | 4.03 | 0.303 | 0.333 | Genotyped |
| rs1394568 | 77508242 | C | T | 0.21 | -5.27 | -13.54 | 3.01 | 0.213 | 0.273 | Genotyped |
| rs8055573 | 77508554 | T | C | 0.31 | 1.26 | -6.24 | 8.75 | 0.743 | 0.750 | Imputed |
| rs2221434 | 77508627 | A | G | 0.19 | -4.65 | -13.14 | 3.83 | 0.283 | 0.284 | Imputed |
| rs1125630 | 77509264 | C | G | 0.23 | -4.51 | -12.66 | 3.65 | 0.279 | 0.318 | Imputed |
| rs7192452 | 77511517 | T | G | 0.21 | -2.70 | -11.22 | 5.82 | 0.534 | 0.857 | Imputed |
| rs11645638 | 77517824 | T | C | 0.36 | 1.23 | -6.02 | 8.47 | 0.740 | 0.560 | Imputed |
| rs12918324 | 77518684 | G | C | 0.33 | 2.20 | -4.91 | 9.32 | 0.544 | 0.500 | Genotyped |
| rs12930134 | 77523606 | C | T | 0.17 | 6.01 | -3.31 | 15.33 | 0.207 | 0.138 | Imputed |
| rs12924818 | 77523698 | A | G | 0.17 | 6.01 | -3.31 | 15.33 | 0.207 | 0.138 | Genotyped |
| rs12928676 | 77523826 | C | G | 0.17 | 5.41 | -3.90 | 14.72 | 0.255 | 0.253 | Imputed |
| rs8047671 | 77527482 | T | C | 0.45 | 2.16 | -4.46 | 8.78 | 0.523 | 0.400 | Genotyped |
| rs1349 | 77527961 | G | C | 0.45 | 2.24 | -4.41 | 8.88 | 0.509 | 0.409 | Imputed |
| rs2062897 | 77529503 | A | T | 0.46 | 2.00 | -4.69 | 8.70 | 0.558 | 0.429 | Imputed |
| rs12598030 | 77530589 | A | G | 0.45 | 2.57 | -4.17 | 9.32 | 0.455 | 0.365 | Imputed |
| rs12596126 | 77531330 | G | A | 0.46 | 2.07 | -4.64 | 8.78 | 0.546 | 0.409 | Imputed |
| rs12596284 | 77531468 | C | G | 0.46 | 2.07 | -4.64 | 8.78 | 0.546 | 0.409 | Imputed |
| *rs11643794* | *77538855* | *A* | *G* | *0.38* | *-2.32* | *-9.03* | *4.38* | *0.497* | *0.391* | *Imputed* |
| *rs11642227* | *77541138* | *T* | *A* | *0.36* | *-3.21* | *-9.93* | *3.52* | *0.350* | *0.328* | *Imputed* |
| *rs10871357* | *77541221* | *C* | *G* | *0.42* | *4.18* | *-2.70* | *11.07* | *0.234* | *0.189* | *Imputed* |
| *rs4888887* | *77541431* | *T* | *G* | *0.36* | *-3.21* | *-9.93* | *3.52* | *0.350* | *0.328* | *Imputed* |
| *rs1995549* | *77541637* | *C* | *T* | *0.40* | *4.16* | *-2.70* | *11.02* | *0.235* | *0.200* | *Imputed* |
| *rs8063186* | *77542541* | *T* | *C* | *0.50* | *1.56* | *-5.17* | *8.29* | *0.650* | *0.857* | *Imputed* |
| *rs11648482* | *77542574* | *A* | *G* | *0.36* | *-3.21* | *-9.93* | *3.52* | *0.350* | *0.328* | *Imputed* |
| *rs7205567* | *77546564* | *C* | *G* | *0.36* | *1.68* | *-5.22* | *8.56* | *0.634* | *0.750* | *Imputed* |
| *rs16949032* | *77546850* | *G* | *A* | *0.11* | *-0.33* | *-11.01* | *10.34* | *0.951* | *0.857* | *Imputed* |
| *rs17709129* | *77546962* | *G* | *C* | *0.38* | *-2.72* | *-9.46* | *4.02* | *0.429* | *0.351* | *Genotyped* |
| *rs17709147* | *77548087* | *A* | *T* | *0.11* | *-0.60* | *-11.30* | *10.10* | *0.912* | *0.857* | *Imputed* |
| *rs17635945* | *77548287* | *G* | *A* | *0.11* | *-0.60* | *-11.30* | *10.10* | *0.912* | *0.857* | *Imputed* |
| *rs11150119* | *77548544* | *A* | *G* | *0.46* | *-1.79* | *-8.43* | *4.86* | *0.599* | *1.000* | *Imputed* |
| *rs9319530* | *77549079* | *G* | *C* | *0.50* | *-1.51* | *-8.23* | *5.22* | *0.661* | *1.000* | *Imputed* |
| *rs13334115* | *77549296* | *G* | *C* | *0.37* | *2.19* | *-4.54* | *8.93* | *0.523* | *0.688* | *Imputed* |
| *rs10871358* | *77550043* | *G* | *A* | *0.50* | *-0.32* | *-7.05* | *6.42* | *0.927* | *1.000* | *Imputed* |
| *rs12596756* | *77550293* | *T* | *A* | *0.50* | *0.45* | *-6.29* | *7.19* | *0.896* | *1.000* | *Imputed* |
| *rs12925319* | *77550925* | *G* | *C* | *0.37* | *-2.81* | *-9.55* | *3.93* | *0.414* | *0.352* | *Imputed* |
| *rs16949036* | *77552383* | *G* | *A* | *0.36* | *-3.25* | *-10.05* | *3.55* | *0.349* | *0.288* | *Imputed* |
| *rs12447067* | *77552975* | *C* | *T* | *0.24* | *-0.03* | *-8.12* | *8.07* | *0.995* | *1.000* | *Imputed* |
| *rs9941223* | *77553010* | *C* | *T* | *0.36* | *-2.82* | *-9.56* | *3.91* | *0.411* | *0.352* | *Imputed* |
| *rs7189021* | *77554351* | *G* | *A* | *0.10* | *-5.46* | *-16.47* | *5.55* | *0.331* | *0.328* | *Imputed* |
| *rs17636170* | *77554912* | *G* | *C* | *0.37* | *3.83* | *-2.96* | *10.61* | *0.269* | *0.244* | *Imputed* |
| *rs8052880* | *77555114* | *C* | *T* | *0.38* | *-2.65* | *-9.43* | *4.12* | *0.443* | *0.517* | *Imputed* |
| *rs4600477* | *77555364* | *G* | *A* | *0.37* | *-2.40* | *-9.17* | *4.36* | *0.486* | *0.517* | *Imputed* |
| *rs4888888* | *77556002* | *T* | *C* | *0.12* | *1.09* | *-9.44* | *11.61* | *0.839* | *1.000* | *Imputed* |
| *rs4888889* | *77556085* | *G* | *C* | *0.38* | *-2.38* | *-9.17* | *4.41* | *0.492* | *0.517* | *Imputed* |
| *rs8052893* | *77557050* | *A* | *G* | *0.48* | *-0.38* | *-6.98* | *6.22* | *0.910* | *1.000* | *Imputed* |
| *rs11150120* | *77558265* | *C* | *G* | *0.40* | *-3.02* | *-9.92* | *3.89* | *0.392* | *0.500* | *Imputed* |
| *rs13339083* | *77558424* | *A* | *G* | *0.48* | *-0.49* | *-7.08* | *6.10* | *0.884* | *1.000* | *Imputed* |
| *rs4888892* | *77558492* | *G* | *C* | *0.38* | *-3.06* | *-9.83* | *3.72* | *0.377* | *0.373* | *Imputed* |
| *rs13330742* | *77559187* | *A* | *C* | *0.37* | *1.42* | *-5.36* | *8.21* | *0.681* | *0.750* | *Imputed* |
| *rs16944152* | *77559398* | *C* | *T* | *0.39* | *-3.61* | *-10.38* | *3.16* | *0.296* | *0.328* | *Imputed* |
| *rs2047927* | *77559442* | *G* | *A* | *0.50* | *2.72* | *-4.01* | *9.45* | *0.428* | *0.857* | *Imputed* |
| *rs5024396* | *77559693* | *A* | *G* | *0.47* | *-0.68* | *-7.29* | *5.93* | *0.840* | *1.000* | *Imputed* |
| *rs17636262* | *77559932* | *C* | *G* | *0.37* | *2.20* | *-4.60* | *8.99* | *0.526* | *0.500* | *Genotyped* |
| *rs2062900* | *77560484* | *G* | *A* | *0.37* | *-2.11* | *-9.11* | *4.90* | *0.556* | *0.600* | *Imputed* |
| *rs8057659* | *77561123* | *A* | *G* | *0.49* | *-5.97* | *-12.92* | *0.98* | *0.093* | *0.085* | *Imputed* |
| *rs12716865* | *77561730* | *T* | *C* | *0.35* | *-0.71* | *-7.83* | *6.42* | *0.845* | *1.000* | *Imputed* |
| *rs1467067* | *77561984* | *C* | *A* | *0.32* | *1.93* | *-5.36* | *9.23* | *0.604* | *0.485* | *Imputed* |
| *rs11860206* | *77562298* | *T* | *C* | *0.36* | *-0.64* | *-7.80* | *6.52* | *0.861* | *1.000* | *Imputed* |
| *rs1110891* | *77563088* | *G* | *A* | *0.41* | *5.23* | *-1.69* | *12.15* | *0.139* | *0.137* | *Imputed* |
| *rs1110894* | *77563252* | *T* | *C* | *0.33* | *-2.03* | *-9.44* | *5.39* | *0.592* | *0.857* | *Imputed* |
| rs9930939 | 77567350 | T | C | 0.12 | 6.91 | -3.84 | 17.65 | 0.208 | 0.182 | Imputed |
| rs12918211 | 77567528 | A | T | 0.50 | 3.68 | -2.93 | 10.28 | 0.276 | 0.247 | Genotyped |
| rs1875940 | 77568367 | G | T | 0.49 | -2.81 | -9.69 | 4.08 | 0.425 | 0.600 | Imputed |
| rs7186745 | 77568674 | A | G | 0.13 | 7.31 | -2.70 | 17.32 | 0.153 | 0.149 | Genotyped |
| rs11150125 | 77568753 | C | G | 0.22 | 8.15 | -0.10 | 16.39 | 0.053 | 0.052 | Imputed |
| rs11647122 | 77569510 | C | G | 0.13 | 7.31 | -2.70 | 17.32 | 0.153 | 0.149 | Imputed |
| rs4888001 | 77570533 | T | C | 0.20 | -4.99 | -13.38 | 3.41 | 0.245 | 0.359 | Genotyped |
| rs8063569 | 77570962 | C | G | 0.40 | 1.48 | -5.33 | 8.30 | 0.670 | 0.591 | Imputed |
| rs924870 | 77571088 | T | C | 0.21 | 5.99 | -2.41 | 14.39 | 0.163 | 0.153 | Imputed |
| rs8062872 | 77571223 | G | C | 0.45 | 0.71 | -6.06 | 7.48 | 0.837 | 0.857 | Imputed |
| rs12919788 | 77571413 | G | C | 0.47 | -0.09 | -7.00 | 6.82 | 0.980 | 1.000 | Imputed |
| rs12444526 | 77571963 | C | T | 0.50 | 3.07 | -3.79 | 9.93 | 0.380 | 0.323 | Imputed |
| rs16949121 | 77572043 | T | A | 0.20 | -2.67 | -11.21 | 5.87 | 0.540 | 0.688 | Imputed |
| rs2014980 | 77572260 | G | C | 0.40 | 3.79 | -3.29 | 10.87 | 0.295 | 0.275 | Imputed |
| rs3751834 | 77573909 | G | A | 0.12 | 3.67 | -6.85 | 14.20 | 0.494 | 0.800 | Imputed |
| rs2347080 | 77591484 | C | G | 0.26 | -1.81 | -9.91 | 6.29 | 0.661 | 0.591 | Imputed |
| rs10492905 | 77593245 | G | A | 0.25 | -3.11 | -11.31 | 5.09 | 0.458 | 0.460 | Imputed |
| rs905775 | 77595472 | G | C | 0.24 | -3.87 | -12.14 | 4.41 | 0.361 | 0.333 | Imputed |
| rs2006902 | 77595532 | G | T | 0.25 | -4.74 | -12.92 | 3.43 | 0.256 | 0.200 | Imputed |
| rs13338273 | 77596323 | G | A | 0.25 | -4.63 | -12.81 | 3.55 | 0.267 | 0.211 | Imputed |
| rs13332888 | 77596461 | C | G | 0.25 | -4.43 | -12.64 | 3.78 | 0.291 | 0.273 | Imputed |
| rs13332891 | 77596492 | A | G | 0.25 | -4.63 | -12.81 | 3.55 | 0.267 | 0.211 | Imputed |
| rs9652678 | 77597067 | G | C | 0.15 | -2.54 | -12.31 | 7.23 | 0.611 | 0.857 | Imputed |
| rs9935794 | 77599068 | A | G | 0.19 | -1.04 | -10.09 | 8.02 | 0.823 | 1.000 | Genotyped |
| rs8056452 | 77600054 | A | G | 0.17 | -2.44 | -11.89 | 7.02 | 0.613 | 0.500 | Imputed |
| rs1469135 | 77601455 | C | G | 0.18 | -0.86 | -10.14 | 8.42 | 0.856 | 0.857 | Imputed |
| rs7184417 | 77601614 | T | C | 0.18 | -0.46 | -9.86 | 8.94 | 0.924 | 1.000 | Imputed |
| rs7185147 | 77601679 | G | A | 0.18 | -0.46 | -9.86 | 8.94 | 0.924 | 1.000 | Imputed |
| rs2656659 | 77604802 | A | G | 0.22 | -1.29 | -9.50 | 6.91 | 0.758 | 0.857 | Genotyped |
| rs11645605 | 77604969 | T | C | 0.20 | -4.85 | -13.31 | 3.62 | 0.262 | 0.189 | Imputed |
| *rs12598471* | *77605036* | *T* | *G* | *0.15* | *-10.11* | *-19.59* | *-0.62* | *0.037* | *0.034* | *Imputed* |
| *rs2550725* | *77605477* | *C* | *T* | *0.49* | *-3.79* | *-10.35* | *2.78* | *0.258* | *0.239* | *Genotyped* |
| *rs1862841* | *77606024* | *T* | *A* | *0.35* | *1.27* | *-5.77* | *8.31* | *0.724* | *0.800* | *Imputed* |
| *rs16949214* | *77606478* | *T* | *C* | *0.15* | *-9.83* | *-19.29* | *-0.38* | *0.042* | *0.034* | *Imputed* |
| *rs2550724* | *77606678* | *T* | *G* | *0.34* | *1.46* | *-5.58* | *8.49* | *0.685* | *0.800* | *Imputed* |
| *rs12447246* | *77606842* | *C* | *A* | *0.30* | *0.87* | *-6.45* | *8.19* | *0.816* | *0.857* | *Imputed* |
| *rs2550723* | *77606893* | *C* | *G* | *0.34* | *1.09* | *-5.95* | *8.12* | *0.762* | *0.800* | *Imputed* |
| *rs16949222* | *77607617* | *T* | *C* | *0.15* | *-9.95* | *-19.27* | *-0.63* | *0.037* | *0.037* | *Genotyped* |
| *rs2113305* | *77609498* | *T* | *G* | *0.28* | *-7.61* | *-15.21* | *-0.01* | *0.050* | *0.039* | *Imputed* |
| *rs7185820* | *77610444* | *G* | *A* | *0.18* | *1.92* | *-7.08* | *10.92* | *0.676* | *0.857* | *Imputed* |
| *rs8064141* | *77610545* | *A* | *G* | *0.44* | *6.26* | *-0.60* | *13.12* | *0.074* | *0.073* | *Imputed* |
| *rs16949238* | *77611012* | *A* | *G* | *0.17* | *2.10* | *-6.98* | *11.18* | *0.651* | *0.857* | *Imputed* |
| *rs16949240* | *77611111* | *A* | *G* | *0.17* | *2.00* | *-7.03* | *11.02* | *0.664* | *0.857* | *Imputed* |
| *rs2656653* | *77611413* | *T* | *C* | *0.29* | *-7.84* | *-15.27* | *-0.42* | *0.039* | *0.034* | *Imputed* |
| *rs2550718* | *77611507* | *A* | *C* | *0.29* | *-7.88* | *-15.20* | *-0.56* | *0.035* | *0.031* | *Imputed* |
| *rs2550717* | *77611521* | *A* | *G* | *0.44* | *6.79* | *-0.08* | *13.67* | *0.053* | *0.053* | *Imputed* |
| *rs2550716* | *77612599* | *A* | *G* | *0.29* | *-7.95* | *-15.27* | *-0.63* | *0.034* | *0.031* | *Imputed* |
| *rs16949251* | *77613249* | *G* | *A* | *0.18* | *5.01* | *-3.90* | *13.93* | *0.271* | *0.209* | *Imputed* |
| *rs8047597* | *77614257* | *A* | *G* | *0.47* | *-3.95* | *-10.73* | *2.83* | *0.254* | *0.200* | *Genotyped* |
| *rs2550711* | *77614399* | *G* | *A* | *0.29* | *-8.53* | *-15.84* | *-1.23* | *0.022* | *0.018* | *Genotyped* |
| *rs8047300* | *77614450* | *C* | *A* | *0.28* | *-8.39* | *-15.78* | *-1.00* | *0.026* | *0.021* | *Imputed* |
| *rs2550710* | *77614645* | *A* | *C* | *0.29* | *-7.84* | *-15.22* | *-0.46* | *0.038* | *0.036* | *Genotyped* |
| *rs7199119* | *77614929* | *T* | *A* | *0.32* | *-5.77* | *-13.04* | *1.51* | *0.121* | *0.098* | *Genotyped* |
| *rs1808447* | *77616501* | *T* | *C* | *0.28* | *-8.66* | *-16.16* | *-1.17* | *0.024* | *0.018* | *Imputed* |
| *rs905781* | *77616627* | *G* | *C* | *0.13* | *-7.89* | *-17.66* | *1.88* | *0.114* | *0.090* | *Imputed* |
| *rs8053936* | *77617158* | *T* | *C* | *0.43* | *5.14* | *-1.77* | *12.06* | *0.146* | *0.152* | *Imputed* |
| *rs2550702* | *77618744* | *C* | *A* | *0.28* | *-8.66* | *-16.16* | *-1.17* | *0.024* | *0.018* | *Imputed* |
| *rs2550701* | *77618863* | *C* | *G* | *0.44* | *-2.96* | *-9.74* | *3.82* | *0.392* | *0.309* | *Imputed* |
| *rs2656649* | *77619030* | *G* | *A* | *0.14* | *-3.77* | *-13.20* | *5.66* | *0.433* | *0.436* | *Imputed* |
| *rs11645630* | *77619102* | *A* | *C* | *0.45* | *-11.14* | *-18.21* | *-4.07* | *0.002* | *0.002* | *Imputed* |
| *rs16949276* | *77619263* | *A* | *G* | *0.11* | *9.22* | *-1.47* | *19.91* | *0.091* | *0.092* | *Imputed* |
| *rs2656647* | *77620219* | *G* | *A* | *0.14* | *-3.77* | *-13.20* | *5.66* | *0.433* | *0.436* | *Genotyped* |
| *rs2656646* | *77620360* | *C* | *T* | *0.14* | *-3.77* | *-13.20* | *5.66* | *0.433* | *0.436* | *Imputed* |
| *rs2550698* | *77620657* | *G* | *T* | *0.14* | *-3.77* | *-13.20* | *5.66* | *0.433* | *0.436* | *Genotyped* |
| rs1862840 | 77620757 | C | G | 0.50 | 3.80 | -3.19 | 10.78 | 0.287 | 0.262 | Imputed |
| rs1862839 | 77620818 | G | C | 0.37 | -3.97 | -10.96 | 3.02 | 0.266 | 0.215 | Imputed |
| rs1876976 | 77621174 | G | C | 0.14 | -3.52 | -12.99 | 5.95 | 0.467 | 0.485 | Genotyped |
| rs2656645 | 77621304 | T | C | 0.35 | -4.69 | -11.84 | 2.46 | 0.199 | 0.204 | Imputed |
| rs2550697 | 77621695 | A | T | 0.14 | -3.88 | -13.35 | 5.59 | 0.423 | 0.400 | Genotyped |
| rs12445245 | 77623837 | G | A | 0.13 | -4.80 | -14.50 | 4.90 | 0.332 | 0.313 | Imputed |
| rs2102280 | 77625241 | C | A | 0.26 | -0.65 | -8.43 | 7.13 | 0.871 | 0.647 | Genotyped |
| rs1120114 | 77625818 | T | C | 0.27 | -6.88 | -14.41 | 0.66 | 0.074 | 0.054 | Imputed |
| rs2550694 | 77626238 | C | G | 0.15 | -4.46 | -13.75 | 4.83 | 0.347 | 0.400 | Imputed |
| rs8055815 | 77626307 | G | A | 0.42 | 5.42 | -1.43 | 12.27 | 0.121 | 0.130 | Imputed |
| rs2550692 | 77626862 | G | A | 0.37 | -3.12 | -10.12 | 3.88 | 0.383 | 0.339 | Imputed |
| rs2550691 | 77627177 | T | A | 0.43 | -2.96 | -9.88 | 3.96 | 0.402 | 0.273 | Imputed |
| rs2550690 | 77628479 | G | C | 0.41 | -4.33 | -11.26 | 2.59 | 0.221 | 0.177 | Imputed |
| rs2550689 | 77630453 | G | T | 0.35 | -3.98 | -11.14 | 3.19 | 0.277 | 0.273 | Imputed |
| rs2656614 | 77631530 | C | A | 0.12 | -8.86 | -19.12 | 1.40 | 0.091 | 0.092 | Imputed |
| rs905780 | 77632003 | T | C | 0.12 | -9.25 | -19.63 | 1.14 | 0.082 | 0.079 | Imputed |
| rs16949354 | 77632101 | G | A | 0.10 | -7.77 | -18.62 | 3.08 | 0.161 | 0.207 | Imputed |
| rs2247465 | 77632812 | A | G | 0.16 | -6.12 | -15.29 | 3.05 | 0.191 | 0.165 | Imputed |
| rs2656613 | 77633125 | G | C | 0.23 | -6.66 | -14.73 | 1.42 | 0.106 | 0.132 | Imputed |
| rs2656612 | 77633983 | G | A | 0.18 | -5.68 | -14.40 | 3.05 | 0.203 | 0.198 | Imputed |
| rs9940793 | 77634004 | T | A | 0.12 | 12.23 | 1.60 | 22.85 | 0.024 | 0.032 | Imputed |
| rs2202422 | 77634339 | G | A | 0.18 | -5.68 | -14.40 | 3.05 | 0.203 | 0.198 | Imputed |
| rs1110898 | 77634659 | G | A | 0.25 | -1.79 | -9.77 | 6.19 | 0.660 | 0.591 | Imputed |
| rs2250443 | 77635041 | G | A | 0.20 | -4.99 | -13.59 | 3.61 | 0.256 | 0.215 | Imputed |
| rs1110897 | 77636292 | C | T | 0.18 | -5.96 | -14.68 | 2.75 | 0.181 | 0.198 | Imputed |
| rs16949396 | 77637605 | C | A | 0.18 | -5.65 | -14.35 | 3.06 | 0.204 | 0.207 | Genotyped |
| rs13335371 | 77640271 | G | C | 0.13 | -8.76 | -18.81 | 1.29 | 0.088 | 0.122 | Imputed |
| rs13335415 | 77640369 | G | C | 0.13 | -8.76 | -18.81 | 1.29 | 0.088 | 0.122 | Imputed |
| rs9972825 | 77652446 | T | C | 0.13 | -2.19 | -12.38 | 8.01 | 0.674 | 0.857 | Imputed |
| rs9940608 | 77653349 | T | C | 0.13 | -2.19 | -12.38 | 8.01 | 0.674 | 0.857 | Imputed |
| rs17647978 | 77657555 | C | T | 0.12 | 0.93 | -9.74 | 11.59 | 0.865 | 1.000 | Imputed |
| rs11150132 | 77658957 | A | T | 0.13 | -2.19 | -12.38 | 8.01 | 0.674 | 0.857 | Imputed |
| rs12324967 | 77659211 | T | C | 0.13 | 0.17 | -10.41 | 10.75 | 0.975 | 1.000 | Imputed |
| rs17726834 | 77659614 | T | C | 0.12 | 0.65 | -9.97 | 11.28 | 0.904 | 1.000 | Imputed |
| rs9925128 | 77660324 | T | C | 0.13 | -2.19 | -12.38 | 8.01 | 0.674 | 0.857 | Genotyped |
| rs9930132 | 77662123 | A | G | 0.14 | -1.55 | -11.61 | 8.50 | 0.762 | 1.000 | Imputed |
| rs12149527 | 77668097 | T | C | 0.48 | -3.82 | -10.59 | 2.94 | 0.268 | 0.318 | Imputed |
| rs8049292 | 77669238 | C | G | 0.47 | -3.41 | -10.19 | 3.37 | 0.324 | 0.471 | Imputed |
| rs12926028 | 77669335 | T | C | 0.49 | -2.51 | -9.26 | 4.24 | 0.466 | 0.688 | Imputed |
| rs12924981 | 77669364 | C | G | 0.49 | -2.76 | -9.51 | 4.00 | 0.424 | 0.471 | Imputed |
| rs12716866 | 77669439 | G | C | 0.47 | -3.29 | -10.06 | 3.48 | 0.341 | 0.471 | Imputed |
| rs12925253 | 77669515 | C | G | 0.47 | -3.29 | -10.06 | 3.48 | 0.341 | 0.471 | Imputed |
| rs17648647 | 77669698 | C | A | 0.47 | -3.29 | -10.06 | 3.48 | 0.341 | 0.471 | Imputed |
| rs12102852 | 77671120 | G | A | 0.49 | -2.33 | -9.05 | 4.39 | 0.497 | 0.688 | Imputed |
| rs4888920 | 77673432 | G | A | 0.49 | -2.33 | -9.05 | 4.39 | 0.497 | 0.688 | Imputed |
| rs17727594 | 77673793 | C | G | 0.47 | -3.16 | -9.92 | 3.61 | 0.361 | 0.409 | Imputed |
| rs7199945 | 77674380 | A | G | 0.47 | -3.16 | -9.92 | 3.61 | 0.361 | 0.409 | Imputed |
| rs13339155 | 77676570 | G | A | 0.50 | -1.86 | -8.60 | 4.88 | 0.588 | 0.688 | Genotyped |
| rs9635581 | 77677101 | G | C | 0.11 | -3.60 | -14.50 | 7.30 | 0.517 | 0.457 | Imputed |
| rs17727650 | 77677479 | G | A | 0.47 | -3.20 | -9.95 | 3.56 | 0.354 | 0.409 | Genotyped |
| rs17727687 | 77681077 | T | G | 0.12 | -3.08 | -13.59 | 7.44 | 0.567 | 0.536 | Imputed |
| rs4888924 | 77684051 | A | G | 0.39 | 1.93 | -4.82 | 8.68 | 0.576 | 0.647 | Genotyped |
| rs4888925 | 77684171 | A | G | 0.43 | 2.06 | -4.60 | 8.71 | 0.545 | 0.688 | Genotyped |
| rs11864213 | 77684697 | G | A | 0.41 | 1.53 | -5.28 | 8.35 | 0.659 | 0.800 | Imputed |
| rs9933420 | 77686511 | C | G | 0.11 | -8.03 | -19.27 | 3.21 | 0.162 | 0.165 | Imputed |
| rs12716868 | 77701484 | A | C | 0.19 | 4.18 | -4.24 | 12.60 | 0.331 | 0.365 | Genotyped |
| rs1111230 | 77728638 | A | G | 0.49 | -4.88 | -11.67 | 1.90 | 0.159 | 0.175 | Genotyped |
| rs1424112 | 77729457 | G | A | 0.14 | 2.12 | -7.90 | 12.14 | 0.678 | 0.647 | Imputed |
| rs11647886 | 77730182 | T | G | 0.11 | -0.15 | -11.46 | 11.15 | 0.979 | 0.857 | Imputed |
| rs9938556 | 77730351 | G | C | 0.23 | 2.47 | -5.67 | 10.60 | 0.553 | 0.460 | Imputed |
| rs7200634 | 77733838 | C | A | 0.12 | -2.83 | -13.75 | 8.10 | 0.612 | 0.565 | Genotyped |
| rs7200334 | 77734025 | T | C | 0.31 | 0.85 | -6.52 | 8.23 | 0.820 | 1.000 | Imputed |
| rs12449066 | 77734794 | G | A | 0.12 | -2.55 | -13.23 | 8.13 | 0.640 | 0.857 | Imputed |
| rs9925100 | 77735616 | C | T | 0.34 | 0.85 | -6.20 | 7.91 | 0.812 | 0.857 | Imputed |
| rs7200529 | 77736034 | T | G | 0.34 | 1.22 | -5.85 | 8.29 | 0.736 | 0.750 | Imputed |
| rs7202835 | 77736126 | T | A | 0.34 | 0.96 | -6.21 | 8.12 | 0.794 | 1.000 | Imputed |
| rs8045284 | 77736905 | G | A | 0.36 | -0.34 | -7.40 | 6.73 | 0.926 | 0.857 | Imputed |
| rs8044764 | 77736940 | G | C | 0.36 | -0.34 | -7.40 | 6.73 | 0.926 | 0.857 | Imputed |
| rs8050239 | 77737173 | C | T | 0.36 | -0.34 | -7.40 | 6.73 | 0.926 | 0.857 | Imputed |
| rs11859942 | 77737760 | G | C | 0.29 | 0.40 | -7.10 | 7.90 | 0.918 | 1.000 | Genotyped |
| rs7190436 | 77737948 | C | G | 0.33 | -0.45 | -7.85 | 6.96 | 0.906 | 0.800 | Imputed |
| rs7191404 | 77738424 | C | G | 0.32 | 0.53 | -6.89 | 7.95 | 0.888 | 0.750 | Imputed |
| rs7193157 | 77738614 | G | C | 0.26 | 0.68 | -7.11 | 8.46 | 0.865 | 0.857 | Genotyped |
| rs8056647 | 77739785 | A | G | 0.25 | 0.47 | -7.45 | 8.39 | 0.907 | 0.647 | Imputed |
| rs12050992 | 77739884 | C | T | 0.28 | -0.80 | -8.26 | 6.66 | 0.833 | 0.857 | Imputed |
| rs7188059 | 77740217 | C | T | 0.28 | -0.80 | -8.26 | 6.66 | 0.833 | 0.857 | Imputed |
| rs7204022 | 77740270 | C | G | 0.29 | -0.58 | -7.94 | 6.77 | 0.877 | 0.688 | Imputed |
| rs7184152 | 77740719 | G | A | 0.28 | -0.89 | -8.48 | 6.70 | 0.819 | 1.000 | Imputed |
| rs9924268 | 77740853 | G | A | 0.30 | -0.53 | -7.68 | 6.62 | 0.885 | 0.800 | Imputed |
| rs8046140 | 77740909 | A | G | 0.35 | -0.38 | -7.48 | 6.73 | 0.917 | 0.857 | Imputed |
| rs7190400 | 77741635 | C | A | 0.32 | 0.51 | -6.67 | 7.70 | 0.889 | 1.000 | Imputed |
| rs7190432 | 77741701 | T | A | 0.32 | 0.51 | -6.67 | 7.70 | 0.889 | 1.000 | Imputed |
| rs7194608 | 77741847 | G | C | 0.28 | 1.36 | -6.25 | 8.97 | 0.726 | 1.000 | Imputed |
| rs8053895 | 77741961 | G | A | 0.31 | 0.24 | -7.12 | 7.61 | 0.948 | 1.000 | Genotyped |
| rs8052122 | 77742005 | A | G | 0.31 | 0.24 | -7.12 | 7.61 | 0.948 | 1.000 | Imputed |
| rs4309411 | 77742254 | G | C | 0.32 | 0.45 | -6.82 | 7.72 | 0.904 | 1.000 | Imputed |
| rs4309413 | 77742322 | T | C | 0.32 | 0.45 | -6.82 | 7.72 | 0.904 | 1.000 | Imputed |
| rs4381615 | 77742501 | C | T | 0.32 | 0.45 | -6.82 | 7.72 | 0.904 | 1.000 | Imputed |
| rs4459555 | 77742697 | C | T | 0.32 | 0.23 | -7.06 | 7.52 | 0.950 | 1.000 | Imputed |
| rs4622524 | 77742714 | C | T | 0.32 | 0.22 | -7.07 | 7.51 | 0.953 | 1.000 | Imputed |
| rs4315347 | 77742768 | T | C | 0.32 | 0.22 | -7.07 | 7.51 | 0.953 | 1.000 | Imputed |
| rs7201082 | 77742894 | T | C | 0.32 | 0.22 | -7.07 | 7.51 | 0.953 | 1.000 | Genotyped |
| rs13339141 | 77743269 | G | C | 0.28 | 1.08 | -6.54 | 8.69 | 0.782 | 1.000 | Imputed |
| rs12934227 | 77743453 | G | C | 0.32 | -0.03 | -7.29 | 7.24 | 0.995 | 1.000 | Imputed |
| rs1117007 | 77743697 | C | T | 0.33 | 0.27 | -6.97 | 7.51 | 0.942 | 1.000 | Imputed |
| rs8050187 | 77744387 | C | T | 0.32 | 0.22 | -7.07 | 7.51 | 0.953 | 1.000 | Imputed |
| rs9922411 | 77744426 | C | G | 0.24 | -0.13 | -7.99 | 7.72 | 0.973 | 1.000 | Imputed |
| rs6564651 | 77751639 | T | C | 0.48 | -0.06 | -6.90 | 6.79 | 0.987 | 1.000 | Imputed |
| rs8046352 | 77751761 | G | C | 0.47 | -0.84 | -7.62 | 5.95 | 0.809 | 1.000 | Imputed |
| rs8050958 | 77752025 | G | C | 0.48 | 0.02 | -6.67 | 6.71 | 0.995 | 1.000 | Imputed |
| rs2347567 | 77753693 | C | A | 0.49 | 0.64 | -6.15 | 7.44 | 0.853 | 0.857 | Imputed |
| rs12443788 | 77754099 | C | G | 0.49 | 0.55 | -6.26 | 7.35 | 0.875 | 0.857 | Imputed |
| rs12443833 | 77754290 | C | T | 0.50 | 0.82 | -5.97 | 7.61 | 0.814 | 0.857 | Imputed |
| rs8045067 | 77754805 | A | C | 0.46 | 0.02 | -6.63 | 6.67 | 0.995 | 1.000 | Genotyped |
| rs4888931 | 77756305 | A | G | 0.49 | -0.44 | -7.10 | 6.21 | 0.896 | 1.000 | Imputed |
| rs1110544 | 77756611 | T | C | 0.49 | -0.19 | -6.89 | 6.51 | 0.956 | 1.000 | Imputed |
| rs1110543 | 77756873 | C | T | 0.50 | 0.20 | -6.56 | 6.96 | 0.953 | 0.857 | Imputed |
| rs1110542 | 77756915 | T | A | 0.50 | 0.14 | -6.62 | 6.90 | 0.967 | 0.857 | Imputed |
| rs9972790 | 77757069 | C | G | 0.49 | 0.84 | -5.98 | 7.65 | 0.810 | 0.857 | Imputed |
| rs9972791 | 77757091 | C | G | 0.49 | 0.52 | -6.25 | 7.29 | 0.880 | 0.857 | Imputed |
| rs8051054 | 77757409 | C | T | 0.50 | -0.73 | -7.51 | 6.05 | 0.834 | 0.857 | Imputed |
| rs9972794 | 77757509 | A | G | 0.49 | 0.60 | -6.18 | 7.38 | 0.862 | 0.857 | Imputed |
| rs12051388 | 77757788 | C | T | 0.49 | 0.07 | -6.75 | 6.90 | 0.983 | 1.000 | Imputed |
| rs11150140 | 77757915 | A | G | 0.50 | 0.16 | -6.68 | 7.00 | 0.963 | 1.000 | Imputed |
| rs11150141 | 77757979 | A | G | 0.49 | 0.49 | -6.38 | 7.37 | 0.888 | 0.857 | Imputed |
| rs17656178 | 77758680 | A | G | 0.48 | -0.10 | -6.86 | 6.67 | 0.978 | 1.000 | Imputed |
| rs7203399 | 77759167 | G | A | 0.47 | -0.23 | -6.96 | 6.50 | 0.947 | 1.000 | Imputed |
| rs7205445 | 77759193 | A | G | 0.48 | -0.31 | -7.07 | 6.44 | 0.928 | 1.000 | Imputed |
| rs8046276 | 77760150 | G | C | 0.48 | 0.34 | -6.45 | 7.13 | 0.923 | 1.000 | Imputed |
| rs1116525 | 77760691 | C | T | 0.48 | -1.83 | -8.71 | 5.06 | 0.604 | 0.457 | Imputed |
| rs4888932 | 77762356 | C | G | 0.49 | -1.48 | -8.26 | 5.30 | 0.669 | 0.857 | Imputed |
| rs12920362 | 77762586 | A | T | 0.48 | -0.34 | -7.13 | 6.45 | 0.922 | 1.000 | Imputed |
| rs6564652 | 77762867 | C | G | 0.45 | 0.18 | -6.85 | 7.20 | 0.961 | 1.000 | Imputed |
| rs8055765 | 77764452 | C | T | 0.49 | -1.58 | -8.42 | 5.26 | 0.651 | 0.857 | Genotyped |
| rs6564653 | 77764739 | C | G | 0.49 | -1.73 | -8.56 | 5.09 | 0.619 | 0.500 | Imputed |
| rs1111415 | 77766640 | G | C | 0.49 | -1.42 | -8.24 | 5.40 | 0.684 | 0.857 | Imputed |
| rs1812063 | 77766723 | A | T | 0.49 | -1.39 | -8.14 | 5.36 | 0.686 | 0.857 | Imputed |
| rs2011394 | 77767268 | C | G | 0.49 | -1.33 | -8.12 | 5.46 | 0.702 | 0.857 | Imputed |
| rs1110546 | 77767463 | C | G | 0.49 | -0.92 | -7.72 | 5.88 | 0.791 | 0.857 | Imputed |
| rs2011200 | 77767927 | C | G | 0.50 | -1.70 | -8.40 | 5.00 | 0.619 | 0.632 | Imputed |
| rs1963839 | 77768048 | C | T | 0.50 | -1.99 | -8.69 | 4.71 | 0.561 | 0.539 | Imputed |
| rs2011184 | 77768204 | G | A | 0.50 | -1.91 | -8.62 | 4.79 | 0.576 | 0.632 | Genotyped |
| rs12444578 | 77769120 | A | G | 0.49 | 2.23 | -4.46 | 8.92 | 0.514 | 0.632 | Imputed |
| rs8049330 | 77769305 | A | C | 0.49 | 2.12 | -4.54 | 8.78 | 0.533 | 0.600 | Imputed |
| rs8053844 | 77769396 | T | C | 0.49 | 2.17 | -4.51 | 8.85 | 0.525 | 0.600 | Imputed |
| rs12716871 | 77770740 | C | G | 0.49 | 2.27 | -4.46 | 9.01 | 0.509 | 0.539 | Imputed |
| rs12934392 | 77770836 | A | T | 0.49 | 2.11 | -4.63 | 8.85 | 0.540 | 0.539 | Imputed |
| rs12935684 | 77771615 | A | G | 0.49 | 1.54 | -5.16 | 8.24 | 0.652 | 0.647 | Imputed |
| rs1834037 | 77772586 | C | T | 0.49 | 2.05 | -4.60 | 8.70 | 0.546 | 0.539 | Genotyped |
| rs11860626 | 77773005 | A | G | 0.34 | 1.40 | -5.64 | 8.44 | 0.696 | 0.632 | Imputed |
| rs4888014 | 77773722 | A | G | 0.25 | 0.15 | -7.79 | 8.09 | 0.970 | 0.857 | Imputed |
| rs1424106 | 77774294 | A | G | 0.47 | 1.18 | -5.75 | 8.11 | 0.738 | 0.714 | Imputed |
| rs1424107 | 77774441 | C | G | 0.47 | 1.18 | -5.75 | 8.11 | 0.738 | 0.714 | Imputed |
| rs16949819 | 77777084 | G | A | 0.27 | -1.48 | -9.24 | 6.29 | 0.709 | 0.591 | Imputed |
| rs17730134 | 77777161 | A | G | 0.40 | -0.05 | -7.01 | 6.91 | 0.989 | 1.000 | Imputed |
| rs6564654 | 77778054 | T | G | 0.42 | -1.09 | -7.93 | 5.75 | 0.755 | 0.688 | Genotyped |
| rs1108663 | 77779393 | A | G | 0.40 | -0.66 | -7.50 | 6.19 | 0.851 | 0.857 | Imputed |
| rs446684 | 77779788 | G | A | 0.38 | 3.66 | -3.32 | 10.63 | 0.304 | 0.242 | Imputed |
| rs1110910 | 77780499 | G | C | 0.46 | -2.33 | -9.06 | 4.40 | 0.498 | 0.591 | Imputed |
| rs2017010 | 77780513 | C | G | 0.46 | -2.33 | -9.06 | 4.40 | 0.498 | 0.591 | Genotyped |
| rs436387 | 77782992 | T | C | 0.48 | -0.87 | -7.71 | 5.97 | 0.803 | 1.000 | Imputed |
| rs386497 | 77783493 | T | C | 0.44 | -0.44 | -7.45 | 6.56 | 0.901 | 0.857 | Imputed |
| rs375992 | 77784354 | G | A | 0.23 | 1.42 | -6.76 | 9.60 | 0.734 | 0.714 | Imputed |
| rs7189876 | 77785069 | T | C | 0.20 | -1.02 | -9.93 | 7.89 | 0.822 | 0.750 | Imputed |
| rs378819 | 77785184 | G | A | 0.23 | 1.38 | -6.77 | 9.52 | 0.741 | 0.857 | Imputed |
| rs388512 | 77787235 | G | C | 0.43 | -1.96 | -8.81 | 4.89 | 0.575 | 0.517 | Imputed |
| rs382888 | 77787324 | A | C | 0.36 | -0.60 | -7.66 | 6.46 | 0.867 | 0.750 | Imputed |
| rs385160 | 77787383 | G | C | 0.44 | -0.25 | -7.12 | 6.62 | 0.944 | 0.857 | Imputed |
| rs383673 | 77789947 | A | C | 0.41 | 0.28 | -6.74 | 7.30 | 0.938 | 1.000 | Imputed |
| rs448302 | 77791896 | T | A | 0.43 | -2.19 | -9.04 | 4.65 | 0.530 | 0.517 | Imputed |
| rs424074 | 77792088 | G | A | 0.39 | -1.43 | -8.35 | 5.48 | 0.685 | 0.750 | Imputed |
| rs410233 | 77792188 | T | C | 0.38 | -2.07 | -8.97 | 4.83 | 0.556 | 0.517 | Imputed |
| rs59344 | 77792446 | A | C | 0.39 | -1.74 | -8.65 | 5.17 | 0.622 | 0.539 | Imputed |
| rs398255 | 77792535 | A | G | 0.49 | -3.39 | -10.00 | 3.23 | 0.316 | 0.460 | Imputed |
| rs454120 | 77793305 | A | G | 0.43 | -2.04 | -8.88 | 4.81 | 0.560 | 0.517 | Imputed |
| rs445862 | 77793946 | C | G | 0.42 | -2.81 | -9.69 | 4.08 | 0.425 | 0.436 | Imputed |
| rs400497 | 77794068 | A | C | 0.43 | -2.40 | -9.09 | 4.29 | 0.482 | 0.750 | Imputed |
| rs441004 | 77794331 | A | G | 0.43 | -1.96 | -8.80 | 4.88 | 0.574 | 0.517 | Genotyped |
| rs369487 | 77794409 | T | G | 0.48 | -3.17 | -9.79 | 3.45 | 0.348 | 0.485 | Genotyped |
| rs1813526 | 77795011 | T | C | 0.25 | -5.24 | -13.10 | 2.61 | 0.191 | 0.237 | Genotyped |
| rs870 | 77795088 | G | A | 0.48 | -3.20 | -9.83 | 3.42 | 0.344 | 0.460 | Imputed |
| rs1553723 | 77795109 | C | G | 0.19 | -4.43 | -13.26 | 4.41 | 0.326 | 0.352 | Genotyped |
| rs2016545 | 77795260 | T | C | 0.19 | -4.18 | -13.03 | 4.68 | 0.355 | 0.400 | Imputed |
| rs12935369 | 77795402 | T | C | 0.47 | -0.48 | -7.17 | 6.20 | 0.888 | 1.000 | Imputed |
| rs7203866 | 77795511 | T | C | 0.24 | 0.63 | -7.46 | 8.72 | 0.878 | 1.000 | Imputed |
| rs7197664 | 77795861 | T | C | 0.28 | 2.48 | -5.26 | 10.22 | 0.531 | 0.485 | Imputed |
| rs12927043 | 77796355 | A | C | 0.14 | -2.69 | -12.65 | 7.27 | 0.596 | 0.539 | Genotyped |
| rs442608 | 77796467 | T | C | 0.22 | 2.50 | -5.76 | 10.75 | 0.553 | 0.647 | Imputed |
| rs417711 | 77796873 | A | G | 0.41 | 0.08 | -6.84 | 7.00 | 0.982 | 1.000 | Imputed |
| rs17642520 | 77797327 | C | T | 0.42 | 2.22 | -4.69 | 9.12 | 0.530 | 0.539 | Imputed |
| rs17796342 | 77797365 | T | C | 0.23 | 2.17 | -5.93 | 10.26 | 0.600 | 0.591 | Imputed |
| rs409150 | 77797889 | G | A | 0.47 | 0.77 | -5.98 | 7.52 | 0.823 | 1.000 | Imputed |
| rs384228 | 77797937 | C | A | 0.47 | 0.77 | -5.98 | 7.52 | 0.823 | 1.000 | Imputed |
| rs420196 | 77798109 | C | G | 0.47 | 1.78 | -4.95 | 8.51 | 0.604 | 0.750 | Imputed |
| rs403632 | 77799255 | G | T | 0.23 | 2.24 | -5.85 | 10.33 | 0.587 | 0.591 | Imputed |
| rs421405 | 77799640 | T | A | 0.28 | 4.42 | -3.29 | 12.13 | 0.262 | 0.223 | Imputed |
| rs407083 | 77799945 | T | C | 0.48 | 1.13 | -5.60 | 7.87 | 0.742 | 0.857 | Imputed |
| rs368920 | 77800003 | C | G | 0.48 | 1.13 | -5.60 | 7.87 | 0.742 | 0.857 | Imputed |
| rs450829 | 77800271 | A | G | 0.48 | 1.41 | -5.34 | 8.16 | 0.683 | 0.857 | Imputed |
| rs413263 | 77801308 | G | T | 0.49 | 0.56 | -6.20 | 7.32 | 0.872 | 1.000 | Imputed |
| rs12446313 | 77801523 | G | A | 0.11 | -1.86 | -13.06 | 9.35 | 0.745 | 0.857 | Imputed |
| rs386776 | 77801677 | G | C | 0.35 | 1.72 | -5.51 | 8.95 | 0.641 | 0.591 | Imputed |
| rs438254 | 77801798 | C | T | 0.48 | 1.66 | -5.10 | 8.41 | 0.631 | 0.560 | Imputed |
| rs418017 | 77802646 | T | C | 0.37 | 1.82 | -5.25 | 8.89 | 0.614 | 0.539 | Imputed |
| rs384216 | 77802966 | T | C | 0.47 | 1.41 | -5.36 | 8.17 | 0.684 | 0.647 | Imputed |
| rs383362 | 77803321 | G | T | 0.47 | 1.41 | -5.36 | 8.17 | 0.684 | 0.647 | Imputed |
| rs2288034 | 77803457 | C | G | 0.41 | 3.41 | -3.55 | 10.36 | 0.337 | 0.242 | Imputed |
| rs2288033 | 77803462 | T | C | 0.41 | 3.41 | -3.55 | 10.36 | 0.337 | 0.242 | Imputed |
| rs12828 | 77803824 | G | A | 0.41 | 3.56 | -3.39 | 10.51 | 0.315 | 0.211 | Genotyped |
| rs391870 | 77804036 | T | C | 0.43 | 0.16 | -6.63 | 6.95 | 0.963 | 0.857 | Imputed |
| rs368299 | 77804510 | A | C | 0.37 | 1.91 | -5.17 | 8.99 | 0.598 | 0.539 | Imputed |
| rs395050 | 77804563 | G | A | 0.37 | 1.91 | -5.17 | 8.99 | 0.598 | 0.539 | Genotyped |
| rs409183 | 77804959 | T | G | 0.37 | 2.16 | -4.93 | 9.24 | 0.551 | 0.471 | Imputed |
| rs401994 | 77804989 | C | A | 0.42 | 4.03 | -2.93 | 10.98 | 0.257 | 0.183 | Genotyped |
| rs435617 | 77805091 | G | C | 0.41 | 4.23 | -2.73 | 11.19 | 0.234 | 0.168 | Imputed |
| rs17642789 | 77806843 | G | A | 0.47 | 2.30 | -4.42 | 9.02 | 0.502 | 0.500 | Imputed |

Table S3. Association analysis of the *WWOX* gene markers with triglycerides.

Italic lines represent the four regions described in the text. The rs2548861 polymorphism associated with HDL by Lee et al. (2008) is highlighted in bold italic. A1: minor allele; A2: major allele; MAF: minor allele frequency; BETA: regression coefficient (change of the phenotypic mean associated with each additional copy of the minor allele); L95, U95: lower, upper confidence intervals; eP: empirical p value.
